# Isolation of kinetically-stabilised diarylchalcogenide radical cations

**DOI:** 10.1038/s42004-025-01613-z

**Published:** 2025-08-09

**Authors:** Pascal Komorr, Corina Stoian, Aleksa Radović, Björn A. Meyer, Pim Puylaert, George E. Cutsail III, Emanuel Hupf, Jens Beckmann

**Affiliations:** 1 Institute for Inorganic Chemistry and Crystallography, University of Bremen, Bremen, Germany; 2 Max Planck Institute for Chemical Energy Conversion, Mülheim an der Ruhr, Germany; 3 Department of Chemistry, Ludwig-Maximilians-Universität München, Munich, Germany

**Keywords:** Organometallic chemistry, Electron transfer, Structure elucidation

## Abstract

Chalcogenide radical cations [R_2_E]^•+^ (E = S, Se, Te) are commonly short-lived intermediates of fundamental interest. Sulfide radical cations in particular are associated in vivo with oxidative stress and neuropathological processes. Having succeeded in the preparation of *meta*-terphenyl-based dichalcogenide radical cations [R_2_E_2_]^•+^ (E = S, Se, Te), and a telluride analogue [R_2_Te]^•+^ in the past, we aimed to complete the series regarding sulfur and selenium. Here we report on the single-electron oxidation of diarylchalcogenides M^S^FluindPhE (E = S, Se, Te; M^S^Fluind = dispiro[fluorene-9,3’-(1’,1’,7’,7’-tetramethyl-*s*-hydrindacen-4’-yl)-5’,9”-fluorene]) using XeF_2_ in the presence of K[B(C_6_F_5_)_4_], which afforded deeply coloured and isolable radical cation salts [M^S^FluindPhE][B(C_6_F_5_)_4_] (E = S, Se, Te). Structural and electronic properties were characterised by electron paramagnetic spectroscopy, cyclic voltammetry, optical absorption spectroscopy and single crystal X-ray diffraction (E = Se, Te), combined with extensive quantum mechanical computations.

**Figure:**
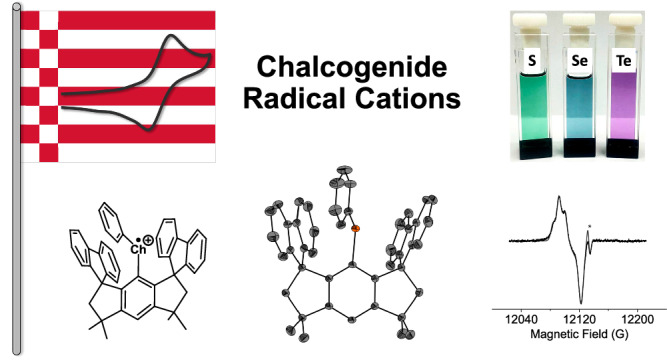

## Abstract (web summary)

Chalcogenide radical cations are usually short-lived intermediates, making their isolation challenging. Here, the authors report the stabilization of these species by using the M^S^Fluind (dispiro[fluorene-9,3’-(1’,1’,7’,7’-tetramethyl-*s*-hydrindacen-4’-yl)-5’,9”-fluorene]) substituent to produce [M^S^FluindPhE][B(C_6_F_5_)_4_] (E = S, Se, Te) radical cation salts, which they characterize using electron paramagnetic spectroscopy, cyclic voltammetry, optical absorption spectroscopy, single crystal X-ray diffraction and quantum mechanical computations.

## Introduction

**Fig. 1:**
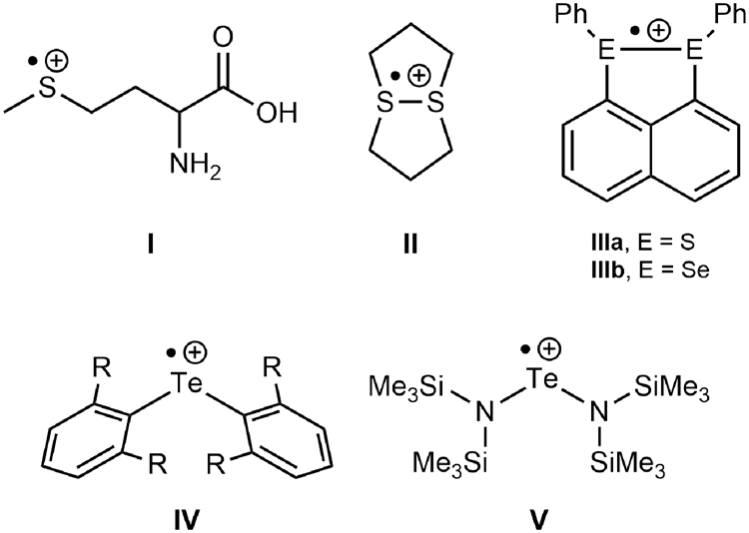
Selected radical cations of chalcogenides known in the literature. While the Methionine and 1,5-dithiacyclooctane radical cations **I**^8,9^, **II**^25^ have been observed spectroscopically, stabilised chalcogen radical cations have been structurally characterised for dichalcogenides (e.g. **IIIa/b**^26,27^), as well as for monotellurides (**IV**^29^, R = mesityl, **V**^28^).

Ever since Gomberg’s seminal discovery of the trityl radical in 1900^1^, odd-electron species of the main group elements received considerable attention^2,3^, which holds particularly true for sulphur-centred radicals^4–6^. Amongst the latter, archetypical diorganosulfide radical cations [R_2_S]^•+^ have been generated in situ by single-electron oxidation, sensitiser-assisted photolysis and gamma radiolysis of the neutral parents R_2_S (R = alkyl, aryl); however, all attempts to isolate them failed due to their extremely short life span^7^. Lots of the interest in this compound class stems from the observation that the methionine radical cation **I**, first characterised by electron paramagnetic resonance (EPR) spectroscopy in 1972^8,9^, when incorporated in proteins, plays a central role for neurodevelopmental disorders, such as autism and schizophrenia, as well as neurodegenerative disorders, such as Alzheimer’s and Parkinson’s diseases (Fig. 1)^10–14^.

Besides various fragmentation pathways, diorganosulfide radical cations undergo (temporary) stabilisation by reaction with their neutral parents R_2_S to give dinuclear radical cations [R_2_SSR_2_]^•+^ possessing three-electron two-centred (3e2c) bonds^15–19^. Other modes of stabilisation, including interactions with N- and O-donor functionalities as well as aromatic π-systems, are also known^20–24^. However, the vast majority of these partially stabilised diorganosulfide radical cations are still highly transient and decompose rapidly into multiple products.

The first exception involves the cyclic 1,5-dithiooctane radical cation **II** reported by Musker’s group in 1976, which possesses a life span of several days, which was attributed to the transannular 3e2c S–S bond (Fig. 1)^25^. The first isolated and fully characterised example of a dinuclear radical cation, namely, the 1,3-bis(phenylsulfanyl)naphthalene radical cation **IIIa** was described by Wang’s group in 2014^26^. The same group also published the analogous selenium radical cation **IIIb** in the same year^27^. While there are no stable mononuclear diorganosulfide radical cations [R_2_S]^•+^ and diorganoselenide radical cations [R_2_Se]^•+^, we are aware of only one example of a diorganotelluride radical cation, [R_2_Te]^•+^, namely, the kinetically-stabilised bis(*m*-terphenyl)telluride radical cation, [(2,6-Mes_2_C_6_H_3_)_2_Te]^•+^ (**IV**), recently published by our group, in which the spin density is situated almost entirely at the tellurium^28^. This compound nicely complemented Roesky’s electronically-stabilised bis[bis(trimethylsilyl)amide]telluride radical cation [{(Me_3_Si)_2_N}_2_Te]^•+^ (**V**) published in 1991, in which electron density resides at the tellurium and nitrogen atoms^29^. Attempts to prepare the sulphide and selenide radical cations analogous to **IV** were met with failure, while cyclic voltammetry studies of the neutral chalcogenides (2,6-Mes_2_C_6_H_3_)_2_E (E = S, Se) confirmed the irreversible nature of the electrochemical oxidation^28^.

Kinetic stabilisation of highly reactive species relies on the judicious choice of bulky and rigid substituents. In 2011, Tamao’s group introduced a family of bulky *s*-hydrindacene-based substituents, including the M^S^Fluind substituent (systematic name = dispiro[fluorene-9,3′-(1′,1′,7′,7′-tetramethyl-*s*-hydrindacen-4′-yl)-5′,9″-fluorene])^30^, which possesses excellent properties for kinetic stabilisation. Starting with the first donor-free phosphenium ions in 2021^31^, the M^S^Fluind ligand and alkyl decorated variations have been instrumental to isolate a wide range of low-valent *p*-block element species of groups 14^32–35^ and 15^36–44^, alongside one example from group 13^45^. Very recently, a singly-bonded tellurenyl radical [R–Te]^•^ could be isolated by Tan’s group^46^. Complementing these results, we now prepared a series of bench-stable diarylchalcogenide radical cations [R_2_E]^•+^ (E = S, Se, Te) with the M^S^Fluind substituent.

## Results and discussion

**Fig. 2:**
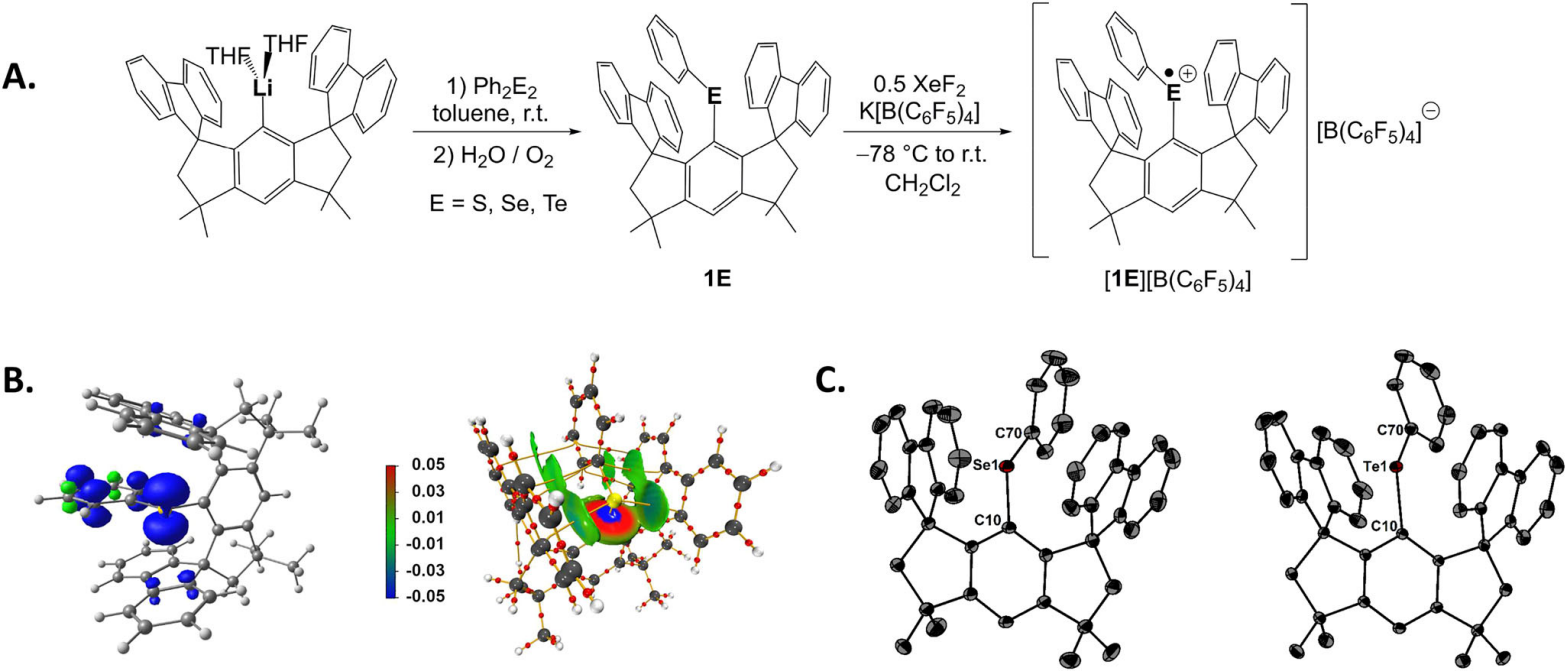
Synthesis and structural characterisation of chalcogen radical cations. **A** Synthesis of **1E** and [**1E**][B(C_6_F_5_)_4_] (E = S, Se, Te). **B** Mulliken spin density (left) of **[1S]**^•+^ (blue—ositive, green—negative, contour value 0.005). AIM molecular graph (right) of **[1S]**^•+^ with bond critical points as red spheres and bond paths in orange, as well as IGM based on a Hirshfeld partition of the molecular density. IGMH *iso*-surfaces at s(r) = 0.005 colour coded with sign(λ2)ρ in a.u. Blue surfaces refer to attractive forces and red to repulsive forces. Green indicates weak interactions. **C** Molecular structures of [**1Se**][B(C_6_F_5_)_4_] and [**1Te**][B(C_6_F_5_)_4_] showing 50% probability ellipsoids and the essential atomic numbering. Hydrogen atoms, solvent molecules and counter-ions are omitted.

**Fig. 3:**
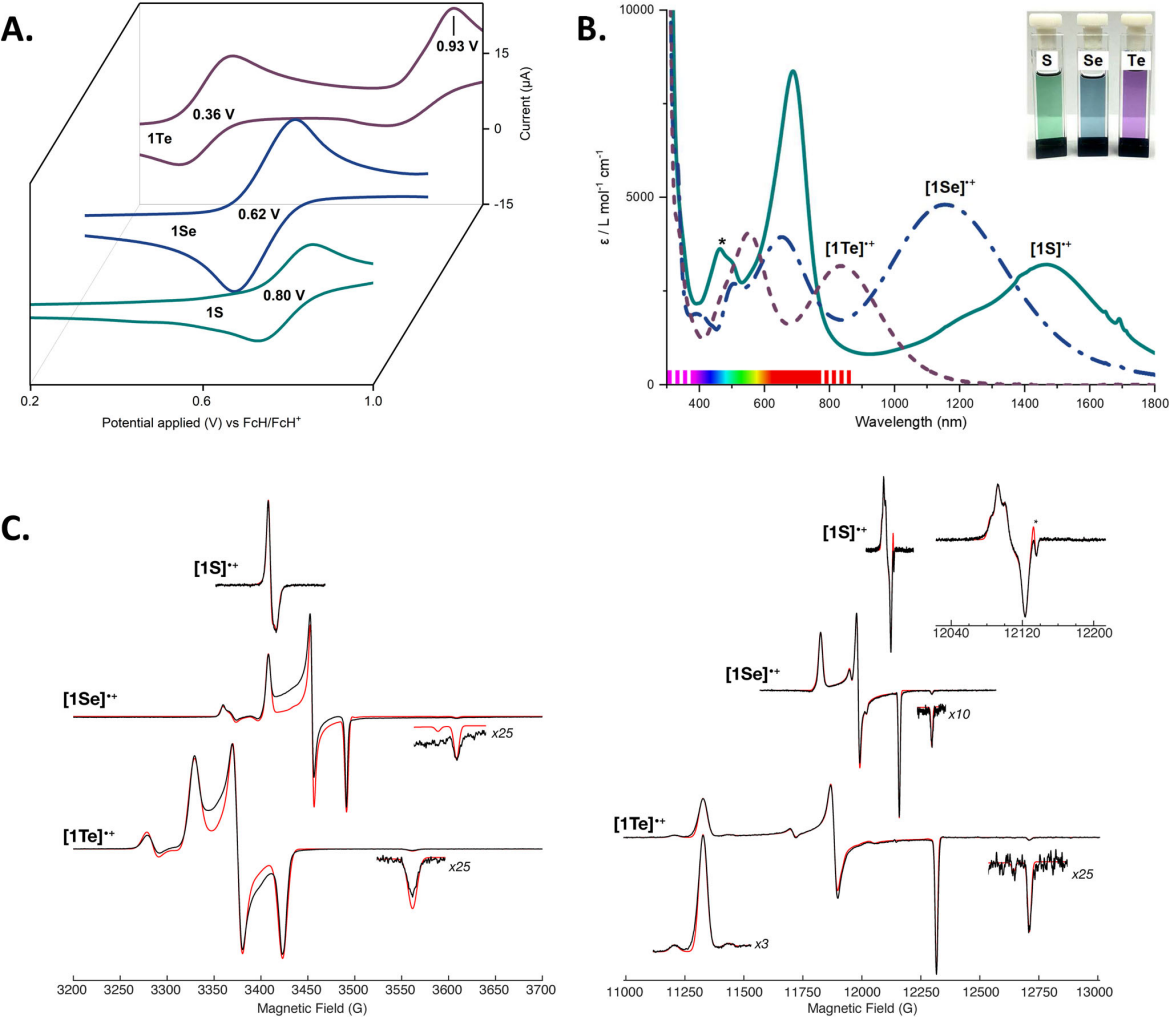
Electronic characterisation of chalcogen radical cations. **A** Cyclic voltammograms of **1E** (E = S, Se, Te) measured in CH_2_Cl_2_ with c(**1E**) = 0.1 mM, c([^*n*^Bu_4_N][PF_6_]) = 0.1 M, scan rate = 100 mV s^−1^ and ferrocene (FcH) as an internal standard. Half-wave potentials E_1/2_ of the 1^st^ oxidations are given, as well as the oxidation peak potential of the 2^nd^ oxidation of **1Te**. **B** UV-Vis-NIR-absorption spectra of solutions of [**1E**][B(C_6_F_5_)_4_] (E = S, Se, Te) in CH_2_Cl_2_ (c = 0.05 mM) (* marks a band attributed to an unidentified impurity). The NIR-absorption bands attributed to HOMO-LUMO-transitions (800–1500 nm) are marked. **C** X-band (left) and Q-band (right) EPR spectra of frozen solutions of [**1E**][B(C_6_F_5_)_4_] (E = S, Se, Te) in CH_2_Cl_2_/THF (1:1) mixture measured at 60, 22, 7.4 K (X-band) and 70, 45, 25 K (Q-band), respectively, displayed on the same magnetic field axis. The figure inset is a magnetic field expansion of [**1S**][B(C_6_F_5_)_4_] to display the axial character of the spectrum. Experimental data is shown in black, while simulations are shown in red. EPR simulation parameters are detailed in Supplementary Table S73 (* marks impurity in the Q-band spectrum ~0.8%).

The reaction of M^S^FluindLi(THF)_2_ (**I**) and diphenyl dichalcogenides Ph_2_E_2_ at room temperature in toluene provided the diarylchalcogenides M^S^FluindPhE (**1E**) as colourless (E = S, Se) or yellow (E = Te) crystals in 53–62% isolated yield (Fig. 2A). The molecular structures of **1E** (E = S, Se, Te) are given in Supplementary Figs. S63–S65. The two heavier diarylchalcogenides **1Se** and **1Te** are characterised by ^77^Se (114 MHz) and ^125^Te (189 MHz) nuclear magnetic resonance (NMR) chemical shifts of *δ* = 314 and 507 ppm, respectively, which are significantly more shielded than those of Ph_2_Se (422 ppm)^47^ and Ph_2_Te (707 ppm)^48^. The electrochemical properties of **1E** (E = S, Se, Te), studied by cyclic voltammetry, revealed reversible, diffusion-controlled events with half-wave potentials at *E*_1/2_ = 0.80, 0.62 and 0.36 V, respectively (vs. FcH/FcH^+^ redox couple, Fig. 3A). When compared to the bis(*m*-terphenyl)chalcogenides (2,6-Mes_2_C_6_H_3_)_2_E (E = S, Se, Te), the oxidation of **1E** (E = S, Se, Te) occurred at lower potentials, thus more readily. Unlike **1S** and **1Se**, (2,6-Mes_2_C_6_H_3_)_2_E (E = S, Se) showed irreversible oxidation processes^28^. Additionally, **1Te** showed a second, irreversible oxidation event at *E*_p,a2_ = 0.93 V, which may be associated with the formation of the corresponding dication in combination with a subsequent chemical reaction.

The diarylchalcogenide radical cations, [**1E**][B(C_6_F_5_)_4_] (E = S, Se, Te), were obtained by single-electron oxidation of **1E** with half an equivalent of XeF_2_ in the presence of K[B(C_6_F_5_)_4_] at −78 °C in CH_2_Cl_2_ (Fig. 2A)^49^. The formation of [**1S**]^•+^ and [**1Te**]^•+^ readily occurred upon warming to room temperature and was accompanied by a colour change to dark blue and dark purple, respectively. For [**1Se**]^•+^ the formation appeared to be incomplete, as indicated by a yellow to green colour. In this case, slow diffusion of ^*n*^hexane drove the reaction forward. Solvated dark blue/purple crystals of [**1E**][B(C_6_F_5_)_4_] (E = Se, Te) were isolated within 2 d by diffusion-controlled crystallisation from CH_2_Cl_2_/^*n*^hexane in yields of 30%, respectively. For [**1S**][B(C_6_F_5_)_4_], a dark blue solid was obtained in 16% yield by leaving a layered solution at −30 °C for 1 d. Low yields were attributed to the formation of side products, such as M^S^FluindPhTeF_2_, incomplete precipitation due to the high solubility of the products, and limited stability in solution (E = S). Arduous efforts to grow crystals suitable for single crystal X-ray diffraction (XRD) from solutions of [**1S**][B(C_6_F_5_)_4_] by variation of temperature, slow diffusion of different alkane solvents or evaporation were unsuccessful. In an effort to avoid XeF_2_ as an oxidant, we reacted **1Te** with the nitrosyl salt NO[SbF_6_] in the presence of K[B(C_6_F_5_)_4_] in CH_2_Cl_2_ or propionitrile at room temperature, which produced [**1Te**][B(C_6_F_5_)_4_] in 56% yield. In the absence of K[B(C_6_F_5_)_4_], [**1Te**][SbF_6_] was obtained as dark purple crystals (see Supplementary Fig. S66 for molecular structure) in 42% yield. The same procedure applied to **1S** and **1Se** failed to give the desired oxidation products, due to the limited oxidising power of NO[SbF_6_]^50^.

Considering the second electrochemical oxidation event for **1Te**, we attempted the two-electron oxidation with one equivalent of XeF_2_ at −40 °C in CH_2_Cl_2_, providing the diaryltellurium difluoride M^S^FluindPhTeF_2_, which was isolated as a colourless crystalline solid in 98% yield and fully characterised (see Supplementary Note 8 and Fig. S67). Attempts to abstract both fluoride ions from M^S^FluindPhTeF_2_ using K[B(C_6_F_5_)_4_] resulted in the intermediary adduct [M^S^FluindPhTeF_2_]_2_·K[B(C_6_F_5_)], which was characterised by single crystal XRD (Supplementary Fig. S68). Isolation of a pure bulk material of said adduct from the reaction mixture was met with failure and slow formation of [**1Te**][B(C_6_F_5_)_4_] was indicated by a colour change to pale violet. Similarly, the reaction of M^S^FluindPhTeF_2_ with solid [Et_3_Si(μ-H)SiEt_3_][B(C_6_F_5_)_4_] resulted in a dark purple solution from which crystals of [**1Te**][B(C_6_F_5_)_4_] were isolated. From the reaction mixture of M^S^FluindPhTeF_2_ with Cs[B_12_Cl_12_], colourless crystals of the hydroxytelluronium salt [M^S^FluindPhTeOH]_2_[B_12_Cl_12_] deposited, which were characterised by XRD (Supplementary Fig. S69). The latter presumably formed by hydrolysis with traces of water or the reaction with the borosilicate glass. The diarylchalcogenide radical cations [**1E**][B(C_6_F_5_)_4_] (E = S, Se, Te) showed no sign of decomposition after several months in the solid-state when stored under argon (E = S) or air (E = Se, Te). The stability in solution was monitored by keeping 1 mM solutions in dry CH_2_Cl_2_, either in the dark or exposed to ambient light, for several days and periodically measuring ultraviolet-visible-near-infrared (UV-Vis-NIR) absorption spectra (see Supplementary Tables S28–S30). For [**1S**][B(C_6_F_5_)_4_], the half-life was 6 d in the dark and 1 d exposed to ambient light. The heavier analogues [**1E**][B(C_6_F_5_)_4_] (E = Se, Te) remained virtually unchanged for at least 2 weeks in the dark, while the half-lives were greater than 20 d when exposed to ambient light.

The molecular structures of the radical cations [**1Se**]^•+^ and [**1Te**]^•+^, established by single crystal X-ray diffraction (Fig. 2C), show clear separation from their [B(C_6_F_5_)_4_]^–^ counter-ions, which is in marked contrast to the structure of [**1Te**][SbF_6_] revealing a significant Te‧‧‧F contact (3.105(1) Å) between the [**1Te**]^•+^ radical cation and the [SbF_6_]^–^ anion (Supplementary Fig. S66). The single-electron oxidation is manifested only by marginal structural changes. The Se–C_Ph_ and Te–C_Ph_ bond lengths decrease from 1.9147(11) to 1.866(2) Å and from 2.117(2) to 2.0795(13) Å, respectively, while the Se–C_Fluind_ and Te–C_Fluind_ bond lengths of 1.9212(10)/1.9203(19) Å and 2.1294(18)/2.1196(13) Å remain (almost) unchanged. The C–Se–C and C–Te–C bond angles only slightly increase from 100.44(5)° to 102.65(9)° and from 99.09(8)° to 103.11(5)°, respectively. These values are in good agreement with the density functional theory (DFT) optimised molecular structures of [**1Se**]^•+^ and [**1Te**]^•+^. While no experimental structure of [**1S**]^•+^ was obtained, the computationally optimised geometry of [**1S**]^•+^ confirms the trend observed for the heavier analogues in regard of shorter S–C bonds as well as a slightly larger C–S–C angle compared to the neutral species (Table 1).

**Table. 1:** Selected bond lengths (Å) and angles (°) of chalcogenides **1E** (E = S, Se, Te) and the respective radical cations

Parameter	1S	/	[1S]^•+^
S1–C10	1.789(3)		*1.7620*
S1–C70	1.783(3)		*1.7181*
C10–S1–C70	103.49(14)		*105.77*
	**1Se**	[**1Se**][B(C_6_F_5_)_4_]	[**1Se**]^•+^
Se1–C10	1.9212(10)	1.9203(19)	*1.9098*
Se1–C70	1.9147(11)	1.866(2)	*1.8687*
C10–Se1–C70	100.44(5)	102.65(9)	*102.99*
	**1Te**	[**1Te**][B(C_6_F_5_)_4_]	[**1Te**]^•+^
Te1–C10	2.1294(18)	2.1196(13)	*2.1135*
Te1–C70	2.117(2)	2.0795(13)	*2.0789*
C10–Te1–C70	99.09(8)	103.11(5)	*100.19*

DFT calculated values are given in italics.

Unlike their neutral parents, the diarylchalcogenide radical cations are NMR silent, but EPR active. The continuous-wave X-band (~9.6 GHz) and Q-band (~34 GHz) EPR spectra of [**1E**][B(C_6_F_5_)_4_] (E = S, Se, Te) were collected as frozen solutions in CH_2_Cl_2_/THF (1:1) under non-saturating microwave conditions and at decreasing temperatures for the heavier species, reflecting the faster electronic relaxation of the heavier chalcogenide centres (Fig. 3C). The X-band EPR spectrum of [**1S**][B(C_6_F_5_)_4_] is consistent with the radical nature of this species, showing a sharp signal in the **g** ≈ 2 area, with a minor **g**-anisotropy, yielding an approximately axial spectrum. Due to the increased resolution of the **g**-tensor at higher microwave frequencies, the Q-band EPR spectrum of [**1S**][B(C_6_F_5_)_4_] exhibits a slightly rhombic **g**-tensor as well as electron-nuclear hyperfine interaction, which is best simulated with a pair of equivalent hydrogen nuclei (Fig. 3C, Supplementary Table S73). The EPR spectra of [**1Se**][B(C_6_F_5_)_4_] and [**1Te**][B(C_6_F_5_)_4_] are much broader due in part to the increased spin-orbit contribution to the shifts of the **g**-values for the heavier chalcogen radical centres, supporting that the unpaired electron density is mostly localised on the chalcogen atom, in agreement with previously characterised dichalcogenide radical species^51^. The spectra are also more complex due to increased rhombicity of the **g**-tensor and the resolved electron-nuclear hyperfine interactions (**A**) for the NMR-active isotopes of selenium and tellurium (^77^Se, *I* = 1/2, 7.63%; ^125^Te, *I* = 1/2, 7.07%)^52^. Simulation of the data enabled the extraction of the **g** and **A** tensors (Table 2), where the relative signs of the components in the **A** tensor were determined using the previously reported procedure^53,54^. Extracting values of the isotropic hyperfine coupling (*a*_*iso*_) and dipolar coupling tensors (**T**) from the **A** tensor (*a*_*iso*_ = (*A*_1_ + *A*_2_ + *A*_3_)/3; **T** = **A**–*a*_*iso*_, where **T** takes the approximate form **T** = [−*t*, −*t*, 2*t*]) enables the estimation of unpaired electron density in *s* and *p* orbitals of chalcogen atoms in [**1Se**][B(C_6_F_5_)_4_] and [**1Te**][B(C_6_F_5_)_4_], as previously described^53,54^. Obtained values of *a*_*iso*_(Se) = 184 MHz and *t*(Se) = 294 MHz provide an estimate of unpaired electron population in *s* orbitals *ρ*(*s*,Se) = 0.009 and in *p* orbitals *ρ*(*p*,Se) = 0.598 of selenium. This indicates that the majority of the unpaired spin population is localised in the *p* orbitals of the selenium atom, which is in a good agreement with DFT calculated Mulliken spin density. Similar results for [**1Te**][B(C_6_F_5_)_4_] are obtained, with minimal *s* orbital unpaired electron population and a large *p* orbital population. The localisation of the unpaired electron to a chalcogen *p* orbital is in good agreement with calculated Mulliken spin density calculations (Table 2). The EPR spectra of [**1S**][B(C_6_F_5_)_4_] contain no sulphur hyperfine interaction as the only NMR-active nucleus ^33^S (*I* = 3/2) has a natural abundance of only 0.76%^52^. However, the small but appreciable **g**-shift observed for [**1S**][B(C_6_F_5_)_4_] indicates spin-orbit coupling from the S atom. DFT predicts a similar **g**-tensor to the experiment, and the visualisation of the spin density plot for [**1S**][B(C_6_F_5_)_4_] (Fig. 2B) reveals a similar picture compared to [**1Se**][B(C_6_F_5_)_4_] and [**1Te**][B(C_6_F_5_)_4_], supporting that [**1S**][B(C_6_F_5_)_4_] can also be described as a chalcogenide *p* radical centre. However, the Mulliken spin populations increase in the order [**1S**]^•+^ < [**1Se**]^•+^ < [**1Te**]^•+^, with the adjacent phenyl rings showing spin populations in the reverse order, indicating more effective orbital overlap between the *p*-Orbital and the π-system in case of [**1S**]^•+^.

**Table. 2:** Comparison of experimental (obtained from simulation of Q-band spectra) and DFT calculated EPR parameters and unpaired spin densities on chalcogens

	g	A (MHz)	*ρ*(*s*)^a^	*ρ*(*p*)^b^
[**1S**][B(C_6_F_5_)_4_]	[2.00693, 2.00476, 2.00198] *[2.01116, 2.00682, 2.00241]*	–	– *0.025*	– *0.407*
[**1Se**][B(C_6_F_5_)_4_]	[2.05450, 2.02713, 1.99812] *[2.05497, 2.02780, 2.00273]*	[−90, −130, 772] *[*−*169*, −*217, 609]*	0.009 *0.026*	0.598 *0.534*
[**1Te**][B(C_6_F_5_)_4_]	[2.14351, 2.04322, 1.97201] *[2.14188, 2.05409, 2.00373]*	[690, 950, −2185] *[564, 862*, −*1658]*	0.003 *0.043*	0.955 *0.702*

DFT calculated values are given in italics.

^a^*ρ*(*s*) = *a*_iso_/*a*_0_ where *a*_0_ is isotropic hyperfine parameter, *a*_0_(Se) = 20120 MHz, *a*_0_(Te) = −55590 MHz. *a*_0_ values are obtained from ref. ^52^.

^b^*ρ*(*p*) = *t*/*b*_0_ where *b*_0_ is uniaxial hyperfine parameter, *b*_0_(Se) = 491.6 MHz, *b*_*0*_(Te) = −1048.7 MHz. *b*_0_ values are obtained from ref. ^52^.

The optical absorption spectra of [**1E**][B(C_6_F_5_)_4_] exhibit intense, broad absorption bands in the near IR region (E = S, 1468 nm; E = Se, 1155 nm; E = Te, 837 nm), which shift toward higher energies with increasing mass of the chalcogen (Fig. 3B). Beside these bands, a series of bands can be observed at higher energies in the visible red (E = S, 689 nm; E = Se, 653 nm), visible green (E = S, 500 nm; E = Se, 507 nm; E = Te, 552 nm) and near UV (E = S, 307 nm; E = Se, 332 nm, E = Te, 340 nm) areas, resulting in deep turquoise (E = S), blue (E = Se) and purple (E = Te) colours of compounds. Closely similar absorption bands could be obtained from spectroelectrochemical studies of **1E** (E = S, Se, Te; see Supplementary Figs. S51–S62). Owing to the lability of [**1S**][B(C_6_F_5_)_4_] in solution, UV-Vis-NIR measurements showed an absorption band at 464 / 500 nm, which was attributed to an unidentified decomposition product. This impurity shows a similar solubility to [**1S**][B(C_6_F_5_)], but by extracting with DCM/heptane (1:3) mixture and immediately measuring UV-Vis-NIR, a purified sample can be obtained. Formation of the side product can then be followed. Attempts to isolate this impurity were not successful, since it is a transient species as well. The ^19^F NMR spectrum of [**1S**][B(C_6_F_5_)_4_] shows minor decomposition of the [B(C_6_F_5_)_4_]^−^ counter-ion, and LIFDI HRMS measurements hint at the presence of [**1S**+F]^+^, therefore a reaction with the counter-ion is a plausible explanation (see Supplementary Figs. S13–S17).

The UV-Vis-NIR spectra are in good agreement with TDDFT calculations (Supplementary Fig. S32), which also show that the near IR feature in the spectrum of [**1Te**][B(C_6_F_5_)_4_] (experimental: 837 nm, calculated: 821 nm) shifts toward lower energies for [**1Se**][B(C_6_F_5_)_4_] (experimental: 1155 nm, calculated: 1088 nm) and [**1S**][B(C_6_F_5_)_4_] (experimental: 1468 nm, calculated: 1302 nm). Analysis of the natural transition orbitals and transition difference densities showed that this low energy transition, in all studied compounds, originates from electron excitation from the π system of both fluorene parts of the M^S^Fluind ligand (β HOMO orbital), parallel to the C_Ph_–E–C_Fluind_ (E = S, Se, Te) plane, to the *p* orbital on the chalcogen atom (β LUMO orbital). In fact, the approximately first ten transitions with wavelengths >360 nm of the TDDFT of all three complexes stem from β valence → β LUMO transitions. No transitions within the α channel of the spin-unrestricted MO description are calculated until energies greater than 28,000 cm^–1^ ( < 360 nm) (Supplementary Tables S36–S38). This corresponds to the high absorbance at lower wavelength from the various charge-transfer features (Fig. 3B). Across the series, the HOMO(β)-LUMO(β) and HOMO-SOMO gaps increase with increased chalcogen mass (Supplementary Fig. S34).

The computationally derived electron density-based bond descriptors AIM^55^ and IGMH^56,57^ were analysed for all radical cations to unravel any weak intermolecular interactions of the chalcogen towards the flanking fluorenyl groups. The AIM analyses of [**1S**]^•+^ reveal bond paths towards a C-atom of each of the fluorenyl groups (Fig. 2B). However, key parameters such as the electron densities at the bond critical points (0.13–0.14 eÅ^–3^) as well as the Laplacians (0.9–1.1 eÅ^–5^) indicate only weak interactions. This is further corroborated by the inspection of the IGMH iso-surfaces showing predominantly green areas reminiscent of weak dispersive interactions between the EPh (E = S, Se, Te) fragment and the flanking groups (Supplementary Fig. S40).

In summary, deeply coloured, kinetically-stabilised chalcogenide radical cations, [M^S^FluindPhE]^•+^ (E = S, Se, Te), have been isolated, in which a bulky M^S^Fluind substituent prevents dimerisation and fast decomposition (M^S^Fluind = dispiro[fluorene-9,3′-(1′,1′,7′,7′-tetramethyl-*s*-hydrindacene-4′-yl)-5′,9″-fluorene]). In the series **1E** (E = Te < Se < S), higher oxidation potentials were found for the lighter homologues. The heavier homologues formed more stable radical cations **[1E]**^•+^, as underlined by larger HOMO-SOMO gaps and longer half-lives. EPR measurements of **[1E]**^•+^ showed a majority of the unpaired spin density to be localised in the *p*-orbitals of the chalcogen centres, the population of which increases with the mass of the chalcogen.

## Methods

### General methods

Unless otherwise stated, all manipulations were performed under an inert argon atmosphere using anhydrous solvents at room temperature. Reagents used in this work were obtained commercially and were used as received. M^S^FluindLi(THF)_2_^31^, K[B(C_6_F_5_)]_4_^58^ were prepared according to published procedures. NMR spectra were recorded at room temperature on Bruker Avance Neo 600 MHz spectrometers. ^1^H (600 MHz), ^13^C{^1^H} (151 MHz), ^11^B{^1^H} (193 MHz), ^19^F (565 MHz), ^77^Se (114 MHz) and ^125^Te (189 MHz) NMR spectra are reported on the *δ* scale (ppm) and are referenced against SiMe_4_. ESI HRMS spectra were measured on a Bruker Impact II spectrometer using CH_2_Cl_2_/MeCN solutions (direct injection). LIFDI HRMS spectra were acquired on a Thermo Fisher Orbitrap Exploris 240 upgraded with a LIFDI source (Linden CMS) using solutions in CH_2_Cl_2_. UV-Vis-NIR spectra were measured on an Agilent Cary 6000i spectrophotometer in a range of 300–1800 nm, in a 2 mm quartz cuvette. DFT calculations were performed for compounds [**1E**][B(C_6_F_5_)_4_] (E = S, Se, Te) using ORCA version 6.0.0^59,60^. EPR measurements were performed on frozen solutions of [**1E**][B(C_6_F_5_)_4_] (E = S, Se, Te) in CH_2_Cl_2_/THF mixture (1:1). Spectra were collected on a Bruker Elexsys E500 spectrometer, equipped with the Bruker dual-mode cavity (ER4116DM), at 7.4, 22 or 60 K for X-band measurements and on a Bruker Elexsys E580 spectrometer equipped with a home-built up/down Q-band conversion accessory, at 25, 35, 70 K. Cyclic voltammetry studies were performed in a V-tube electrochemical cell at room temperature using an Autolab PGSTAT 101 (Metrohm) Electrochemical Workstation with a three-electrode configuration. The potentials are reported against the ferrocene/ferrocenium redox couple (FcH/FcH^+^). Spectroelectrochemical studies were performed using a Microcell HC cell stand from rhd instruments GmbH & Co.KG combined with the TSC spectro measuring cell. An Autolab PGSTAT 101 Metrohm potentiostat was used for bulk electrolysis. A three-electrode configuration was used. Single crystal X-Ray diffraction intensity data was collected on a Bruker Venture D8 diffractometer at 100 K with graphite-monochromatic Mo-Kα (0.7107 Å) radiation. More detailed information on the methods used is given in Supplementary Methods 1.

### General synthesis of M^S^FluindPhE (1E, E = S, Se, Te)

A solid mixture of M^S^FluindLi(THF)_2_ (1.00 g, 1.50 mmol) and diphenyl dichalcogenide (Ph_2_E_2_, 1.60 mmol) was suspended in toluene (20–40 mL) in a Schlenk tube and stirred for 3–7 d. After aqueous workup and extraction with large amounts of CH_2_Cl_2_ (250–1000 mL), the organic phase was separated, filtered through a plug of Celite®-535 and the solvent was removed by rotary evaporation. The solid was washed with diethyl ether (3 × 7 –10 mL) and then repeatedly (1–3 times) recrystallised from boiling toluene, affording crystals suitable for single crystal XRD. After decanting and drying of the colourless or yellow solid under reduced pressure (80 °C, 1 d, 10^−3^ mbar), the title compounds were obtained in yields of 53–62%. ^**77**^**Se NMR** (**1Se**, 114 MHz, CDCl_3_): *δ* = 314.2 ppm. ^**125**^**Te NMR** (**1Te**, 189 MHz, CDCl_3_): *δ* = 506.7 ppm. Detailed procedures and analytical data are included in Supplementary Notes 1–3.

### Synthesis and characterisation of [1S][B(C_6_F_5_)_4_]

A solid mixture of **1S** (182 mg, ca. 98% purity, 286 µmol) and K[B(C_6_F_5_)_4_] (206 mg, 287 µmol) was placed in a Schlenk tube and suspended in CH_2_Cl_2_ (7 mL) while stirring. The vessel was cooled to −60 °C using a cooling bath and XeF_2_ (24.0 mg, 143 µmol) was added. The cooling bath was removed, and the reaction mixture was stirred for 1.5 h under exclusion of light. The solution was filtered using a syringe filter, then ^*n*^heptane (10 mL) was layered on top of the solution. After storing at −30 °C for 1 d (in darkness), the solution was decanted off. The remaining amorphous dark blue solid was dried under reduced pressure (10^−3^ mbar), affording [**1S**][B(C_6_F_5_)_4_]·(60.0 mg, 46.1 µmol, 16%). The cation is ^1^H and ^13^C{^1^H} NMR silent in CD_2_Cl_2_. ^**11**^**B{**^**1**^**H} NMR** (193 MHz, CD_2_Cl_2_): *δ* = −16.65 ppm. ^**19**^**F NMR** (565 MHz, CD_2_Cl_2_): *δ* = −133.14 (s, br), −163.61 (t, *J* = 20.3 Hz), −167.48 (s, br) ppm. **UV-Vis-NIR** (CH_2_Cl_2_, 300–1800 nm): *λ* (*ε*) = 307 (18800), 464* (3600), 500* (3300), 689 (8400), 1468 (3200) nm (L mol^−1^ cm^−1^) (*attributed to an unidentified impurity). **LIFDI HRMS** (m/z): [M−Ph+H]^+^ calculated for C_40_H_33_S: 545.22975, found: 545.2298; [M]˙^+^ calculated for C_46_H_38_S: 622.26887, found: 622.2693; [M+CH]^+^ calculated for C_47_H_39_S: 635.27670, found: 635.2772; [M+F]^+^ calculated for C_46_H_38_FS: 641.26728, found: 641.2680. *Melting point*: 266–268 °C. NMR, UV-Vis-NIR and HRMS spectra are included in Supplementary Note 4.

### Synthesis and characterisation of [1Se][B(C_6_F_5_)_4_]

A solid mixture of **1Se** (30.0 mg, 44.8 µmol) and K[B(C_6_F_5_)_4_] (32.2 mg, 44.8 µmol) was placed in a Schlenk tube and suspended in CH_2_Cl_2_ (3 mL) while stirring. The vessel was cooled to −60 °C using a cooling bath and XeF_2_ (3.9 mg, 23 µmol) was added. The mixture was left to slowly warm up to room temperature while stirring for 16 h. The solution was filtered using a syringe filter and ^*n*^hexane (5 mL) was layered on top of the solution. After 2 d the colourless precipitate was removed by decanting and ^*n*^hexane (5 mL) was layered on top of the solution. After 4 d the solution was decanted off and the remaining dark blue solid was dried under reduced pressure (10^−3^ mbar), affording [**1Se**][B(C_6_F_5_)_4_]·2.5 CH_2_Cl_2_ (21.0 mg, 13.5 µmol, 30%). Crystals suitable for single crystal XRD were obtained by slow diffusion of ^*n*^hexane into a solution of [**1Se**][B(C_6_F_5_)_4_]in CH_2_Cl_2_. The Cation is ^1^H, ^13^C{^1^H} and ^77^Se NMR silent in CD_2_Cl_2_. ^**11**^**B{**^**1**^**H} NMR** (193 MHz, CD_2_Cl_2_): *δ* = −16.65 (s) ppm. ^**19**^**F NMR** (565 MHz, CD_2_Cl_2_): *δ* = −132.99 (s, br, 8F), −163.51 (t, *J* = 19.6 Hz, 4F), −167.31 (s, br, 8F) ppm. **UV-Vis-NIR** (CH_2_Cl_2_, 300–1800 nm): *λ* (*ε*) = 307 (13700), 332 (6000), 507 (2700), 653 (3900), 1155 (4800) nm (L mol^−1^ cm^−1^). **ESI HRMS** (m/z): [M]^•+^ calculated for C_46_H_38_Se: 670.21379, found: 670.21346, [M+OH]^+^ calculated for C_46_H_39_OSe: 687.21654, found: 687.21600. *Melting point*: 198–200 °C. NMR spectra are included in Supplementary Note 5.

### Synthesis and characterisation of [1Te][B(C_6_F_5_)_4_]

*Method A*: A solid mixture of **1Te** (105 mg, 0.146 µmol) and K[B(C_6_F_5_)_4_] (107 mg, 0.149 µmol) was placed in a Schlenk tube and suspended in CH_2_Cl_2_ (7 mL) while stirring. The vessel was cooled to −60 °C using a cooling bath and XeF_2_ (12.3 mg, 72.7 µmol) was added. The cooling bath was removed, and the mixture was stirred for 2 h. ^*n*^Hexane (1 mL) was added and the mixture was stirred for 1 h. The solution was filtered using a syringe filter and ^*n*^hexane (7 mL) was layered on top of the solution. After 1 d the solution was decanted off and the remaining dark purple solid was dried under reduced pressure (10^−3^ mbar) affording [**1Te**][B(C_6_F_5_)_4_]·CH_2_Cl_2_ (65.0 mg, 43.9 µmol, 30%).

*Method B*: A solid mixture of **1Te** (51.0 mg, 71.0 µmol), K[B(C_6_F_5_)_4_] (51.0 mg, 71.0 mmol) and NO[SbF_6_] (19.0 mg, 71.5 mmol) was placed in a Schlenk tube, suspended in CH_2_Cl_2_ (4 mL) and stirred for 16 h at room temperature. ^*n*^Hexane (1 mL) was added and the mixture was stirred for 1 h. The solution was filtered using a syringe filter and ^*n*^hexane (4 mL) was layered on top of the solution. After 16 h the solution was decanted off and the remaining dark purple solid, which contained crystals suitable for single crystal XRD, was dried under reduced pressure (10^−3^ mbar) affording [**1Te**][B(C_6_F_5_)_4_]·CH_2_Cl_2_ (58.9 mg, 39.7 µmol, 56%). The cation is ^1^H, ^13^C{^1^H} and ^125^Te NMR silent in CD_2_Cl_2_. ^**11**^**B{**^**1**^**H} NMR** (193 MHz, CD_2_Cl_2_): *δ* = −16.65 (s, br) ppm. ^**19**^**F NMR** (565 MHz, CD_2_Cl_2_): *δ* = −132.97 (s, br, 8F), −163.41 (t, *J* = 19.6 Hz, 4F), −167.21 (s, br, 8F) ppm. **UV-Vis-NIR** (CH_2_Cl_2_, 300–1800 nm): *λ* (*ε*) = 307 (12100), 340 (4000), 552 (4000), 837 (3200) nm (L mol^−1^ cm^−1^). **ESI HRMS** (m/z): [M]^•+^ calculated for C_46_H_38_Te: 720.20303, found: 720.20196, [M+OH]^+^ calculated for C_46_H_39_OTe: 737.20577, found: 737.20458. *Melting point*: 246–248 °C. NMR spectra are included in Supplementary Note 6.

## Supplementary information

Supplementary Information

Description of Additional Supplementary Files

Supplementary Data 1

Supplementary Data 2

Supplementary Data 3

Supplementary Data 4

## Data Availability

Experimental details on the synthesis and characterisation of all isolated compounds can be found in the Supplementary Information. This includes detailed general methods (Supplementary Methods 1); synthetic procedures of **1S**, **1Se**, **1Te**, [**1Te**][SbF_6_], M^S^FluindPhTeF_2_ (Supplementary Notes 1, 2, 3, 4, 7 and 8); NMR spectra of **1S**, **1Se**, **1Te** (Supplementary Notes 1–3), M^S^FluindPhTeF_2_ (Supplementary Note 8); UV-Vis (‑NIR) spectra of [**1S**][B(C_6_F_5_)_4_], **1Te** and [**1Te**][SbF_6_] (Supplementary Figs. S12, S17 and S22); a LIFDI HRMS spectrum of [**1S**][B(C_6_F_5_)_4_] (Supplementary Fig. S13); computational methods and results (Supplementary Note 10); cyclic voltammograms of **1S**, **1Se**, **1Te** (Supplementary Note 11); spectroelectrochemical measurements of **1S**, **1Se**, **1Te** (Supplementary Note 12); molecular structures of **1S**, **1Se**, **1Te**, [**1Te**][SbF_6_], M^S^FluindPhTeF_2_, [M^S^FluindPhTeF_2_]·K[B(C_6_F_5_)_4_] and [M^S^FluindPhTeOH][B_12_Cl_12_]_0.5_, crystal data and structure refinements of all structurally characterised compounds (Supplementary Note 13). Source data for cyclic voltammograms are provided in Supplementary Data 1. Source data for UV-Vis-NIR spectra are provided in Supplementary Data 2. Cartesian coordinates of the optimised DFT models are provided in Supplementary Data 3. Crystallographic Information Files of the reported molecular structures are given in Supplementary Data 4. Crystallographic data for the structural analyses have been deposited with the Cambridge Crystallographic Data Centre (Deposition Numbers: 2419127–2419135).
