# Supplementary material for: Isolation of kinetically-stabilised diarylchalcogenide radical cations
Source: Commun Chem. 2025 Aug 9;8:239. doi: 10.1038/s42004-025-01613-z (PMC12335450; doi:10.1038/s42004-025-01613-z)
Supplement: Supplementary file 1 — Supplementary Information [file 42004_2025_1613_MOESM1_ESM.pdf]

Table of Contents

Supplementary Methods 1. General Methods 2

Supplementary Note 1. Synthesis and Characterization of M^SFluindPhS (1S) 3

Supplementary Note 2. Synthesis and Characterization of M^SFluindPhSe (1Se) 6

Supplementary Note 3. Synthesis and Characterization of M^SFluindPhTe (1Te) 9

Supplementary Note 4. Characterization of [1S][$B(C_6F_5)_4$] 12

Supplementary Note 5. Characterisation of [1Se][$B(C_6F_5)_4$] 14

Supplementary Note 6. Characterisation of [1Te][$B(C_6F_5)_4$] 14

Supplementary Note 7. Synthesis and Characterisation of [1Te][SbF_6] 15

Supplementary Note 8. Synthesis and Characterisation of M^SFluindPhTeF_2 16

Supplementary Note 9. Stability of [1E][$B(C_6F_5)_4$] (E = S, Se, Te) in Solution 19

Supplementary Note 10. Computational Results 20

Supplementary Note 11. Cyclic Voltammetry 26

Supplementary Note 12. Spectroelectrochemical Studies 31

Supplementary Note 13. X-Ray Diffraction Studies 38

Supplementary Note 14. Electron Paramagnetic Resonance 45

Supplementary References 46

Supplementary Methods 1. General Methods

Unless otherwise stated, all reactions, manipulations, work-up and purifications were performed under inert argon atmosphere using anhydrous solvents at room temperature. Reagents used in this work were obtained commercially and were used as received. M^SFluindLi(THF)$_2$,[1] K[B(C_6F_5)]$_4$[2] were prepared according to published procedures. Anhydrous solvents (dichloromethane, ^{n}hexane, toluene) were collected from an SPS800 mBraun solvent purification system and stored over 4 Å molecular sieves. Deuterated solvents were degassed and dried over 4 Å molecular sieves under argon. **NMR** spectra were recorded at room temperature on Bruker Avance Neo 600 MHz spectrometers. ^{1}H (600 MHz), ^{13}C{^{1}H} (151 MHz), ^{11}B{^{1}H} (193 MHz), ^{19}F (565 MHz), ^{77}Se (114 MHz) and ^{125}Te (189 MHz) NMR spectra are reported on the δ scale (ppm) and are referenced against $SiMe_4$. ^{1}H and ^{13}C{^{1}H} NMR chemical shifts are reported relative to the residual peak of the solvent ($CHCl_3$: 7.26 ppm, for chloroform-d_1; $CDHCl_2$: 5.32 ppm, for dichloromethane-d_2) in the ^{1}H NMR spectra, and to the peak of the deuterated solvent ($CDCl_3$: 77.16 ppm; CD_2Cl_2: 53.84 ppm) in the ^{13}C{^{1}H} NMR spectra.[3] The assignment of the ^{1}H and ^{13}C{^{1}H} resonance signals was made in accordance with the HSQC and HMBC spectra. The **ESI HRMS** spectra were measured on a Bruker Impact II spectrometer. Dichloromethane/acetonitrile solutions ($c = 1\cdot10^{-5}$ mol·L^{-1}) were injected directly into the spectrometer at a flow rate of 3 µL·min^{-1}. Nitrogen was used both as a drying gas and for nebulization with flow rates of approximately 5 L·min^{-1} and a pressure of 5 psi. Pressure in the mass analyser region was usually about $1\cdot10^{-5}$ mbar. Spectra were collected for 1 min and averaged. The nozzle-skimmer voltage was adjusted individually for each measurement. **LIFDI HRMS** spectra were acquired on a Thermo Fisher Orbitrap Exploris 240 upgraded with a LIFDI source (Linden CMS). The compounds were dissolved in dry CH_2Cl_2 (ca. 0.5 mg·mL^{-1}) in septum capped vials. Approximately 100 nL of solution was directly infused in the LIFDI source through a fused silica capillary of 0.75 µm inner diameter. The extraction voltage was −7 kV at an ion energy of +10 eV. A temperature program was applied by means of an emitter heating current ramp of 60 mA min^{-1} for two minutes. **UV-Vis-NIR** spectra were measured on a VWR UV-1600PC spectrophotometer in a range of 300—1100 nm, in a 10 mm quartz cuvette OR on an Agilent Cary 6000i spectrophotometer in a range of 300—1800 nm, in a 2 mm quartz cuvette. **EPR** measurements were performed on frozen solutions of [1E][B(C_6F_5)$_4$] (E = S, Se, Te) in CH_2Cl_2/THF mixture (1:1). Samples were prepared in a glovebox and filled in a custom 4 mm (for X band) and 1.6 mm (for Q band) quartz EPR tube. Continuous wave (CW) X-band spectra were collected on a Bruker Elexsys E500 spectrometer, equipped with the Bruker dual-mode cavity (ER4116DM), at 7.4, 22 or 60 K, with following parameters: 100 kHz field modulation frequency, 2 or 4 G modulation amplitude, 120 s scan time, 5 scans. Desired temperature was achieved with an Oxford Instruments helium flow cryostat ESR 900 and temperature controller. CW Q-band spectra were collected on a Bruker Elexsys E580 spectrometer equipped with a home-built up/down Q-band conversion accessory, at 25, 35, 70 K, with following parameters: 100 kHz field modulation frequency, 3 or 6 G modulation amplitude, 60 or 120 s scan time. Desired temperature was achieved with an Oxford Instruments helium flow cryostat C-935 and temperature controller The data was processed and analysed in Matlab R2021b and simulated using the EasySpin package (v. 6.0.5).[4]

Supplementary Note 1. Synthesis and Characterization of M^SFluindPhS (1S)

A solid mixture of M^SFluindLi(THF)$_2$ (1.00 g, 1.50 mmol) and diphenyl disulphide* (350 mg, 1.60 mmol) was suspended in toluene (40 mL) in a Schlenk-tube and stirred for 7 d. Water (10 mL) was added and the mixture was stirred for 15 min. The mixture was combined with water (100 mL) and CH_2Cl_2 (1 L). The organic phase was separated, filtered through a plug of Celite®-535 and the solvent was removed by rotary evaporation. The solid was washed with diethyl ether (3 × 10 mL) and dried under reduced pressure (10^{-3} mbar), affording the crude product (847 mg, ca. 90 % purity, ca. 81 % crude yield), which contained ca. 10 % of M^SFluindH as a side product, as determined by ^{1}H NMR. The crude product was recrystallized from boiling toluene (ca. 100 mL, not fully dissolved) with subsequent reduction of the solvent to ca. 20 mL by rotary evaporation (repeated 2—3 times). After decanting and drying of the colourless solid under reduced pressure (80 °C, 1 d, 10^{-3} mbar), the title compound was obtained in a yield of 61 % (571 mg, 917 µmol).† Colourless crystals suitable for single crystal XRD were obtained by recrystallization of **1S** from boiling toluene.

^{1}H NMR (600 MHz, $CDCl_3$): δ = 7.37 (s, 1H), 7.34 (d, J = 7.5 Hz, 4H, 12, 19, 24, 31), 6.94 (m, 4H, 13, 18, 25, 30), 6.88 (d, J = 7.5 Hz, 4H, 15, 16, 27, 28), 6.85 – 6.80 (m, 3H, 14, 17, 26, 29), 6.49 (t, J = 7.3 Hz, 1H, 51), 6.42 (t, J = 7.5 Hz, 2H, 49, 50), 5.63 (d, J = 7.7 Hz, 2H, 47, 48), 2.44 (s, 4H), 1.62 (s, 12H).

^{13}C{^{1}H} NMR (151 MHz, $CDCl_3$): δ = 156.81 (2, 6), 153.80 (8, 11, 20, 23, only observed in HMBC), 150.50 (3, 5), 140.42 (9, 10, 21, 22), 138.82 (46), 126.97 (14, 17, 26, 29), 126.43 (13, 18, 25, 30), 126.22 (49, 50), 124.98 (4), 124.00 (47, 48), 123.91 (15, 16, 27, 28), 122.20 (51), 119.11 (12, 19, 24, 31, 1, overlap), 63.40 (7, 32), 56.32 (33, 36), 43.34 (34, 35), 32.99 (37, 38, 39, 40) ppm.

ESI HRMS (m/z): $[M+Na]^+$ calculated for $C_{46}H_{38}NaS$: 645.25864, found: 645.25718; $[M+K]^+$ calculated for $C_{46}H_{38}KS$: 661.23258, found: 661.23116.

Melting point: >360 °C.

---

* The more sterically encumbered M^SFluindMesS (Mes = mesityl) could not be generated by reacting M^SFluindLi(THF)$_2$ with Mes_2S_2 or MesSCl.

†Small amounts of M^SFluindH (ca. 2—4 %) were still detected by ^{1}H NMR and could not be removed by washing, recrystallization, column chromatography or sublimation. To be noted is the very poor solubility of **1S** (ca. 1 mg/mL) in comparison to the more soluble M^SFluindH, thus **1S** may appear less pure in saturated solutions. No interference of M^SFluindH with subsequent reactions was observed.

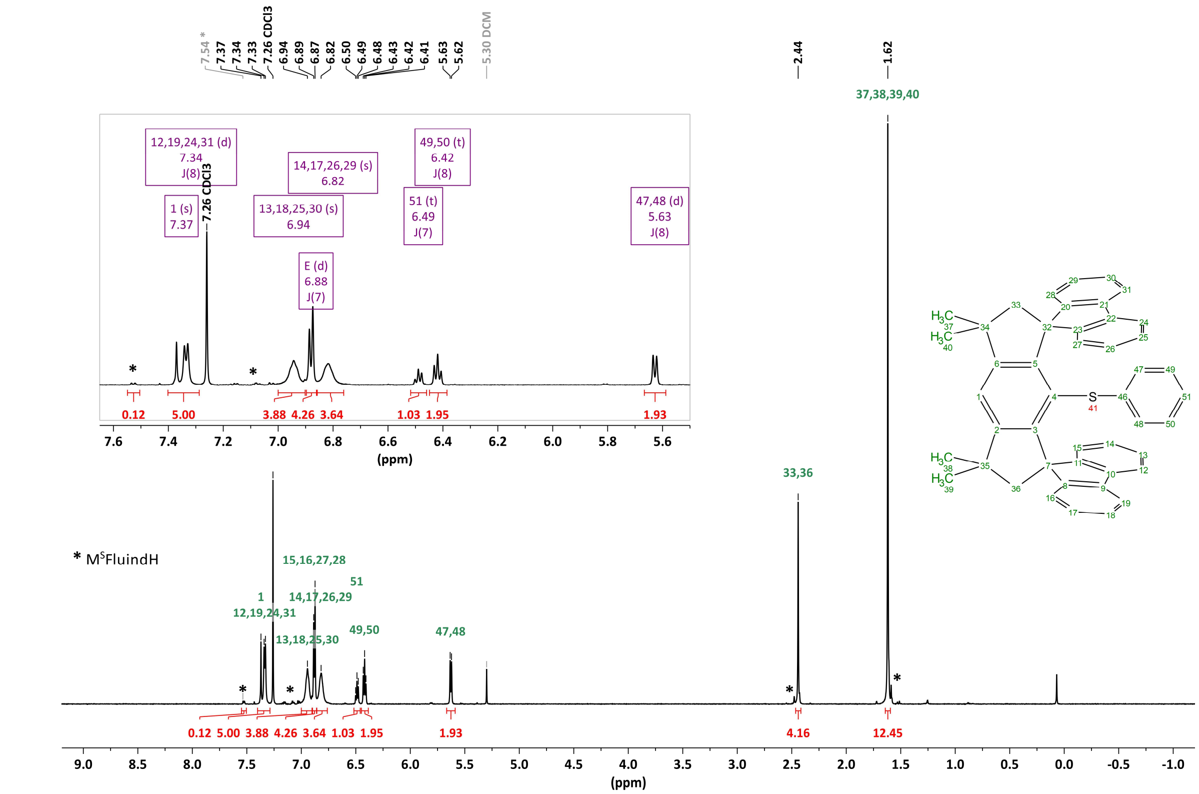

Figure S1. ^{1}H NMR (600 MHz, $CDCl_3$) spectrum of **1S**.

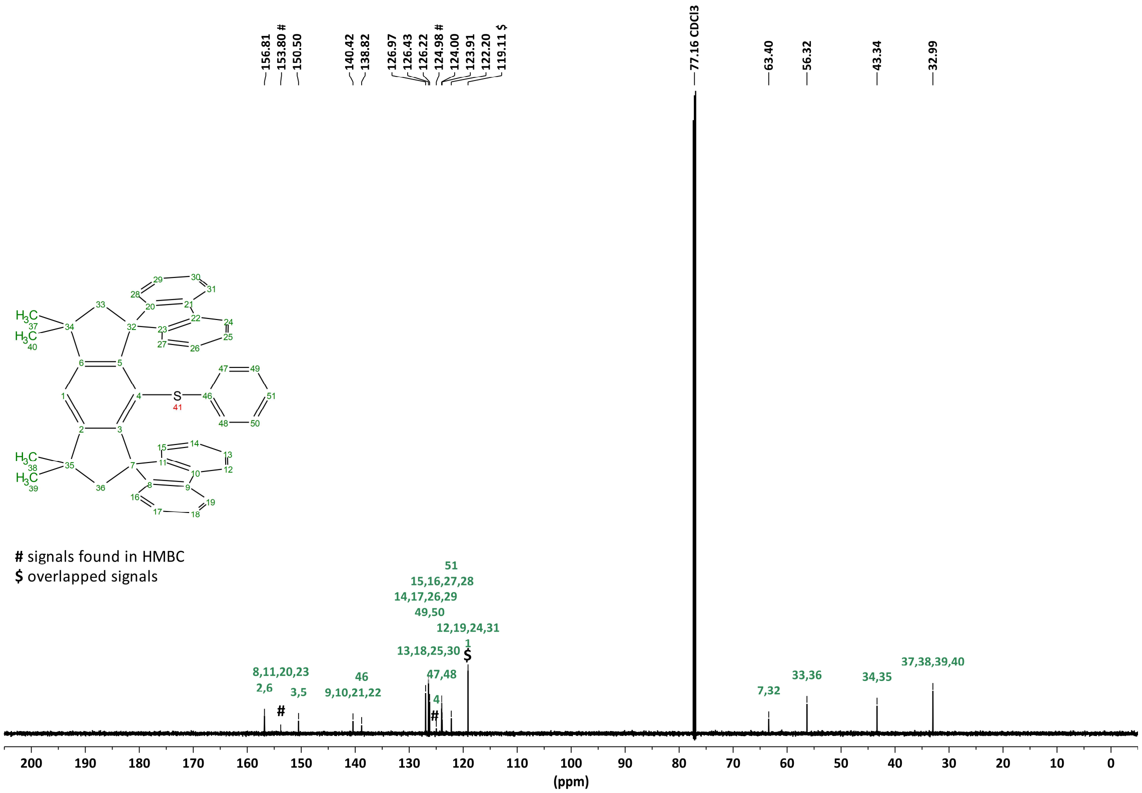

Figure S2. $^{13}C\{^{1}H\}$ NMR (151 MHz, $CDCl_3$) spectrum of **1S**.

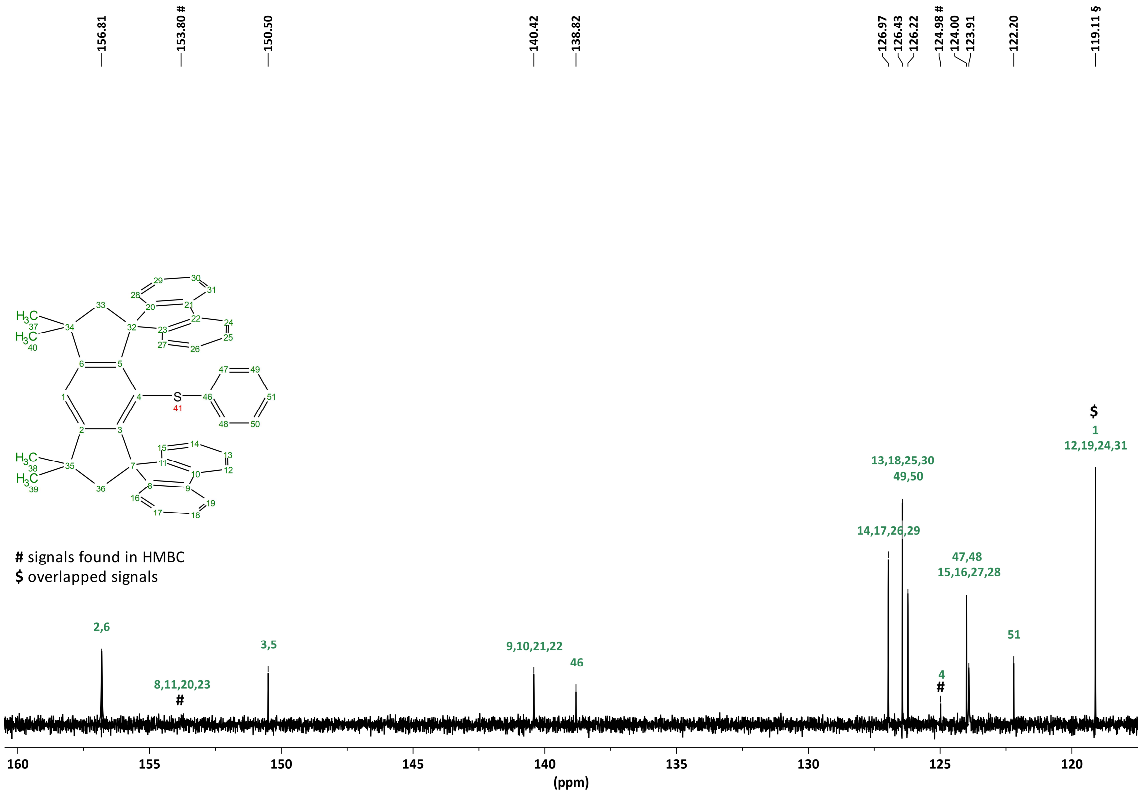

Figure S3. $^{13}C\{^{1}H\}$ NMR (151 MHz, $CDCl_3$) spectrum of **1S** (downfield area).

Supplementary Note 2. Synthesis and Characterization of M^{S}FluindPhSe (1Se)

A solid mixture of M^{S}FluindLi(THF)$_2$ (1.00 g, 1.50 mmol) and diphenyl diselenide (500 mg, 1.60 mmol) was suspended in toluene (20 mL) in a Schlenk-tube and stirred for 5 d. Water (5 mL) was added and the mixture was stirred for 15 min. The mixture was combined with water (100 mL) and CH_2Cl_2 (500 mL). The organic phase was separated, filtered through a plug of Celite®-535 and the solvent was removed by rotary evaporation. The solid was washed with diethyl ether (ca. 3 × 7 mL) and dried under reduced pressure (10^{-3} mbar), affording the crude product (819 mg, ca. 90 % purity, ca. 73 % crude yield), which contained ca. 10 % of M^{S}FluindH as a side product, as determined by ^{1}H NMR. The crude product was recrystallized from boiling toluene (repeat if necessary). After decanting and drying the solid under reduced pressure (80 °C, 1 d, 10^{-3} mbar), the title compound was obtained in a yield of 62 % (622 mg, 929 μmol). Colourless crystals suitable for single crystal XRD were obtained by recrystallization of **1Se** from boiling toluene.

^{1}H NMR (600 MHz, $CDCl_3$): δ = 7.38 (s, 1H, 1), 7.37 (d, J = 7.4 Hz, 4H, 12, 19, 24, 31), 6.97 (t, J = 7.2 Hz, 4H, 13, 18, 25, 30), 6.90 (d, J = 7.5 Hz, 4H, 15, 16, 27, 28), 6.82 (t, J = 6.7 Hz, 4H, 14, 17, 26, 29), 6.60 (t, J = 7.3 Hz, 1H, 51), 6.48 (t, J = 7.7 Hz, 2H, 49, 50), 5.81 (d, J = 7.0 Hz, 2H, 47, 48) 2.43 (s, 4H, 33, 36)), 1.61 (s, 12H, 37, 38, 39, 40) ppm.

^{13}C{^{1}H} NMR (151 MHz, $CDCl_3$): δ = 156.53 (2, 6), 154.37 (8, 11, 20, 23), 150.35 (3, 5), 140.66 (9, 10, 21, 22), 135.02 (46), 127.01, 126.70, 126.57, 126.41, 123.92 (15, 16, 27, 28), 123.16, 122.10 (4), 119.20 (12, 19, 24, 31), 119.05 (1), 64.48 (7, 32), 56.56 (33, 36), 43.16 (34, 35), 32.98 (37, 38, 39, 40) ppm.

^{77}Se NMR (115 MHz, $CDCl_3$): δ = 314.2 ppm.

ESI HRMS (m/z): $[M+Na]^+$ calculated for $C_{46}H_{38}NaSe$: 693.20356, found: 693.20120; $[M+K]^+$ calculated for $C_{46}H_{38}KSe$: 709.17748, found 709.17507.

Melting point: >360 °C.

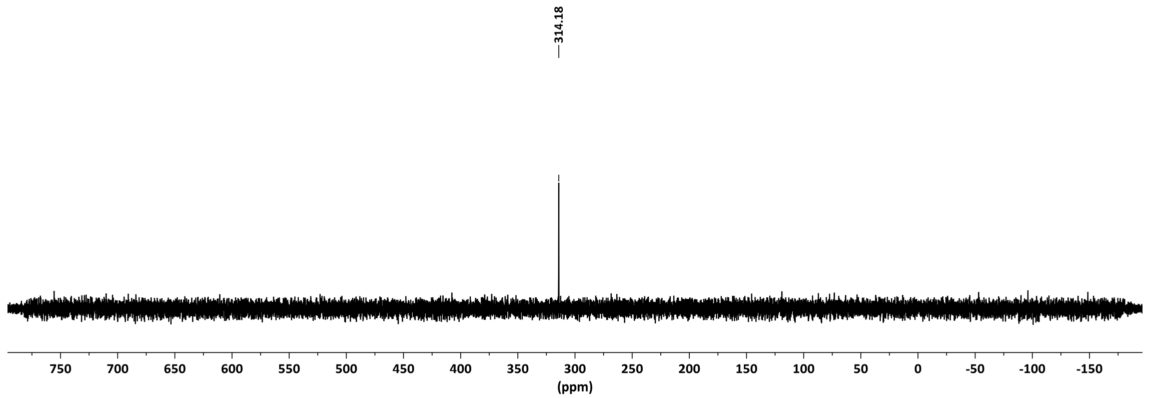

Figure S4: ^{77}Se NMR (114 MHz, $CDCl_3$) spectrum of **1Se**.

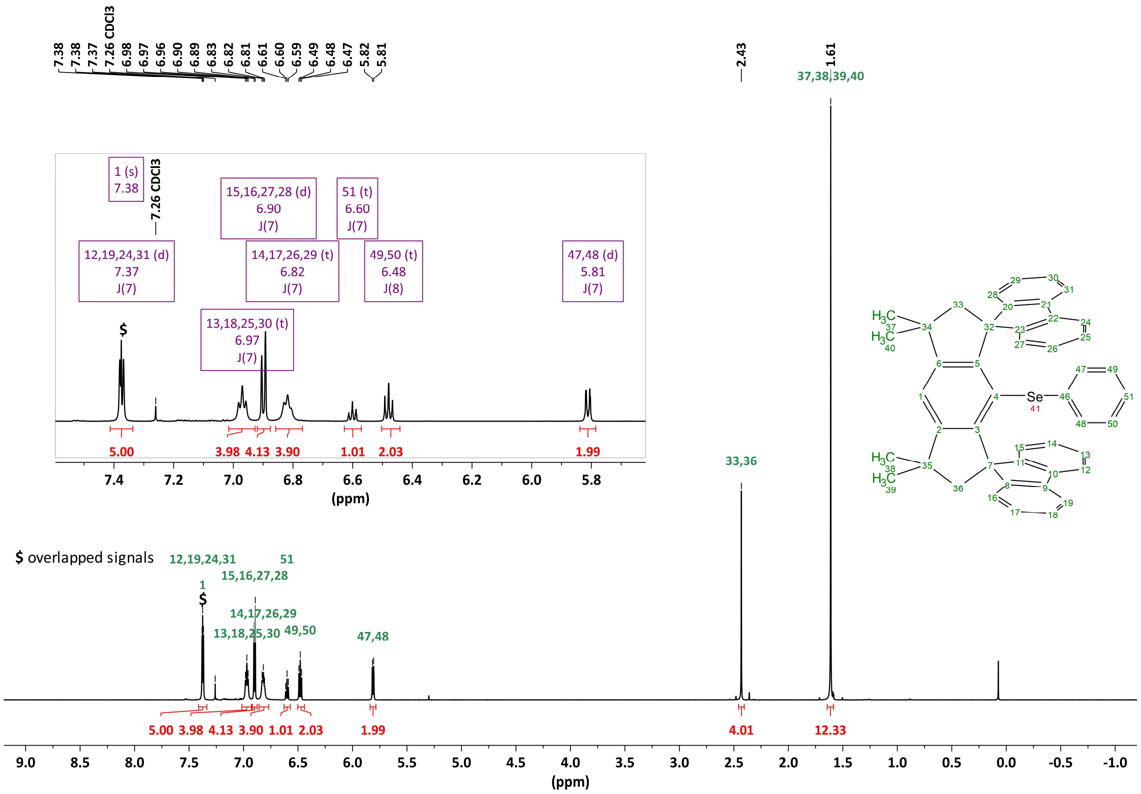

Figure S5: ^{1}H NMR (600 MHz, $CDCl_3$) spectrum of **1Se**.

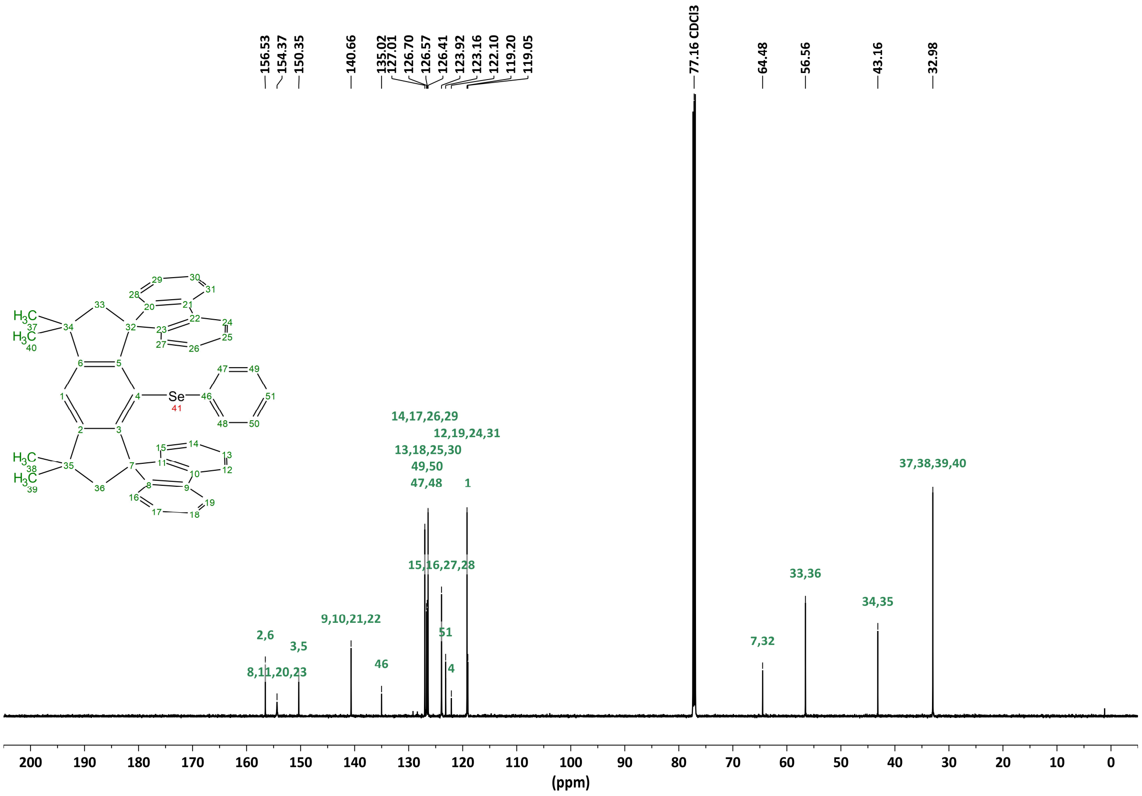

Figure S6. $^{13}C\{^{1}H\}$ NMR (151 MHz, $CDCl_3$) spectrum of **1Se**.

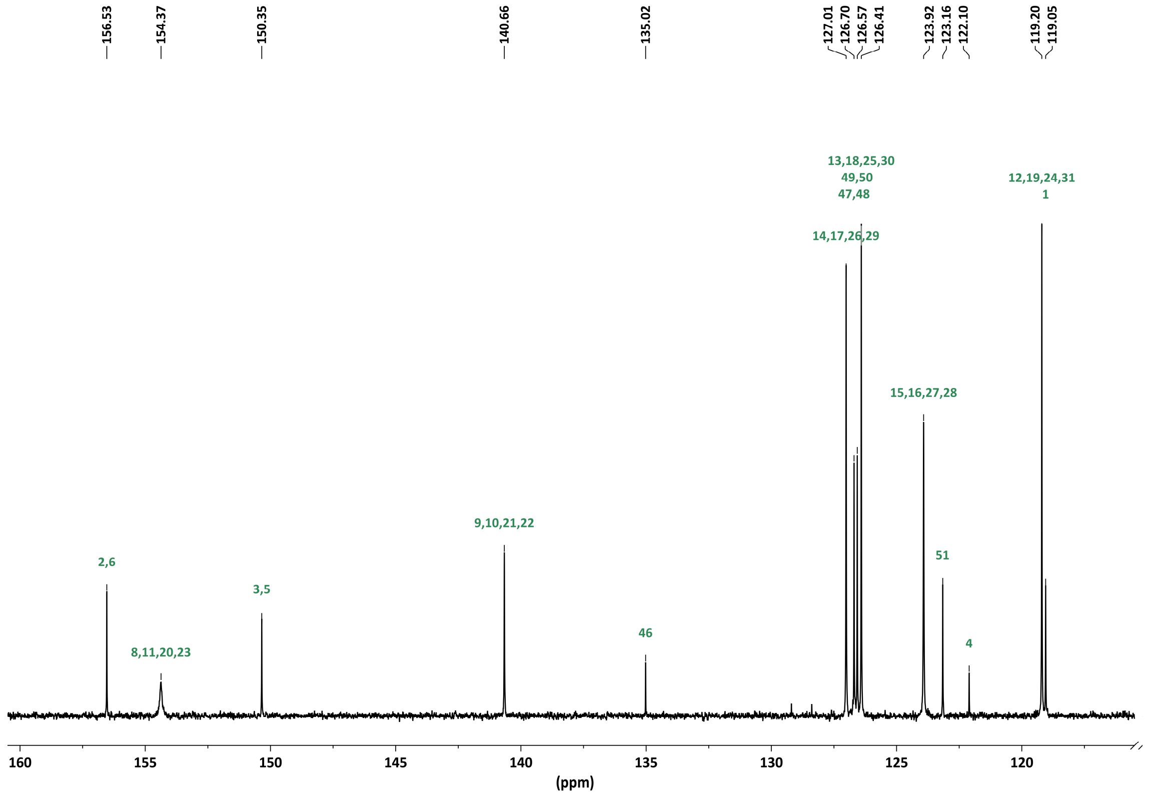

Figure S7. $^{13}C\{^{1}H\}$ NMR (151 MHz, $CDCl_3$) spectrum of **1Se** (downfield area).

Supplementary Note 3. Synthesis and Characterization of M^{S}FluindPhTe (1Te)

A solid mixture of M^{S}FluindLi(THF)$_2$ (1.00 g, 1.50 mmol) and diphenyl ditelluride (655 mg, 1.60 mmol) was suspended in toluene (20 mL) in a Schlenk-tube and stirred for 3 d. Water (1 mL) was added and the mixture was stirred for 15 min. The mixture was combined with water (50 mL) and CH_2Cl_2 (250 mL). The organic phase was separated, filtered through a plug of Celite®-535 and the solvent was removed by rotary evaporation. The solid was washed with diethyl ether (3 × 7 mL) and dried under reduced pressure (10^{-3} mbar), affording the crude product (779 mg, ca. 93 % purity, ca. 67 % crude yield), which contained ca. 7 % of M^{S}FluindH as a side product, as determined by ^{1}H NMR. The crude product was recrystallized from boiling toluene (repeat if necessary). After decanting and drying the yellow solid under reduced pressure (80 °C, 1 d, 10^{-3} mbar), the title compound was obtained in a yield of 53 % (568 mg, 791 µmol). Pale-yellow crystals suitable for single crystal XRD were obtained by recrystallization of **1Te** from boiling toluene.

^{1}H NMR (600 MHz, $CDCl_3$): δ =7.44 (d, J = 7.5 Hz, 4H, 12, 19, 24, 31), 7.39 (s, 1H, 1), 7.03 (t, J = 7.4 Hz, 4H, 13, 18, 25, 30), 6.93 (d, J = 7.5 Hz, 4H, 15, 16, 27, 28), 6.84 (t, J = 7.4 Hz, 4H, 14, 17, 26, 29), 6.71 (t, J = 7.3 Hz, 1H, 51), 6.53 (t, J = 7.6 Hz, 2H, 49, 50), 6.03 (d, J = 7.1 Hz, 2H, 47, 48), 2.42 (s, 4H, 33, 36), 1.59 (s, 12H, 37, 38, 39, 40) ppm.

^{13}C{^{1}H} NMR (151 MHz, $CDCl_3$): δ = 155.73 (2, 6), 155.00 (8, 11, 20, 23), 151.78 (3, 5), 141.22 (9, 10, 21, 22), 132.74 (47, 48), 127.17 (14, 17, 26, 29), 126.97 (49, 50), 126.51 (13, 18, 25, 30), 124.51 (51), 124.02 (15, 16, 27, 28), 119.49 (12, 19, 24, 31), 119.45 (1), 118.45 (46), 108.77 (4), 66.47 (7, 32), 57.07 (33, 36), 42.64 (34, 35), 32.91 (37, 38, 39, 40) ppm.

^{125}Te NMR (189 MHz, $CDCl_3$): δ = 506.7 ppm.

UV-Vis-NIR (CH_2Cl_2, 0.2 mM, 300–1100 nm): λ = 380 nm.

ESI HRMS (m/z): $[M]^{\cdot+}$ calculated for $C_{46}H_{38}Te$: 720.20303, found: 720.20164; $[M+OH]^{+}$ calculated for $C_{46}H_{39}OTe$: 737.20577, found: 737.20398.

Melting point: 341–342 °C.

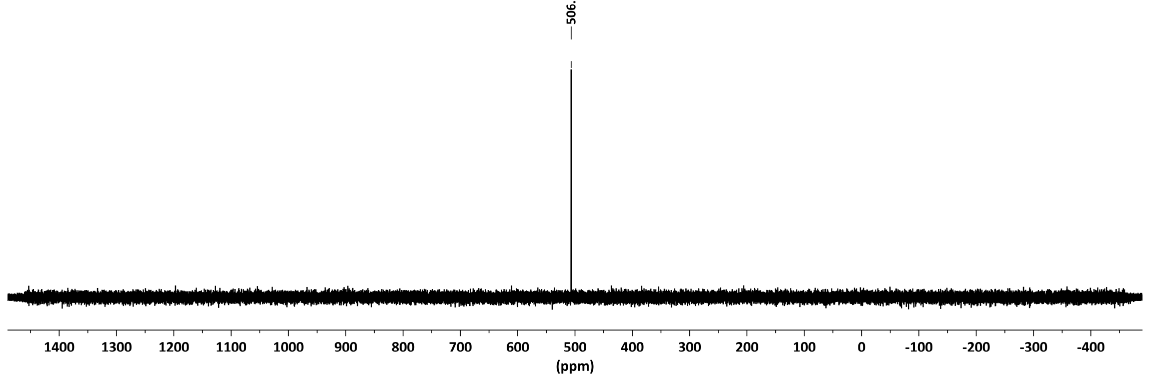

Figure S8. ^{125}Te NMR (189 MHz, $CDCl_3$) spectrum of **1Te**.

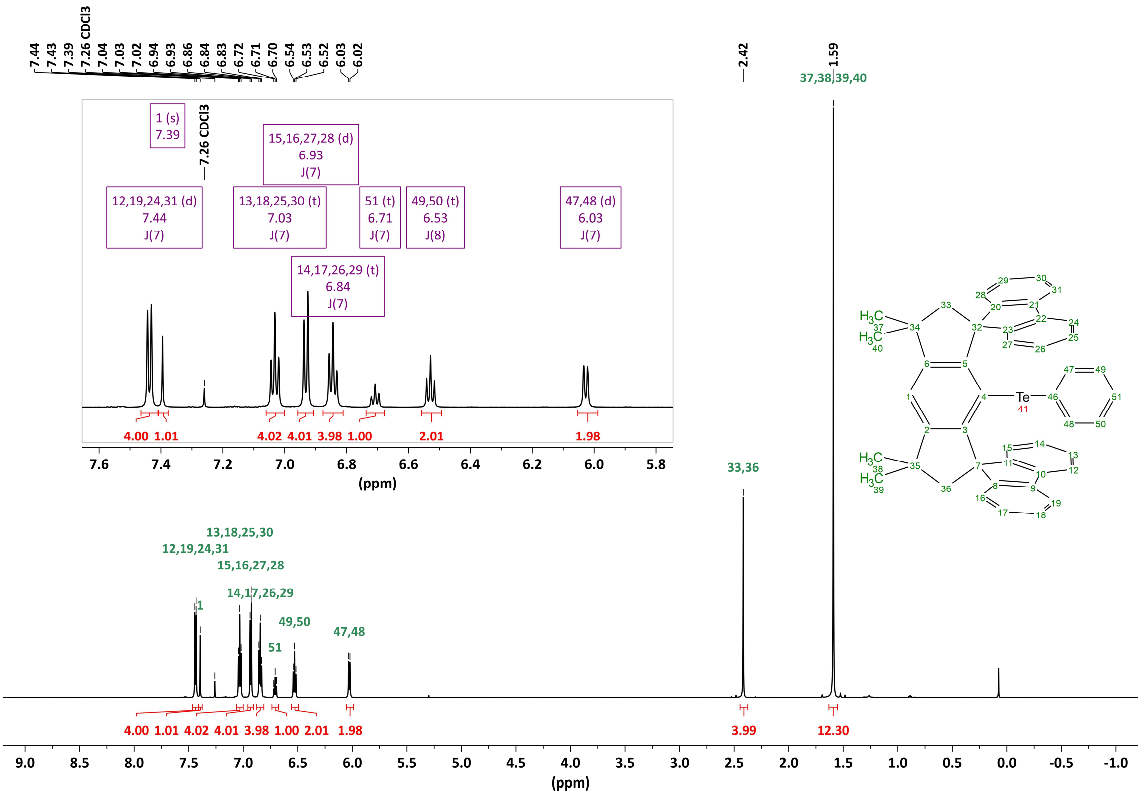

Figure S9. ^{1}H NMR (600 MHz, $CDCl_3$) spectrum of **1Te**.

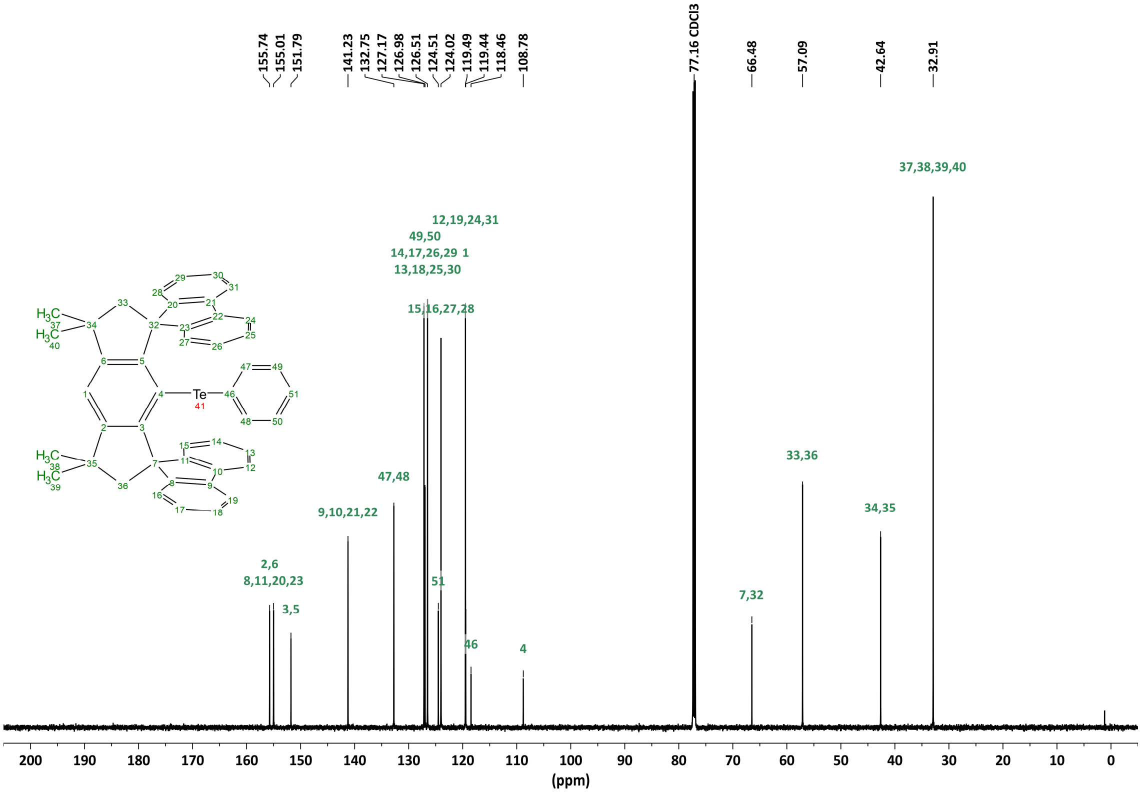

Figure S10. ^{13}C{^{1}H} NMR (151 MHz, $CDCl_3$) spectrum of **1Te**.

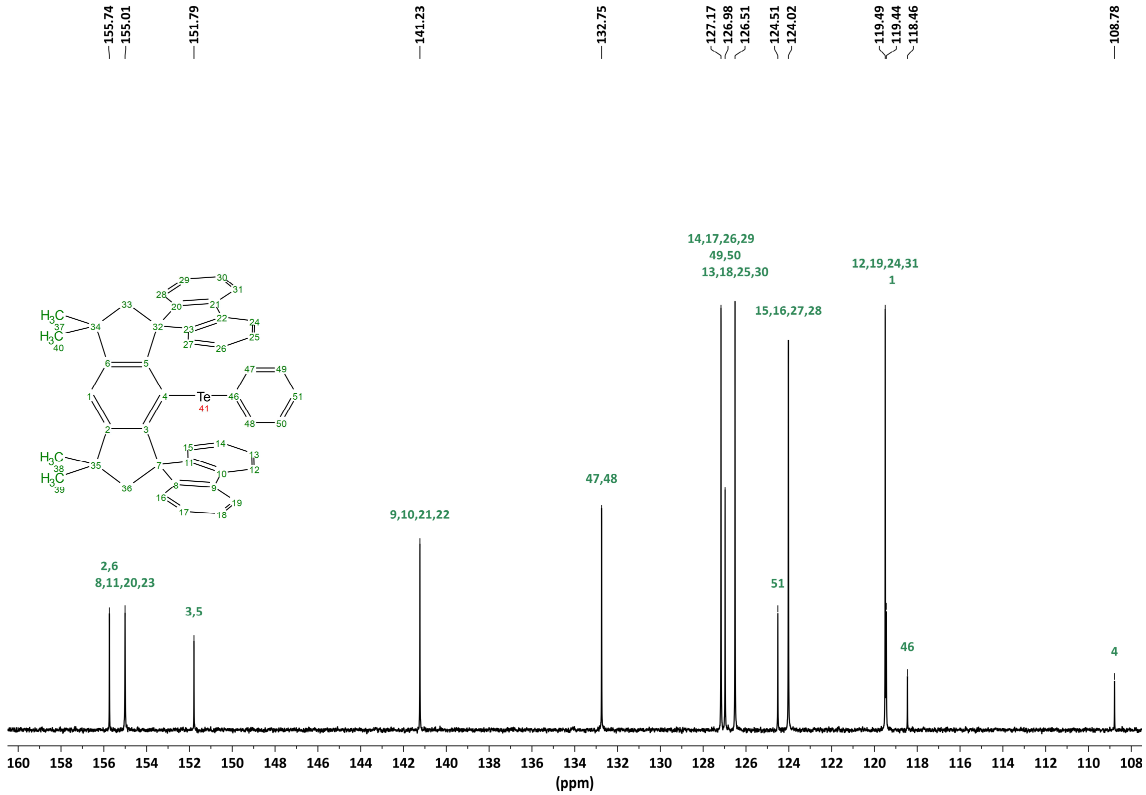

Figure S11. $^{13}C\{^{1}H\}$ NMR (151 MHz, $CDCl_3$) spectrum of **1Te** (downfield area).

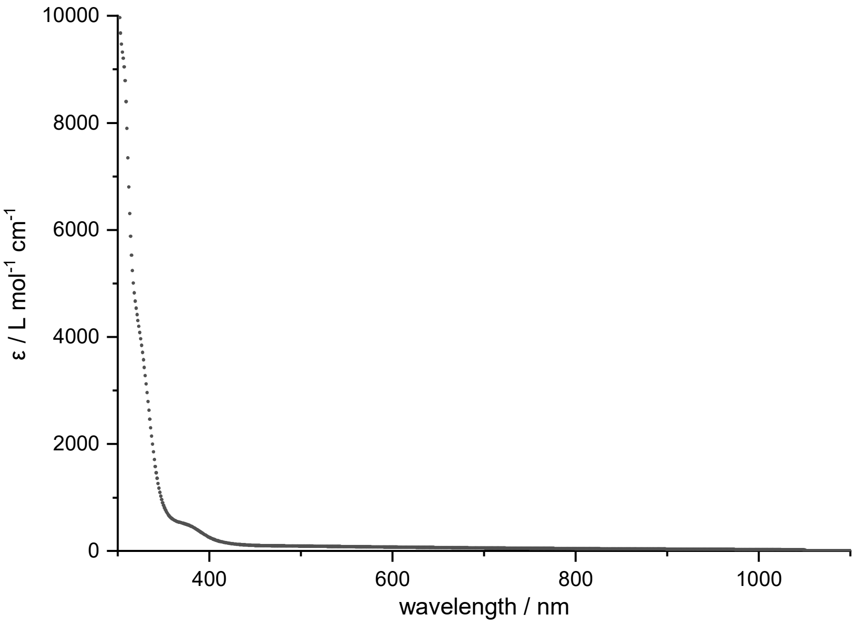

Figure S12. UV-Vis spectrum of **1Te** measured in CH_2Cl_2 (c = 0.2 mM).

Supplementary Note 4. Characterization of [1S][B(C_6F_5)$_4$]

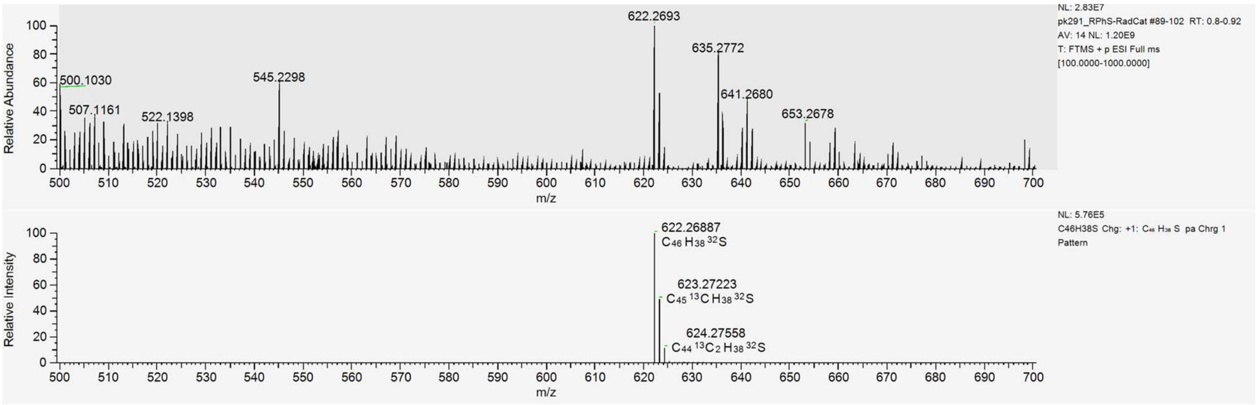

Figure S13. (top) LIFDI HRMS spectrum of [**1S**][B(C_6F_5)$_4$], (bottom) simulation of the isotope pattern of $C_{46}H_{38}S^+$.

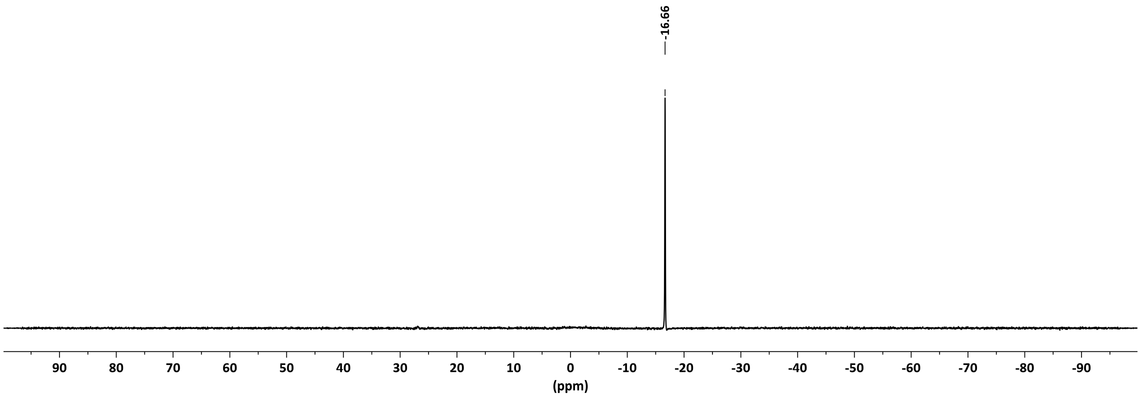

Figure S14. $^{11}B\{^1H\}$ NMR (193 MHz, CD_2Cl_2) spectrum of [**1S**][B(C_6F_5)$_4$].

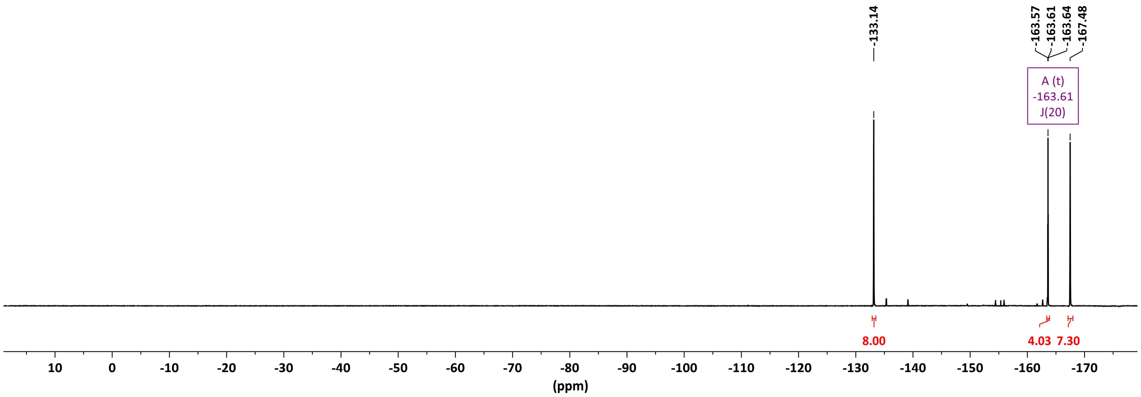

Figure S15. ^{19}F NMR (565 MHz, CD_2Cl_2) spectrum of [**1S**][B(C_6F_5)$_4$].

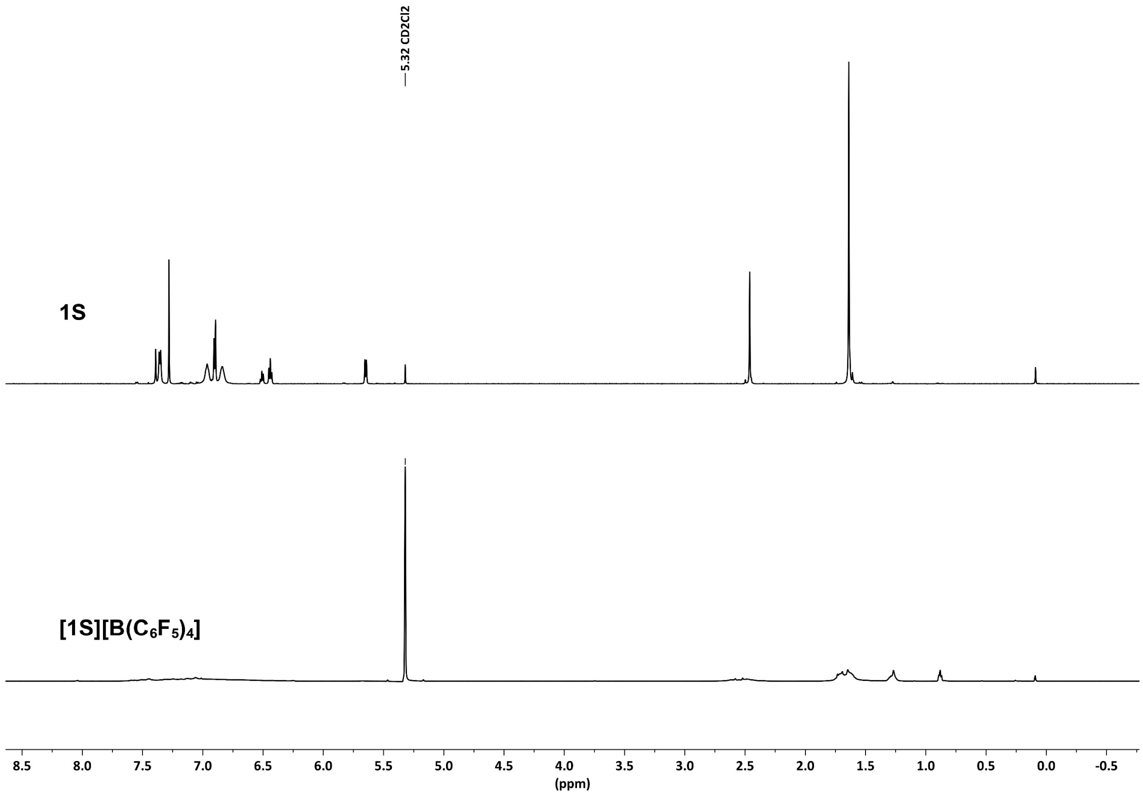

Figure S16. ^{1}H NMR (600 MHz) spectra of [**1S**][B(C_6F_5)$_4$] (bottom, ca. 10 mM in CD_2Cl_2) and **1S** (top, ca. 1 mM in $CDCl_3$).

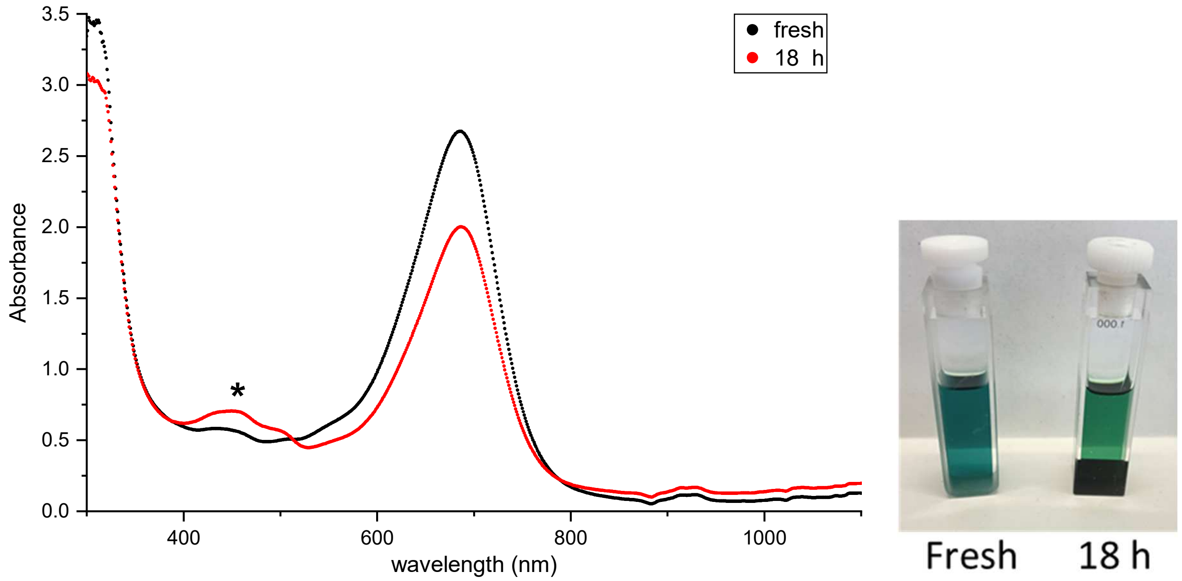

Figure S17. UV-Vis spectra of [**1S**][B(C_6F_5)$_4$] (c = ca. 0.3 mM), sample extracted with DCM/heptane (1:5) (*bands attributed to an unidentified decomposition product).

Supplementary Note 5. Characterisation of [1Se][B(C_6F_5)$_4$]

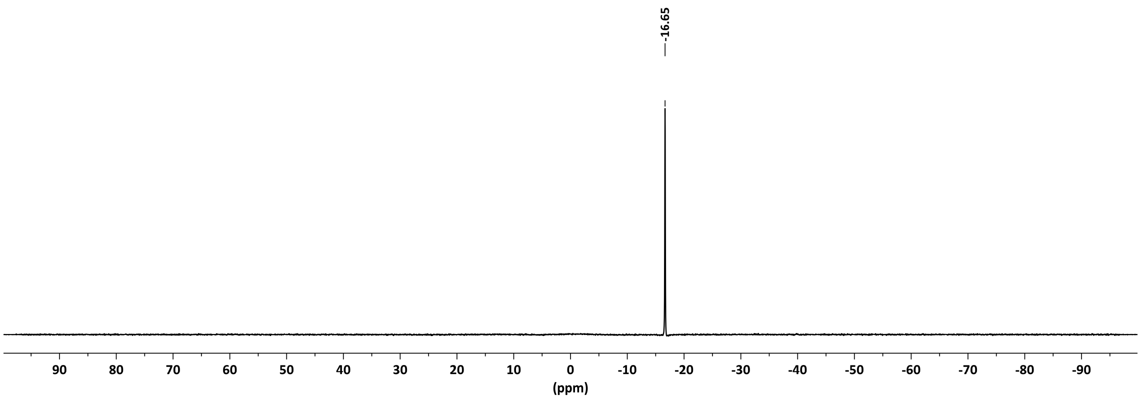

Figure S18. $^{11}B\{^1H\}$ NMR (193 MHz, CD_2Cl_2) spectrum of [**1Se**][B(C_6F_5)$_4$].

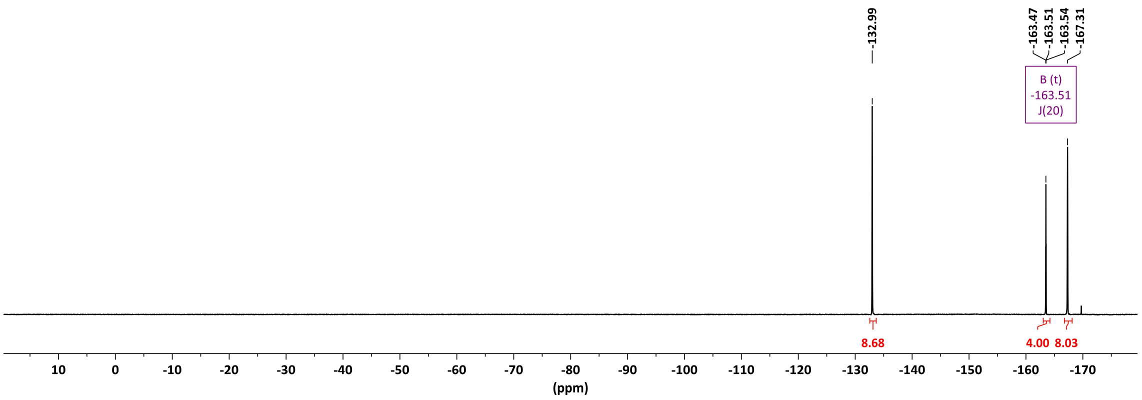

Figure S19. ^{19}F NMR (565 MHz, CD_2Cl_2) spectrum of [**1Se**][B(C_6F_5)$_4$].

Supplementary Note 6. Characterisation of [1Te][B(C_6F_5)$_4$]

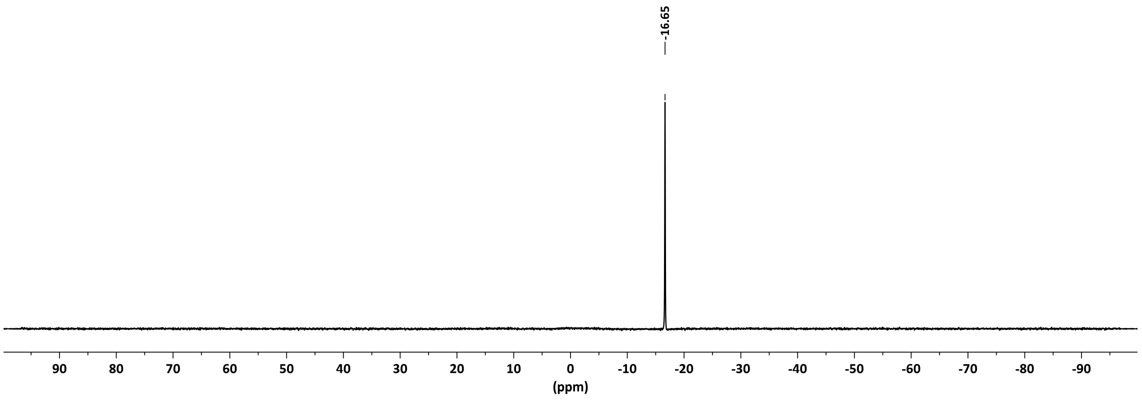

Figure S20. $^{11}B\{^1H\}$ NMR (193 MHz, CD_2Cl_2) spectrum of [**1Te**][B(C_6F_5)$_4$].

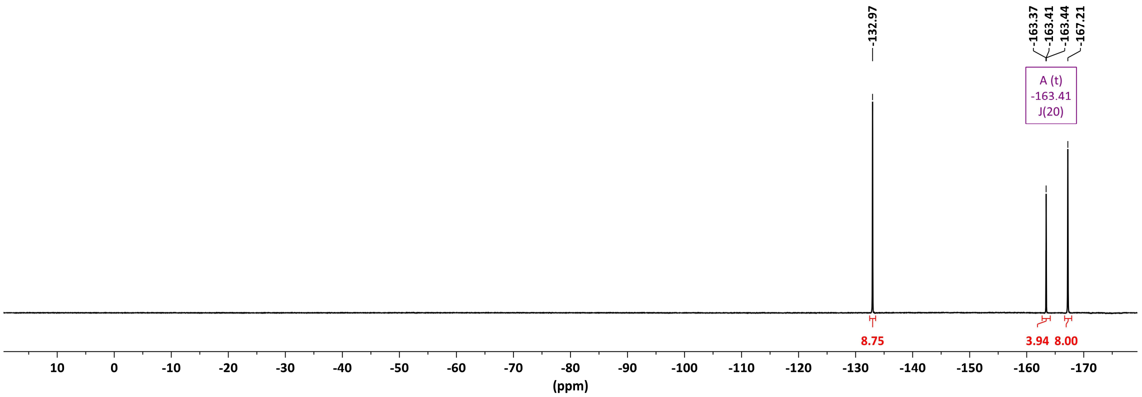

Figure S21. ^{19}F NMR (565 MHz, CD_2Cl_2) spectrum of [**1Te**][$B(C_6F_5)_4$].

Supplementary Note 7. Synthesis and Characterisation of [1Te][SbF_6]

A solid mixture of **1Te** (30.0 mg, 41.8 µmol) and NO[SbF_6] (11.1 mg, 41.8 mmol) was placed in a Schlenk-tube, suspended in CH_2Cl_2 (5 mL) (propionitrile gave similar results) and stirred for 16 h at room temperature. The solution was filtered using a syringe filter, ^{n}hexane (5 mL) was layered on top of the solution and after 2 d the solution was decanted off. The remaining dark purple solid contained crystals suitable for single crystal XRD. Drying the solid under reduced pressure (10^{-3} mbar) afforded [**1Te**][SbF_6] (16.8 mg, 17.6 µmol, 42 %).

The compound is NMR silent in CD_2Cl_2.

ESI HRMS (m/z): $[M+OH]^+$ calculated for $C_{46}H_{39}OTe$: 737.20577, found: 737.20597.

UV-Vis-NIR (CH_2Cl_2, 300—1100 nm): $\lambda(\varepsilon)$ = 307 (10000), 340 (3300), 553 (3200), 835 (2500) nm (L mol^{-1} cm^{-1}).

Melting point: 315° C (decomposition to brown solid).

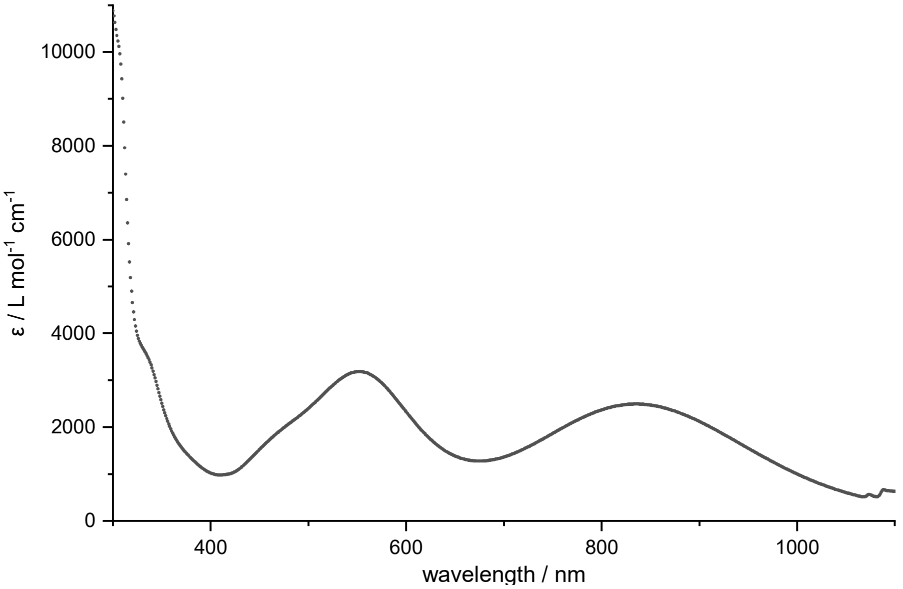

Figure S22. UV-Vis-NIR spectrum of [**1Te**][SbF_6] measured in CH_2Cl_2 (c = 0.2 mM).

Supplementary Note 8. Synthesis and Characterisation of M^{S}FluindPhTeF$_2$

Compound **1Te** (250 mg, 348 µmol) was dissolved in CH_2Cl_2 (10 mL) within a Schlenk-tube and cooled to −40 °C while stirring. XeF_2 (59.0 mg 349 µmol) was added, and the solution was warmed up to room temperature while stirring for 1 h. The volatiles were removed under reduced pressure. The remaining colourless solid was washed with ^{n}pentane (3×3 mL) and dried under reduced pressure (10^{-3} mbar, 16 h, 60 °C) affording the title compound in 98 % yield (258 mg, 341 µmol). Colourless crystals suitable for single crystal XRD were obtained by slowly evaporating a solution of M^{S}FluindPhTeF$_2$ in CH_2Cl_2/pentane.

^{1}H NMR (600 MHz, CD_2Cl_2): δ = 7.63 (d, J = 7.1 Hz, 1H, 12, 19), 7.51 (s, 1H, 1), 7.42 (d, J = 6.9 Hz, 1H, 15, 16), 7.33 (t, J = 7.0 Hz, 2H, 13, 18), 7.30 (t, J = 7.0 Hz, 1H, 14, 17), 7.10 (t, J = 7.4 Hz, 2H, 26, 29), 7.04 (d, J = 7.7 Hz, 3H, 27, 28), 7.02 (t, J = 7.0 Hz, 1H, 25, 30), 6.99 (t, J = 7.3 Hz, 1H, 51), 6.86 (d, J = 7.4 Hz, 2H, 24, 31), 6.78 (t, J = 7.6 Hz, 2H, 49, 50), 6.25 (d, J = 7.9 Hz, 2H, 47, 48), 2.59 (s, 2H, 36), 2.11 (s, 2H, 33), 1.66 (s, 6H, 38, 39), 1.44 (s, 6H, 37, 40) ppm.

^{13}C{^{1}H} NMR (151 MHz, CD_2Cl_2): δ = 159.51 (6), 157.60 (2), 154.66 (8, 11), 154.43 (20, 23), 145.75 (3), 142.23 (5), 140.82 (4), 140.13 (9, 10), 139.33 (21, 22), 135.07 (46), 132.72, 132.67, 132.61, 129.56 (49, 50), 129.33 (51), 128.18 (14, 17), 128.09 (13, 18), 126.93 (26, 29), 126.85 (25, 30), 126.74 (15, 16), 124.46 (27, 28), 121.31 (24, 31), 121.06 (1), 120.50 (12, 19), 66.14 (32), 65.86 (7), 62.89 (33), 58.93 (36), 53.84, 43.70 (35), 43.23 (34), 32.75 (38, 39), 32.07 (37, 40) ppm.

^{19}F NMR (565 MHz, CD_2Cl_2): δ = −127.02 (d, J = 472.1 Hz).

^{125}Te NMR (189 MHz, CD_2Cl_2): δ = 1164.30 (t, J = 470.8 Hz).

ESI HRMS(m/z): $[M+Na]^{+}$ calculated for $C_{46}H_{38}F_2NaTe$: 781.19013, found: 781.18956; $[M+K]^{+}$ calculated for $C_{46}H_{38}F_2KTe$: 797.16354, found: 797.16423.

Melting point: 275 °C (decomposition to brown solid).

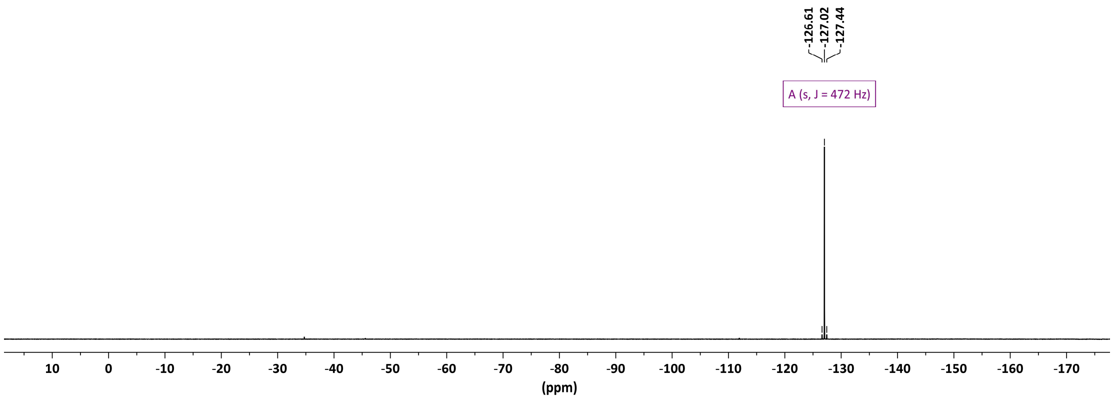

Figure S23. ^{19}F NMR (565 MHz, CD_2Cl_2) spectrum of M^{S}FluindPhTeF$_2$.

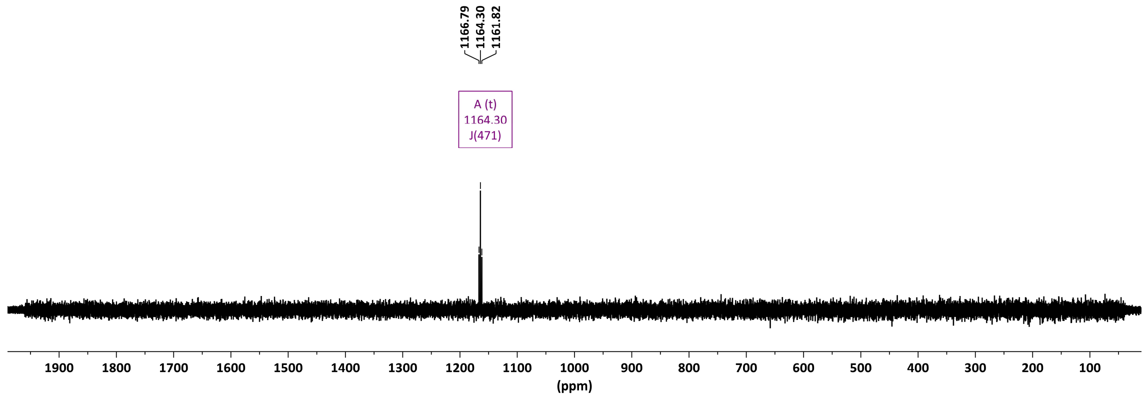

Figure S24. [125]Te NMR (193 MHz, CD_2Cl_2) spectrum of M[S]FluindPhTeF_2.

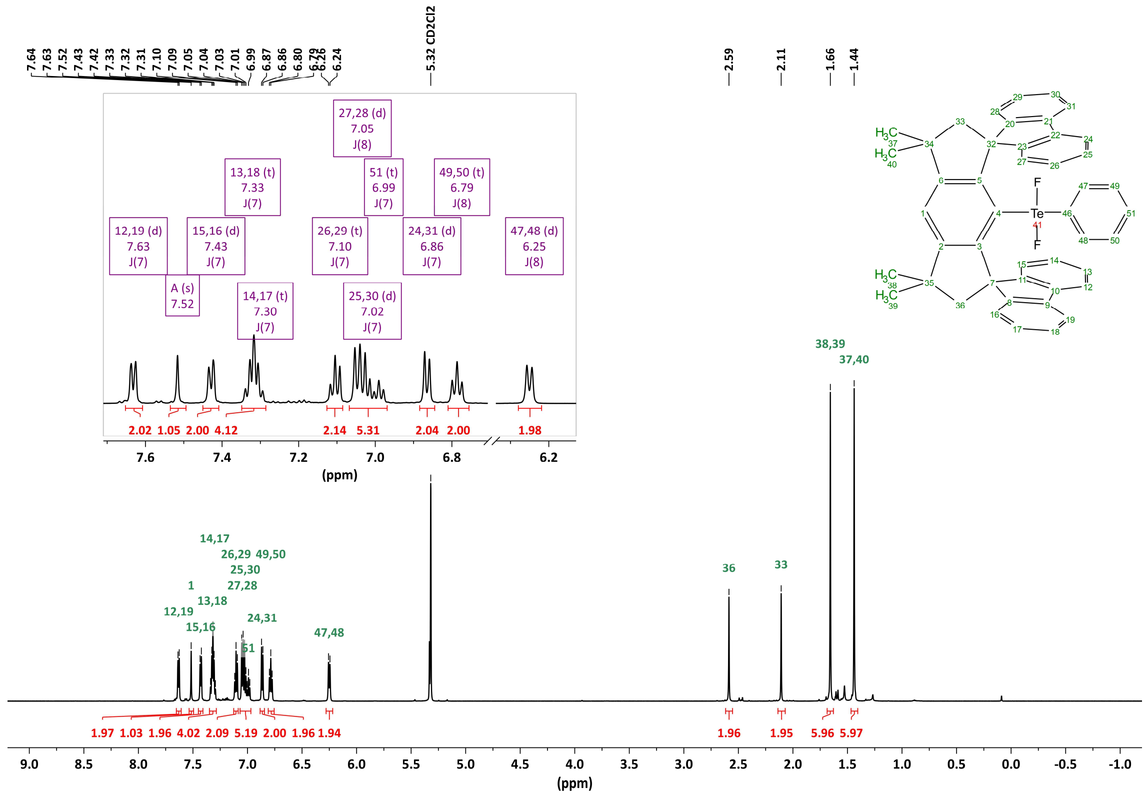

Figure S25. ^{1}H NMR (600 MHz, CD_2Cl_2) spectrum of M[S]FluindPhTeF_2.

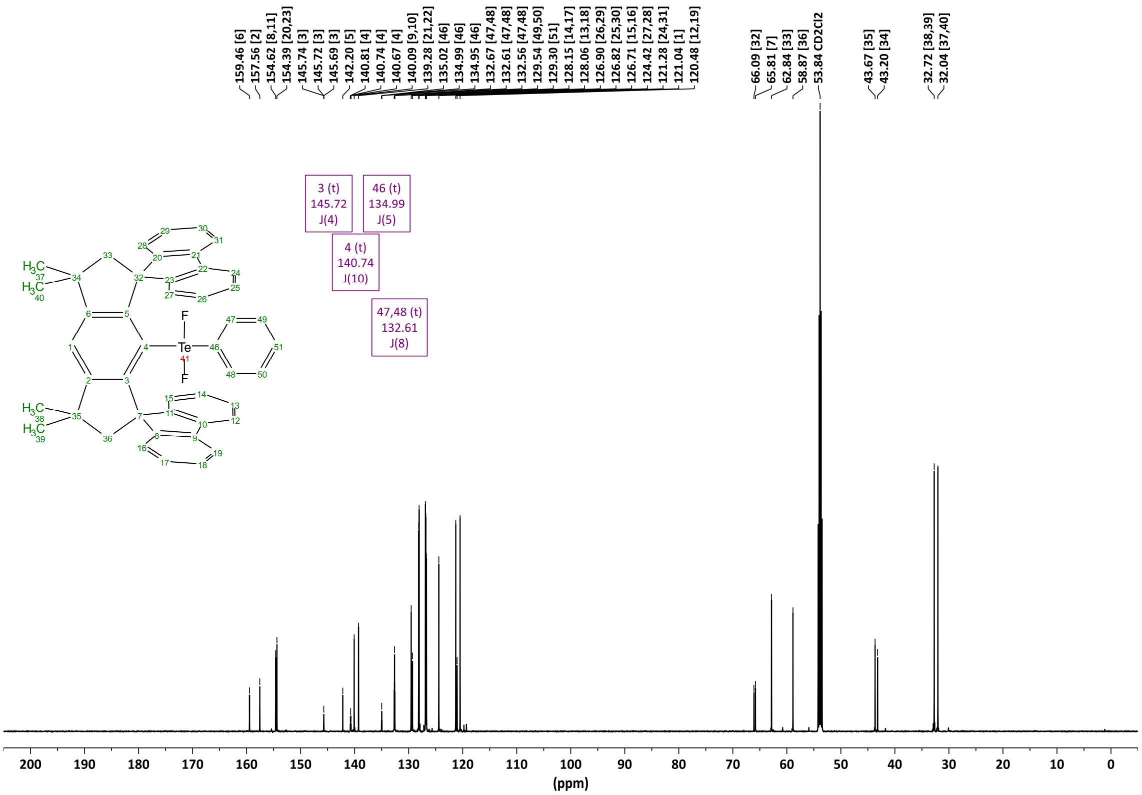

Figure S26. $^{13}C\{^{1}H\}$ NMR (151 MHz, CD_2Cl_2) spectrum of M^{S}FluindPhTeF_2.

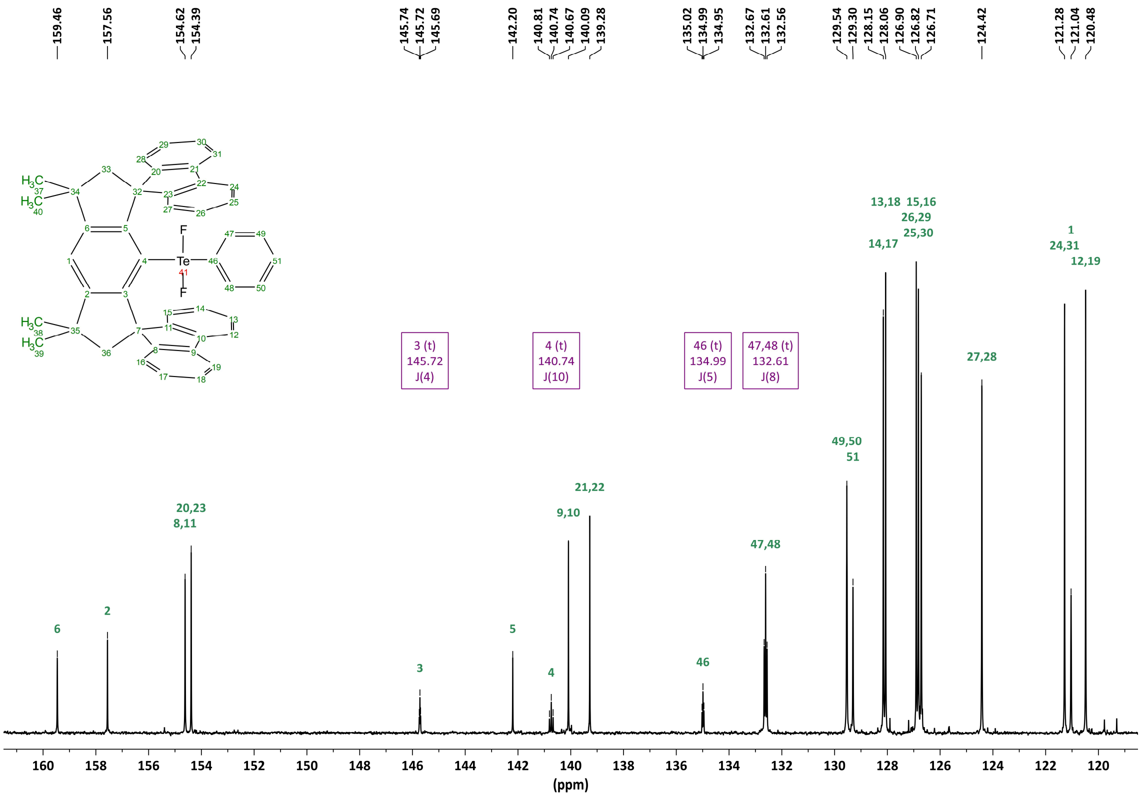

Figure S27. $^{13}C\{^{1}H\}$ NMR (151 MHz, CD_2Cl_2) spectrum of M^{S}FluindPhTeF_2 (downfield area).

Supplementary Note 9. Stability of [1E][B(C_6F_5)$_4$] (E = S, Se, Te) in Solution

Table S28. Stability of [**1S**][B(C_6F_5)$_4$] in CH_2Cl_2 (c = ca. 0.1 mM) when stored at room temperature in the dark or under ambient light, monitored by UV-Vis measurements. (*Attributed to an unidentified, transient, decomposition product).

	Darkness		Light	
t / d	A (λ = 500 nm)*	A (λ = 697 nm)	A (λ = 500 nm)*	A (λ = 697 nm)
0	0.31	0.99	0.32	**1.00**
1	0.23	0.87	0.25	**0.54**
2	0.20	0.82	0.23	0.34
6	0.16	0.73		
13	0.13	0.61		
24	0.11	0.58		

Table S29. Stability of [**1Se**][B(C_6F_5)$_4$] in CH_2Cl_2 (c = ca. 0.1 mM) when stored at room temperature in the dark or under ambient light, monitored by UV-Vis measurements.

t / d	Absorbance (λ = 1068 nm) — Darkness	Absorbance (λ = 1068 nm) — Light
0	0.48	**0.40**
5	0.45	0.32
10		0.27
14	0.45	
22		**0.20**
29	0.44	

Table S30. Stability of [**1Te**][B(C_6F_5)$_4$] in CH_2Cl_2 (c = ca. 0.1 mM) when stored at room temperature in the dark or under ambient light, monitored by UV-Vis measurements.

t / d	Absorbance (λ = 553 nm) — Darkness	Absorbance (λ = 553 nm) — Light
0	0.50	**0.50**
7		0.36
14	0.48	0.31
21	0.49	0.29
28		**0.26**

Supplementary Note 10. Computational Results

DFT calculations were performed for [**1E**][$B(C_6F_5)_4$] (E = S, Se, Te) compounds using ORCA version 6.0.0.[5,6] Experimentally obtained crystal structures of [**1Se**][$B(C_6F_5)_4$] and [**1Te**][$B(C_6F_5)_4$] were used as starting points for geometry optimisations. Structures of all compounds were optimised without counter ions. Calculations were performed utilising hybrid pbe0[7] functional, along with atom-pairwise dispersion correction with the Becke-Johnson damping scheme (D3BJ).[8,9] For the inclusion of relativistic effects zeroth-order regular approximation (ZORA)[10,11] was used along with relativistically contracted triple ζ ZORA-def2-tzvp basis set for C, H, S, Se atoms and old-ZORA-TZVP basis set for Te atoms.[12,13] The RIJCOSX approximation was used to speed up calculations.[14] To account for solvation effects, the conductor-like polarisable continuum model (CPCM)[15] with CH_2Cl_2 solvent was used. TDDFT calculations were performed for the first 100 transitions.

The wavefunction file was used for a topological analysis of the electron density according to the Atoms-In-Molecules partitioning scheme[16] using AIMAll.[17] The Independent gradient model based on Hirshfeld partition (IGMH)[18,19] analysis were obtained with Multiwfn_3.8.[20] AIM bond critical points and bond paths and IGMH-iso-surfaces are displayed with VMD.[21]

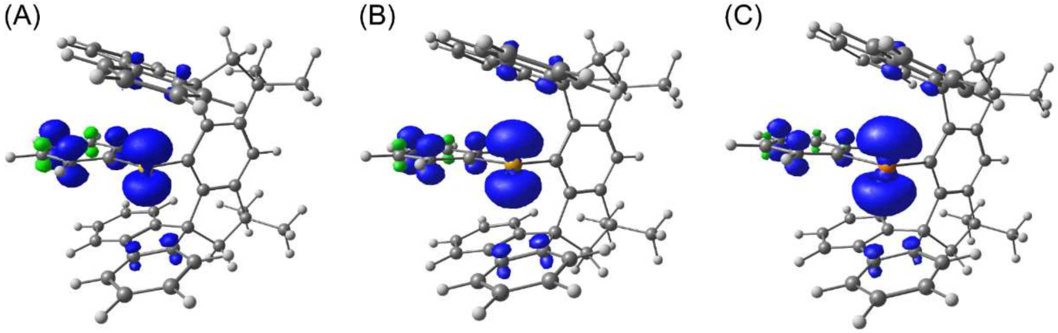

Figure S31. Mulliken spin densities for [**1E**]$^{•+}$ (E = S (A), Se (B), Te (C); blue – positive, green – negative, contour value 0.005).

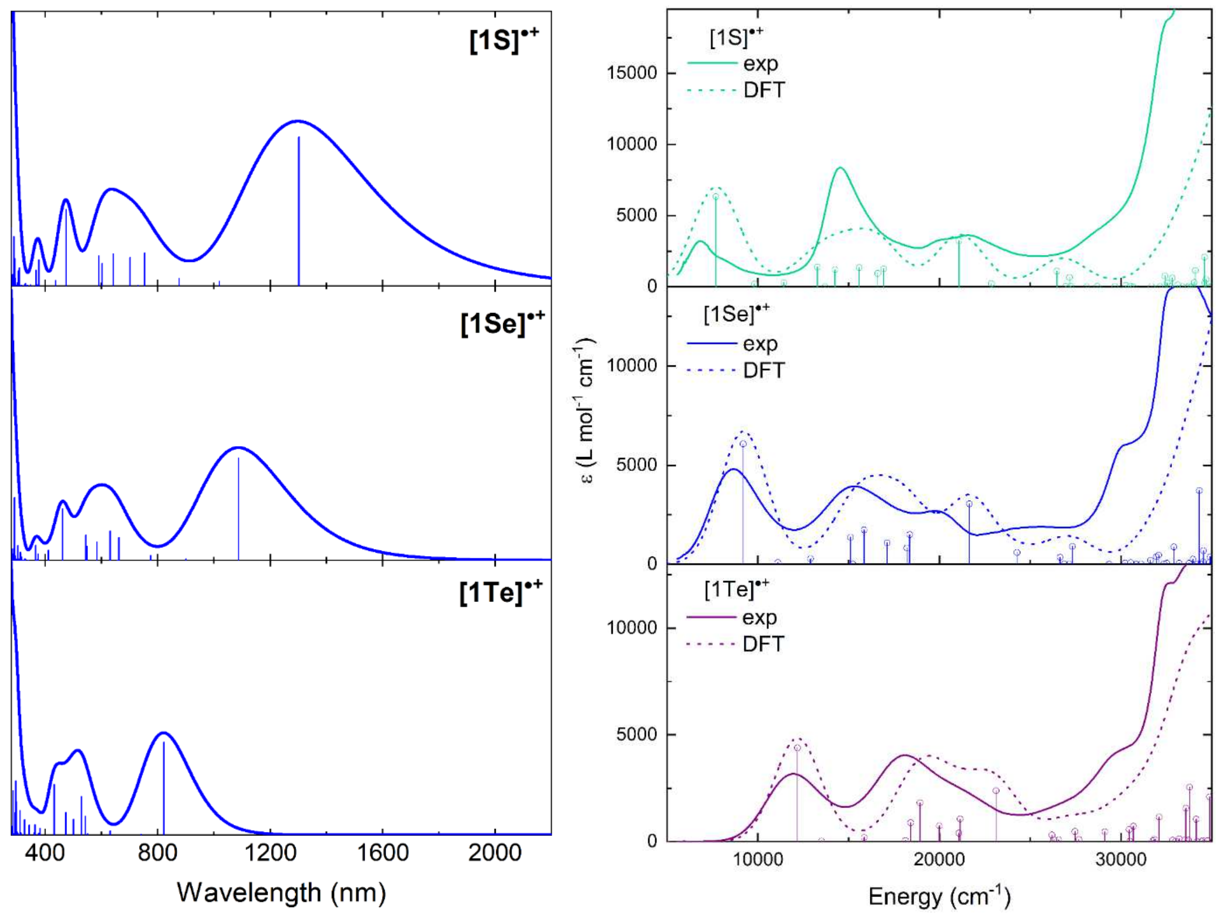

Figure S32. TDDFT calculated electronic absorption spectra of [**1E**]$^{\bullet+}$ (E = S, Se, Te) (left). Comparison of experimental (solid line) and TDDFT calculated (dotted line) electronic absorption spectra of [**1E**]$^{\bullet+}$ (E = S, Se, Te) (right). Individual transitions are depicted by vertical lines.

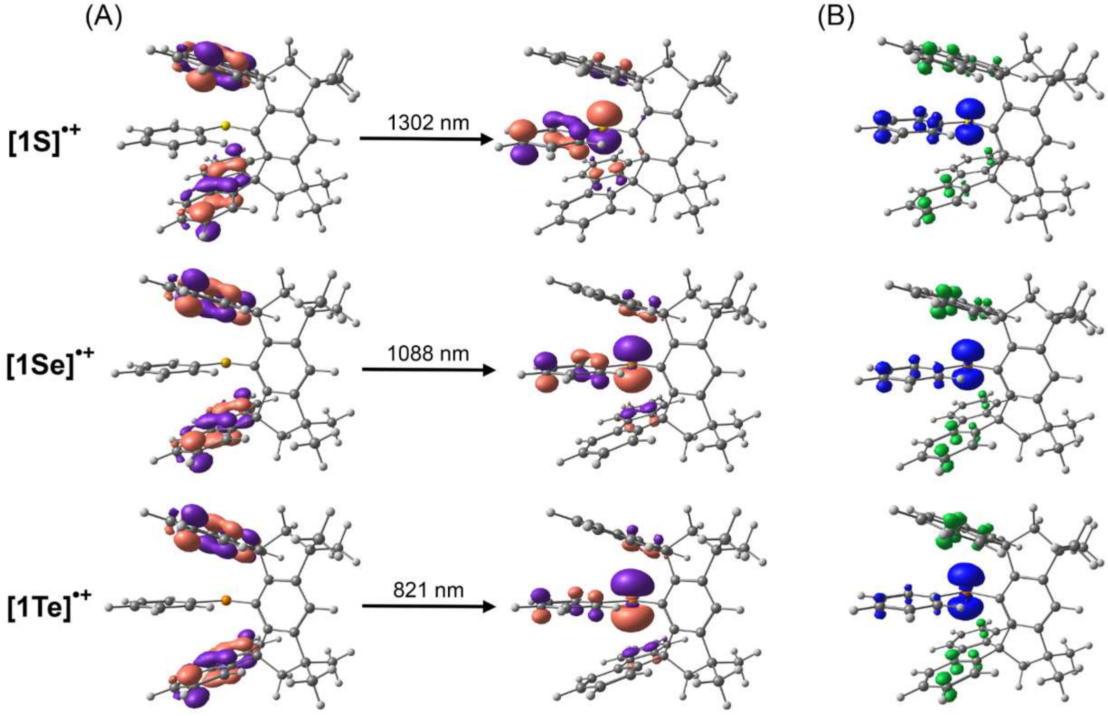

Figure S33. (A) Natural transition orbitals (orange – positive, purple – negative, contour value 0.05) and (B) transition difference densities (blue – positive, green – negative, contour value 0.005) for lowest energy transitions, for [**1E**][B(C_6F_5)$_4$] (E = S, Se, Te).

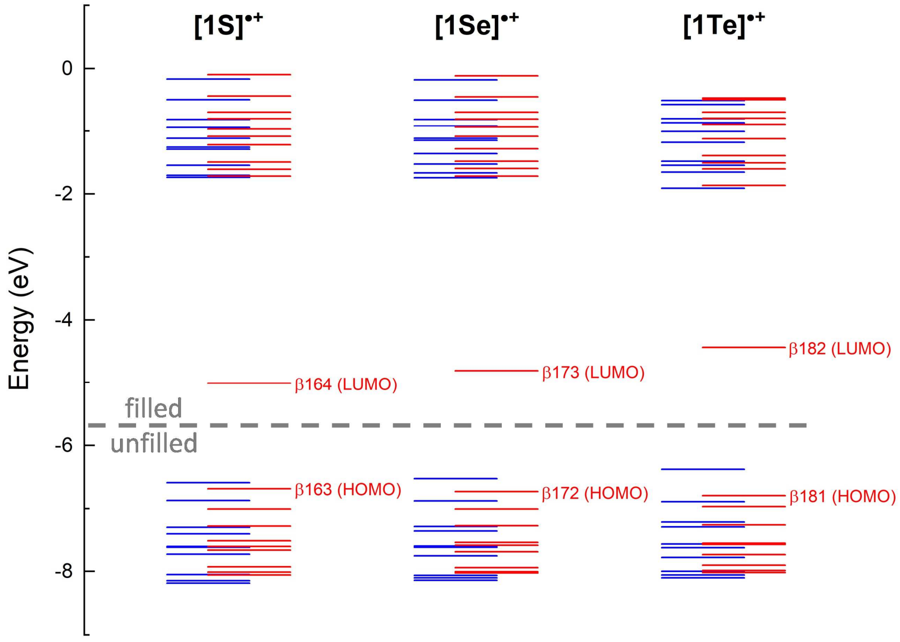

Figure S34. MO diagram of [**1E**]$^{\bullet+}$ (E = S, Se, Te, blue lines – α orbitals, red lines – β orbitals).

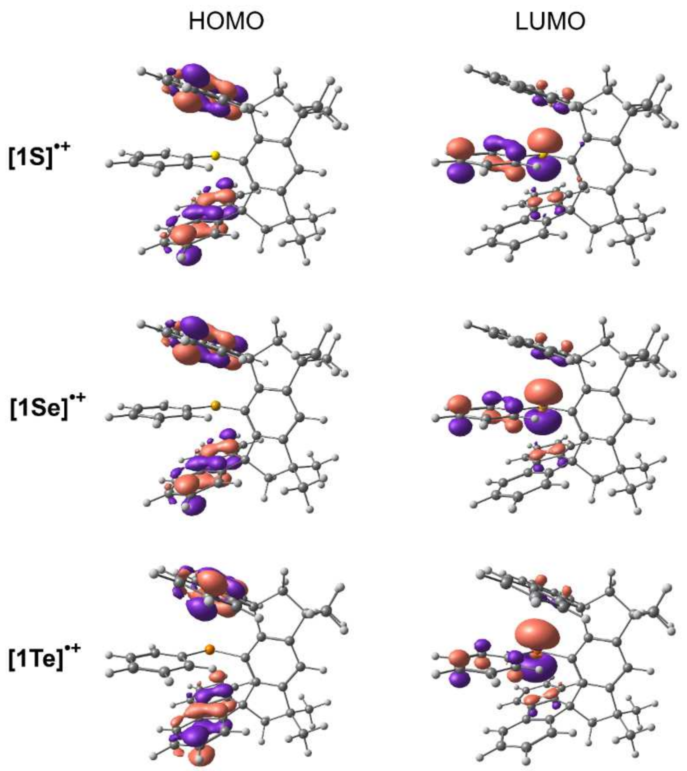

Figure S35. HOMO and LUMO β orbitals of [**1E**]$^{•+}$ (E = S, Se, Te, orange – positive, purple – negative, contour value 0.05).

Table S36. TDDFT calculated transitions for [**1S**]$^{•+}$ (first 10 transitions).

	Transition	Coefficient	Energy (cm^{-1})
1	163β -> 164β	0.986681	7682.9
2	162β -> 164β	0.976102	9807.7
3	161β -> 164β	0.995810	11421.1
4	160β -> 164β	0.954311	13271.7
5	159β -> 164β<br>158β -> 164β	0.807884<br>0.156338	13702.1
6	158β -> 164β<br>159β -> 164β	0.829022<br>0.158955	14244.6
7	155β -> 164β<br>157β -> 164β	0.597081<br>0.326540	15567.9
8	156β -> 164β	0.949244	16601.4
9	157β -> 164β<br>155β -> 164β	0.634378<br>0.349789	16927.1
10	154β -> 164β	0.940134	21075.2

Table S37. TDDFT calculated transitions for [**1Se**]$^{\bullet+}$ (first 10 transitions).

	Transition	Coefficient	Energy (cm^{-1})
1	172β -> 173β	0.988110	9188.9
2	171β -> 173β	0.981685	11100.9
3	170β -> 173β	0.992510	12899.6
4	169β -> 173β	0.960700	15102.1
5	168β -> 173β<br>167β -> 173β	0.765450<br>0.205652	15231.4
6	167β -> 173β<br>168β -> 173β	0.780618<br>0.199675	15845.9
7	166β -> 173β<br>165β -> 173β	0.492930<br>0.455833	17121.5
8	164β -> 173β	0.946867	18214.4
9	165β -> 173β<br>166β -> 173β	0.506090<br>0.472084	18350.1
10	163β -> 173β	0.944436	21643.9

Table S38. TDDFT calculated transitions for [**1Te**]$^{\bullet+}$ (first 10 transitions).

	Transition	Coefficient	Energy (cm^{-1})
1	181b -> 182b	0.990791	12162.5
2	180b -> 182b	0.987382	13495.6
3	179b -> 182b	0.989729	15826.3
4	178b -> 182b<br>176b -> 182b	0.839427<br>0.131246	18113.3
5	177b -> 182b	0.942779	18388.6
6	176b -> 182b<br>178b -> 182b	0.835614<br>0.111631	18912.8
7	175b -> 182b	0.958711	19957.4
8	173b -> 182b	0.904180	21043.9
9	174b -> 182b	0.949380	21130.0
10	172b -> 182b	0.935443	23090.3

Table S39. Topological bond properties from AIM analysis of **[1S]**$^{\bullet+}$, **[1Se]**$^{\bullet+}$ and **[1Te]**$^{\bullet+}$.

Species	d [Å]	ρ(r) [eÅ^{-3}]	∇^2ρ(r) [eÅ^{-5}]	ε	G/ρ(r) [a.u.]	H/ρ(r) [a.u.]	δ
			**[1S]**$^{\bullet+}$ (**q$_{Bader}$(S) = 0.34 e**)				
**S1–C9**	1.762	1.37	–9.5	0.02	0.29	–0.77	1.08
**S1–C12**	1.715	1.49	–11.6	0.21	0.32	–0.86	1.23
**S1–C34**	2.966	0.13	1.0	0.52	0.56	0.02	0.10
**S1–C55**	2.962	0.13	1.1	0.52	0.56	0.02	0.10
			**[1Se]**$^{\bullet+}$ (**q$_{Bader}$(Se) = 0.62 e**)				
**Se1–C9**	1.909	1.10	–5.0	0.02	0.35	–0.67	1.03
**S1–C12**	1.867	1.18	–5.3	0.19	0.40	–0.72	1.15
**S1–C34**	3.005	0.13	1.0	0.63	0.52	0.00	0.12
**S1–C55**	3.000	0.14	1.0	0.63	0.52	0.00	0.12
			**[1Te]**$^{\bullet+}$ (**q$_{Bader}$(Te) = 1.05 e**)				
**Te1–C44**	2.080	0.88	0.4	0.16	0.55	–0.52	1.05
**Te1–C69**	2.114	0.83	–0.1	0.04	0.49	–0.50	0.98
**Te1–C58**	3.103	0.14	0.9	0.82	0.49	–0.03	0.12
**Te1–C74**	3.101	0.14	0.9	0.83	0.49	–0.03	0.12

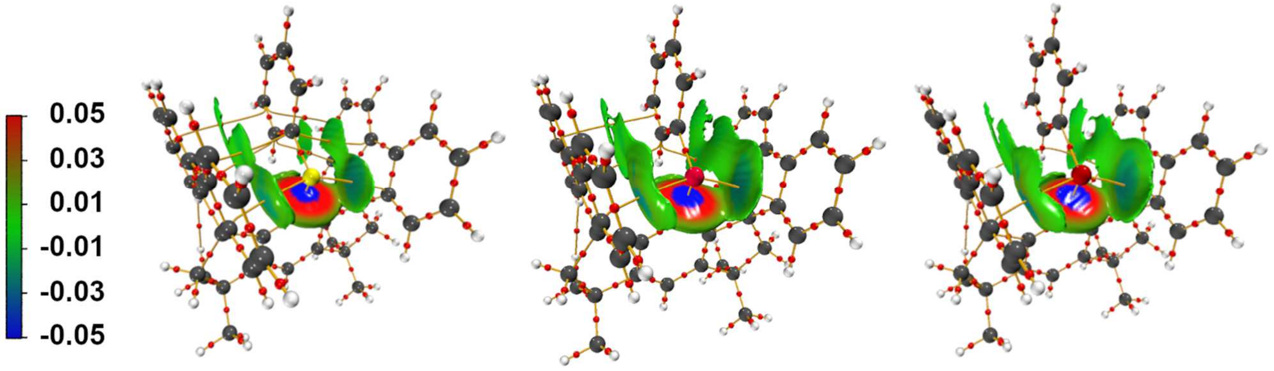

Figure S40. AIM molecular graph of **[1S]**$^{\bullet+}$ (left), **[1Se]**$^{\bullet+}$ (middle) and **[1Te]**$^{\bullet+}$ (right) with bond critical points as red spheres and bond paths in orange as well as IGM based on a Hirshfeld partition of the molecular density of **[1E]**$^{\bullet+}$: Fragment 1 is the EPh-moiety (E = S, Se, Te) and fragment 2 includes all other atoms. IGMH *iso*-surfaces at s(r) = 0.005 colour coded with sign(λ2)ρ in a. u. Blue surfaces refer to attractive forces and red to repulsive forces. Green indicates weak interactions.

Supplementary Note 11. Cyclic Voltammetry

Cyclic voltammetry studies were performed in a V-tube electrochemical cell. The CV data was recorded at room temperature (*ca.* 25 °C) using an Autolab PGSTAT 101 (Metrohm) Electrochemical Workstation. A three-electrode configuration was used with a glassy carbon (CHInstruments CHI104) acting as working electrode (WE), a Pt wire acting as counter electrode (CE) and a Ag/AgCl pseudo reference electrode (pRE). Before every measurement, the counter electrode was polished to a mirror-like appearance with diamond paste (1µm) and carefully rinsed with deionised water, sonicated, and then rinsed with HPLC grade acetone. The analyte solutions (1 mM) were prepared in dry and degassed CH_2Cl_2 (10 mL), using [^{n}Bu$_4$N][PF$_6$] (0.1 M; from Sigma Aldrich, used as received) as supporting electrolyte. The potentials are reported against the ferrocene/ferrocenium redox couple (FcH/FcH$^+$), ferrocene was added as an internal standard at the end of the measurements. Plotting convention: IUPAC [direction of the scan: toward positive potentials (to the right); initial potential: − 0.1 V].

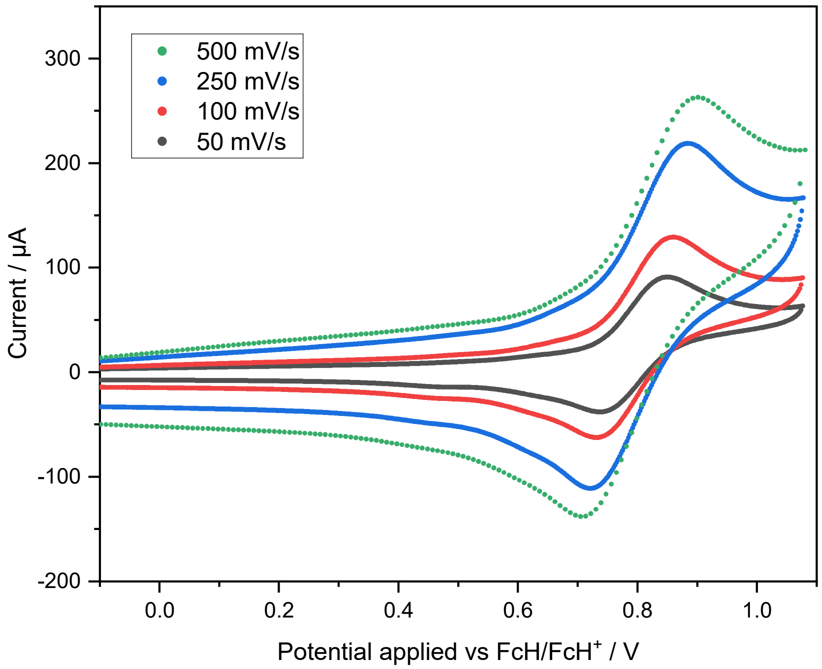

Figure S41. Cyclic voltammograms of **1S** (1 mM) in CH_2Cl_2 at room temperature, at various scan rates with [^{n}Bu$_4$N][PF$_6$] (0.1 M) as supporting electrolyte and reported against the ferrocene/ferrocenium redox couple (FcH/FcH$^+$).

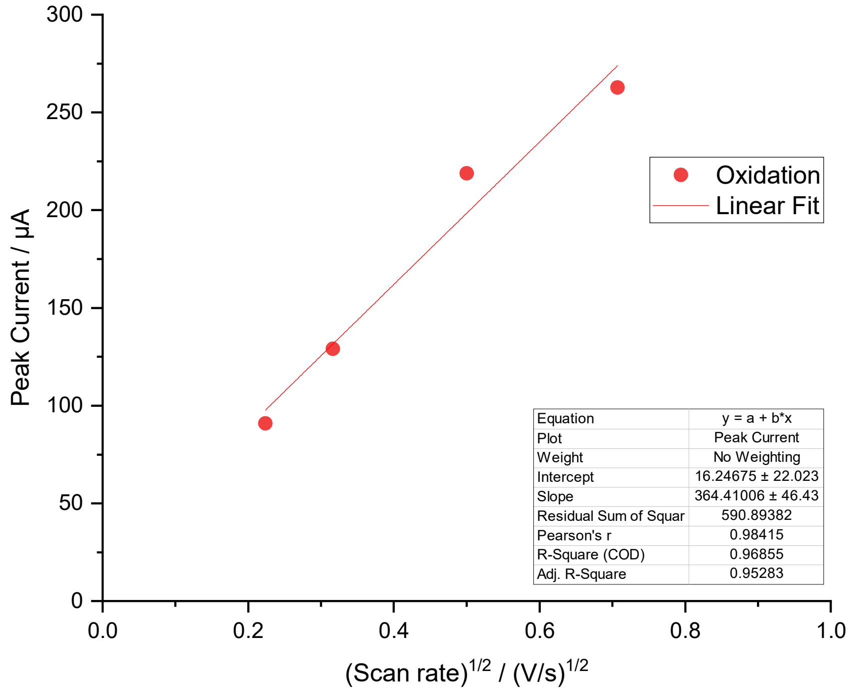

Figure S42. Plot of the oxidation peak currents of **1S** versus the square root of the scan rate.

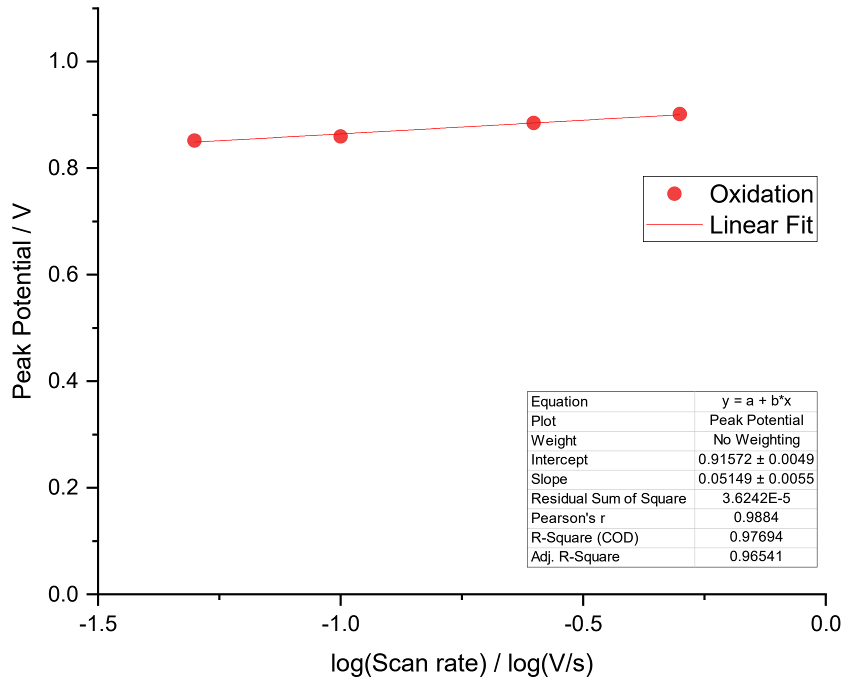

Figure S43. Plot of the oxidation peak potentials of **1S** versus the decadic logarithm of the scan rate.

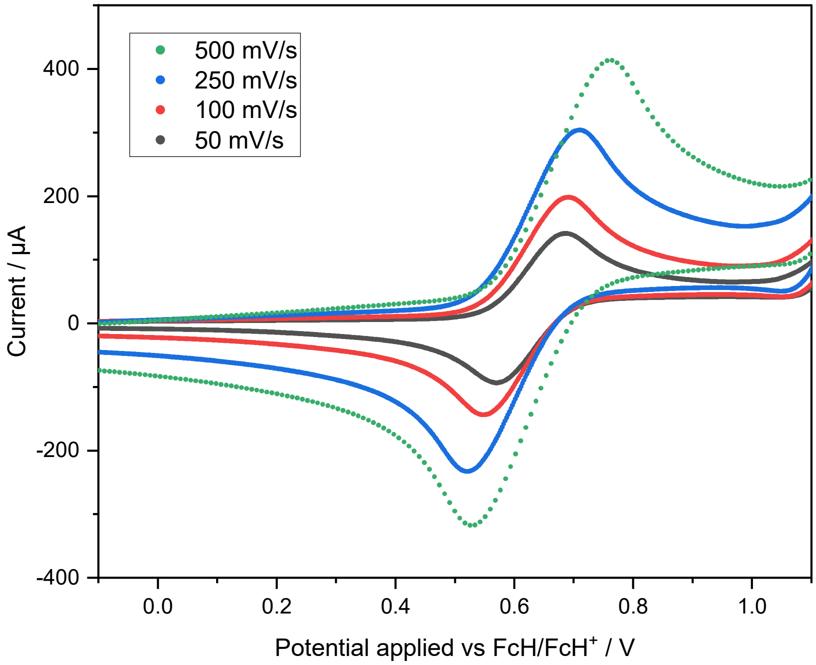

Figure S44. Cyclic voltammograms of **1Se** (1 mM) in CH_2Cl_2 at room temperature, at various scan rates with [^{n}Bu$_4$N][PF_6] (0.1 M) as supporting electrolyte and reported against the ferrocene/ferrocenium redox couple (FcH/FcH^+).

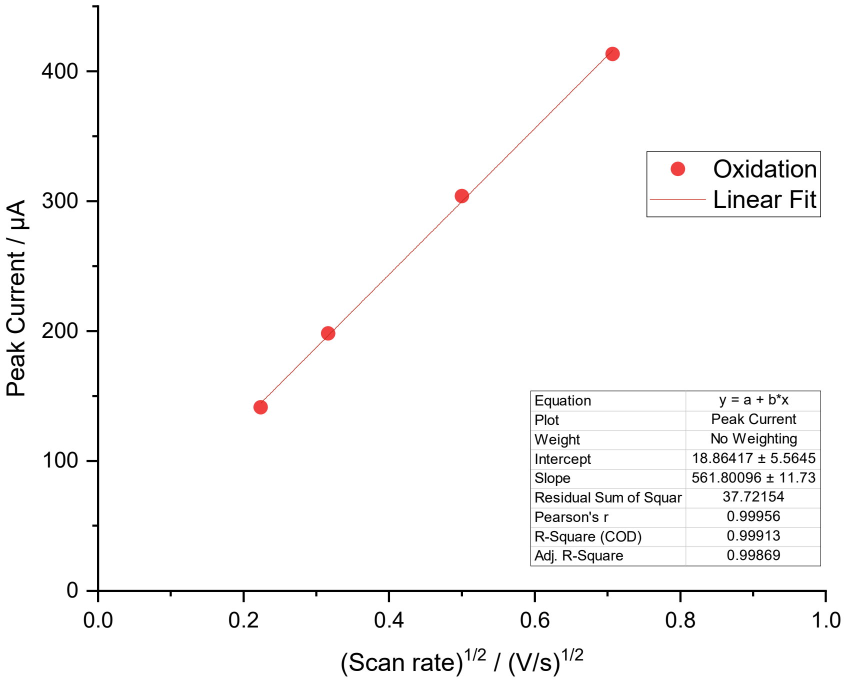

Figure S45. Plot of the oxidation peak currents of **1Se** versus the square root of the scan rate.

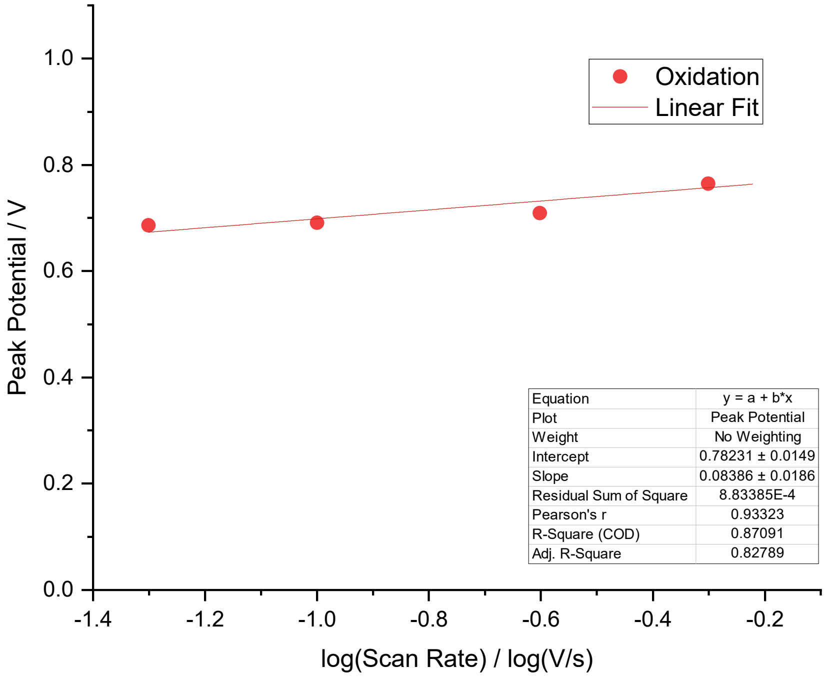

Figure S46. Plot of the oxidation peak potentials of **1Se** versus the decadic logarithm of the scan rate.

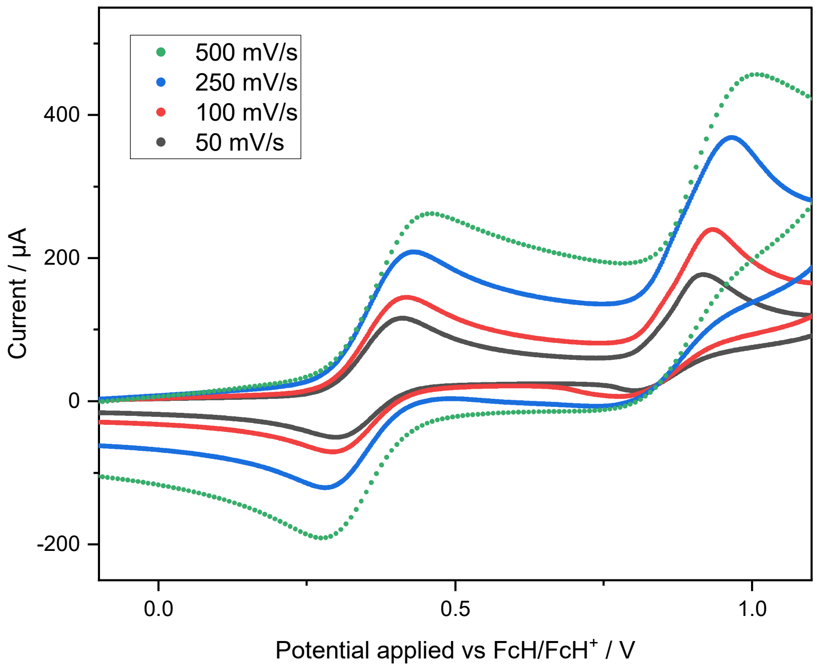

Figure S47. Cyclic voltammograms of **1Te** (1 mM) in CH_2Cl_2 at room temperature, at various scan rates with [^{n}Bu$_4$N][PF$_6$] (0.1 M) as supporting electrolyte and reported against the ferrocene/ferrocenium redox couple (FcH/FcH^+).

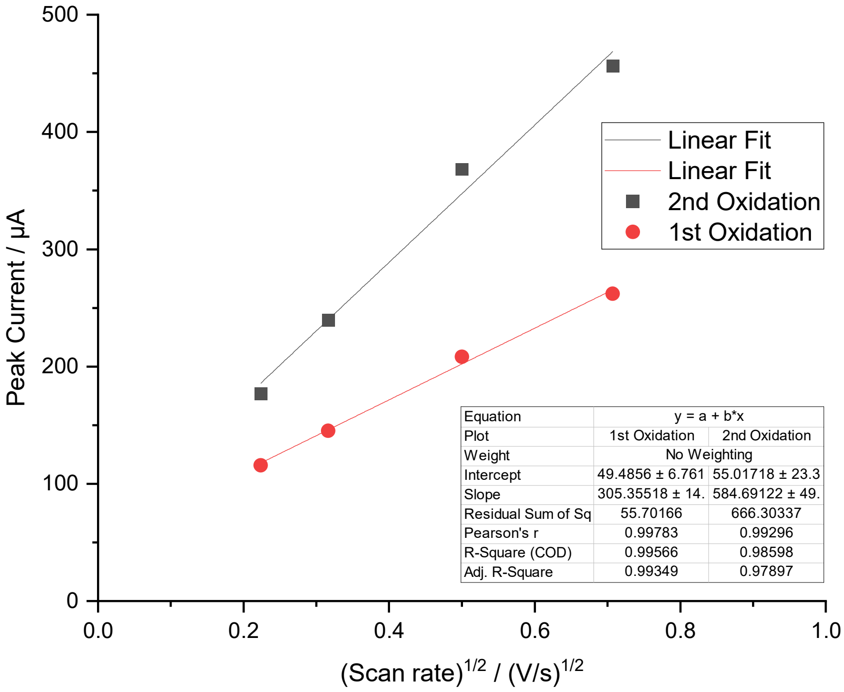

Figure S48. Plot of the oxidation peak currents of **1Te** versus the square root of the scan rate.

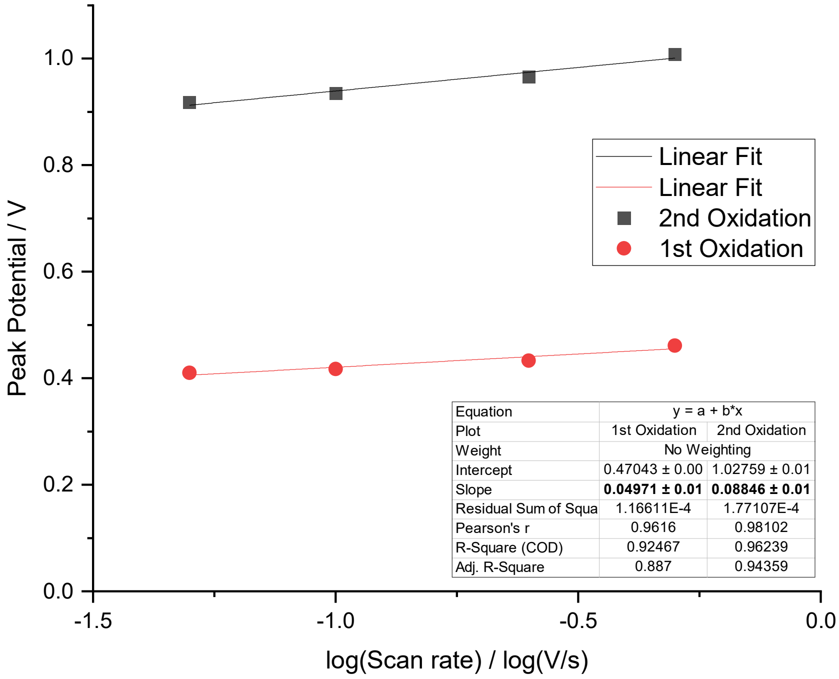

Figure S49. Plot of the oxidation peak potentials of **1Te** versus the decadic logarithm of the scan rate.

Supplementary Note 12. Spectroelectrochemical Studies

Method

For all spectroelectrochemical experiments spectroscopy grade CH_2Cl_2 (dried and degassed) was employed. All the supporting electrolytes were used as received, except for $[^{n}Bu_4N][B(C_6F_5)_4]$.[22] Spectroelectrochemical studies were performed using Microcell HC cell stand from *rhd instruments GmbH & Co.KG* combined with the TSC spectro measuring cell (see picture below). An Autolab PGSTAT 101 Metrohm potentiostat was used for bulk electrolysis. A three-electrode configuration was used with a platinum mesh (D = 0.04 mm) acting as a working electrode (WE), a stainless-steel plate (D = 6 mm) acting as counter electrode (CE) and an Ag pseudo reference electrode (pRE). The platinum mesh was cleaned in hot nitric acid, then rinsed with deionised water and HPLC grade acetone and dichloromethane. The analyte solutions (1 mM), including the supporting electrolyte (0.1 M for $[^{n}Bu_4N][PF_6]$ and $[^{n}Bu_4N][BF_4]$; 25 mM for $[^{n}Bu_4N][B(C_6F_5)_4]$), were prepared in the glovebox. The cell was assembled in the glovebox as well. Initially, an oxidation potential was applied for each analyte (details in the figure captures) and simultaneously the UV-vis region was scanned using the AvaSpec-2048 USB2 Fiber Optic spectrometer, registering the absorption (1 cm path length). Afterwards, the reduction potentials were applied while the spectra were registered, to check for reversibility and rule out decomposition. The averaged spectra were saved every 10 seconds and analysed using Spectragryph software.[23]

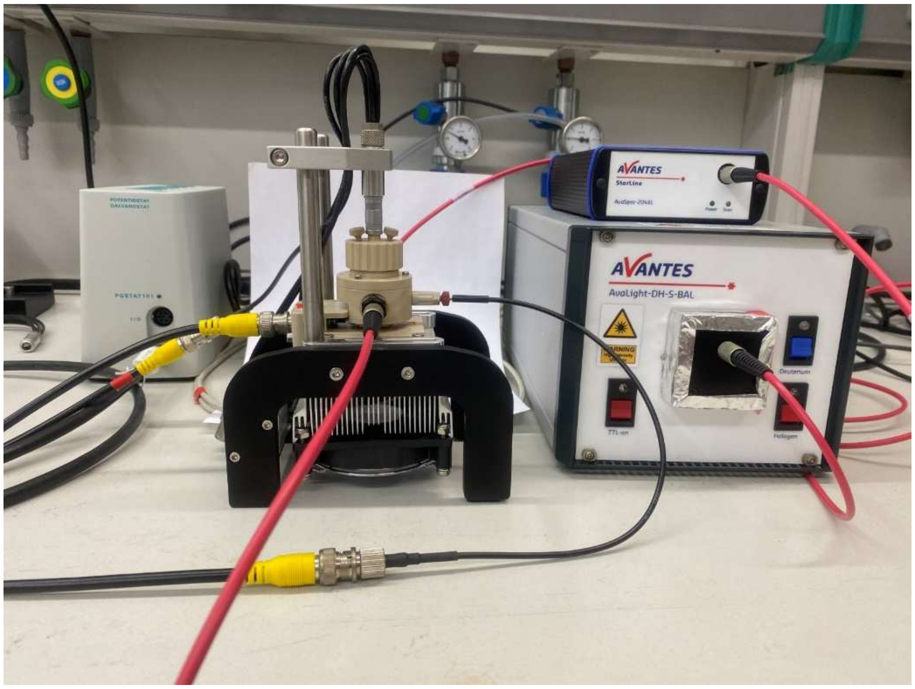

Figure S50. Spectroelectrochemistry setup: the Autolab potentiostat on the left and the Avantes spectrometer on the right.

Results.

Electrochemical bulk oxidation of **1E** (E = S, Se, Te) was studied in CH_2Cl_2 solutions using [^{n}Bu$_4$N][PF$_6$] as supporting electrolyte, while the UV-Vis absorption spectra were recorded every 10 s. Upon one-electron oxidation, an increase of the corresponding absorption bands could be observed for [**1E**][PF$_6$] (E = Se, Te) (Supplementary Figures S57 & S59). On the other hand, the bulk oxidation of **1S** in [^{n}Bu$_4$N][PF$_6$] showed only a wide absorption band maximum at 464 nm (Supplementary Figure S51), which may be a consequence of a following reaction with the PF_6^- anion. For that reason, the bulk oxidation of **1S** was studied in solutions of [^{n}Bu$_4$N][BF$_4$] as well as [^{n}Bu$_4$N][B(C$_6$F$_5$)$_4$] in CH_2Cl_2. The formation of the corresponding radical cation [**1S**][BF$_4$] was indicated by the increase in absorption of the band at 720 nm (Supplementary Figure S53). Shortly after (*ca*. 4 min of applied voltage) that band disappeared, leaving the band at 464 nm, which we attribute to an unidentified decomposition product. Finally, the bulk oxidation in [^{n}Bu$_4$N][B(C$_6$F$_5$)$_4$] (solution in CH_2Cl_2) revealed the bands with absorption maxima similar to the ones of the isolated [**1S**][B(C$_6$F$_5$)$_4$], with the one at 464 nm not being observed, confirming once again that the sulphur radical cation is the most reactive (Supplementary Figure S55).

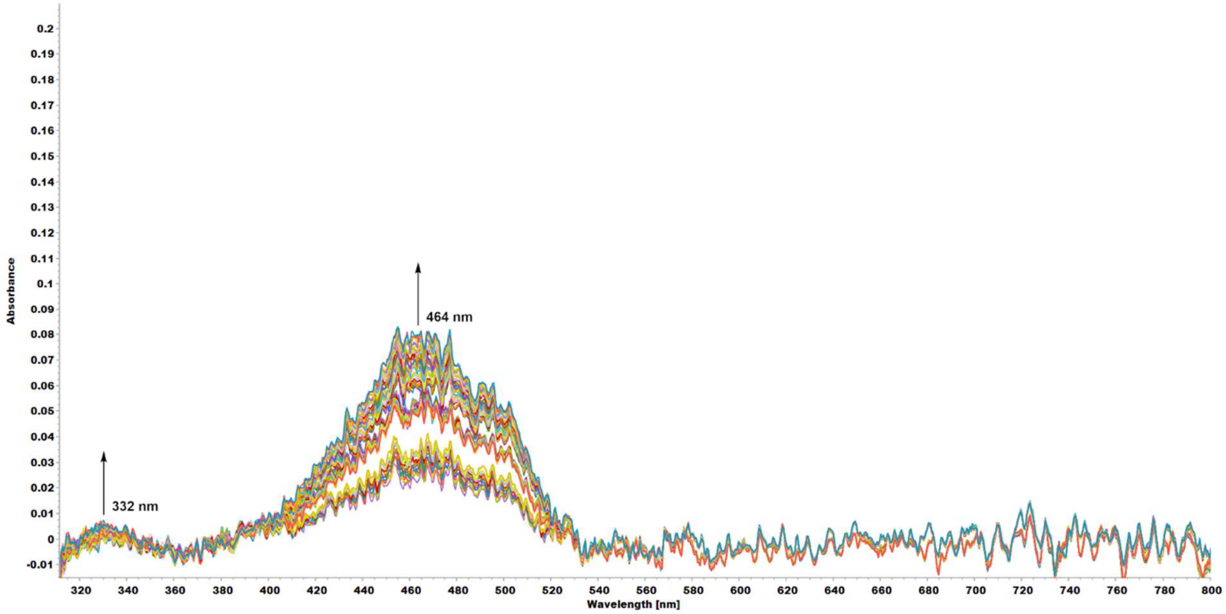

Figure S51. UV-Vis absorption spectra of **1S** at different times during bulk oxidation (applied voltage of **1.5 V** for 300 s) using [^{n}Bu$_4$N][PF$_6$] as supporting electrolyte.

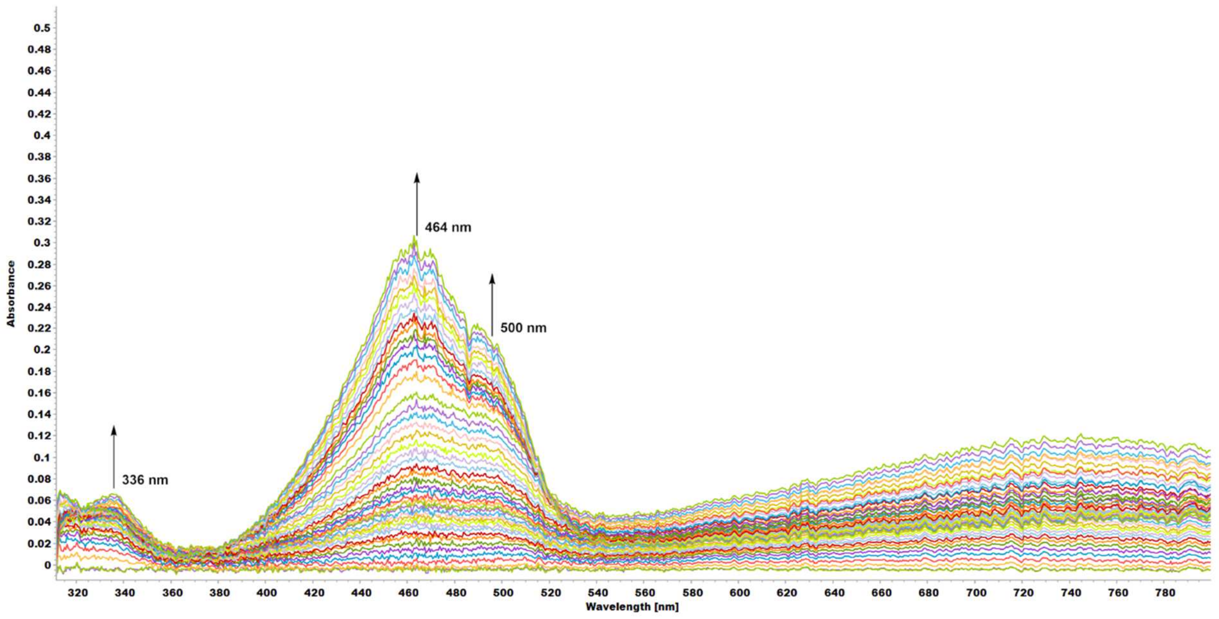

Figure S53. UV-Vis absorption spectra of **1S** at different times during bulk oxidation (applied voltage of **1.4 V** for 300 s) using [^{n}Bu$_4$N][BF$_4$] as supporting electrolyte.

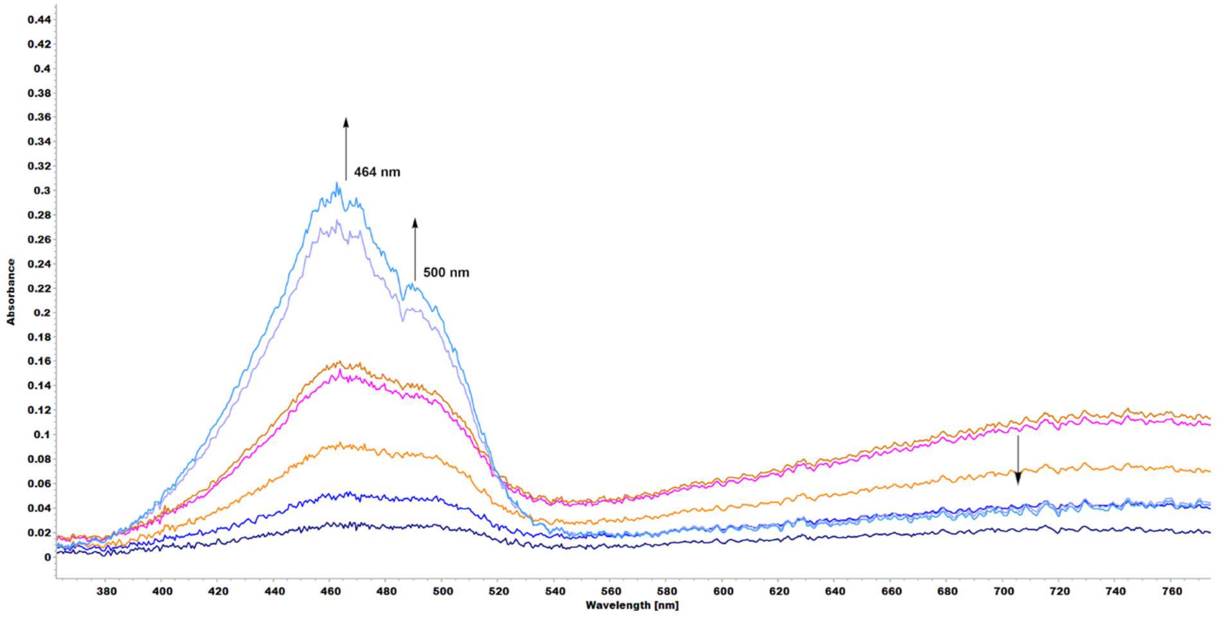

Figure S54. UV-Vis absorption spectra of **1S** at different times during bulk oxidation (applied voltage of **1.4 V**) using [^{n}Bu$_4$N][BF$_4$] as supporting electrolyte; the absorption of the band at ca. 720 nm starts decreasing after approximately 4 min of bulk oxidation.

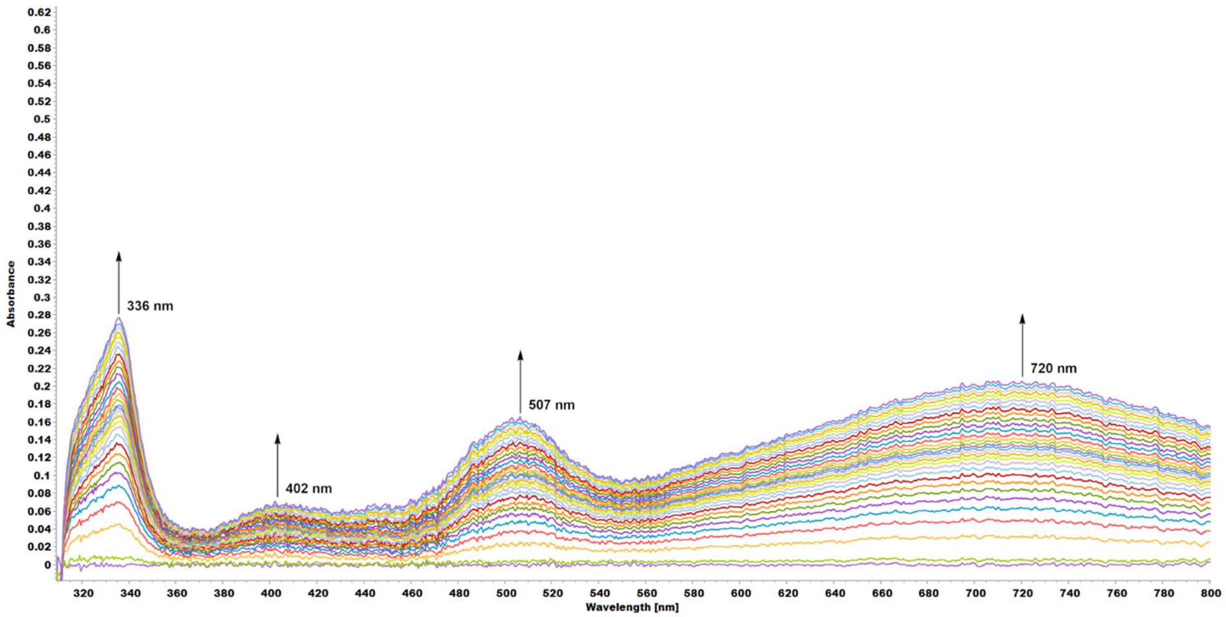

Figure S55. UV-Vis absorption spectra of **1S** at different times during bulk oxidation (applied voltage of **1.5 V** for 300 s) using [^{n}Bu$_4$N][B(C$_6$F$_5$)$_4$] as supporting electrolyte.

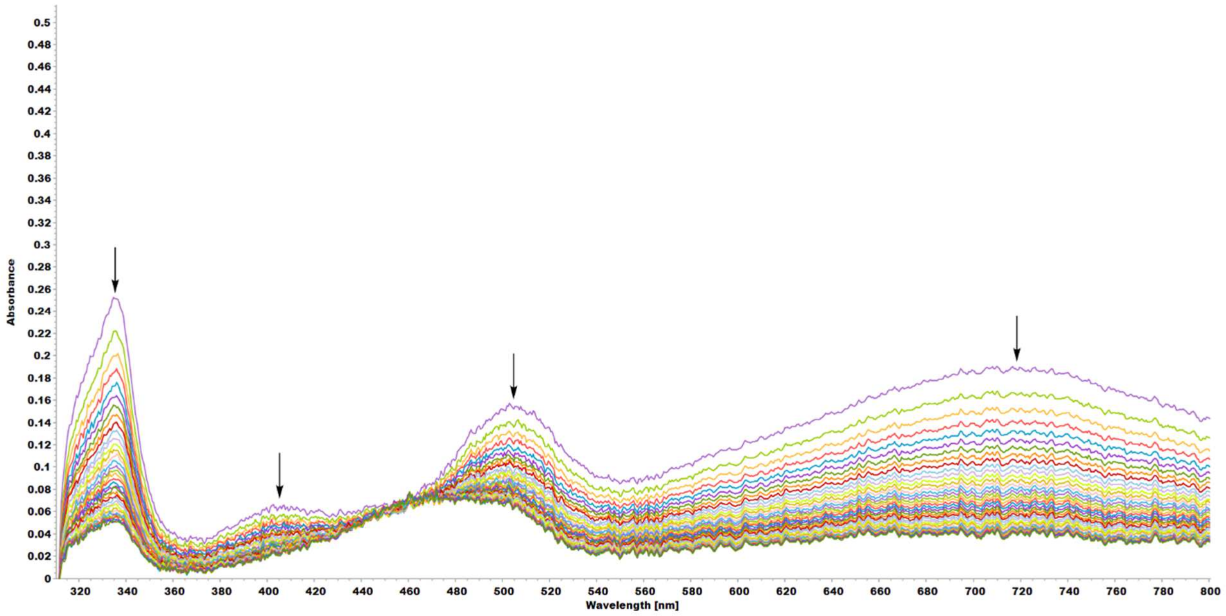

Figure S56. UV-Vis absorption spectra of **1S** at different times during bulk reduction (applied voltage of **1.1 V** for 300 s) using [^{n}Bu$_4$N][B(C$_6$F$_5$)$_4$] as supporting electrolyte.

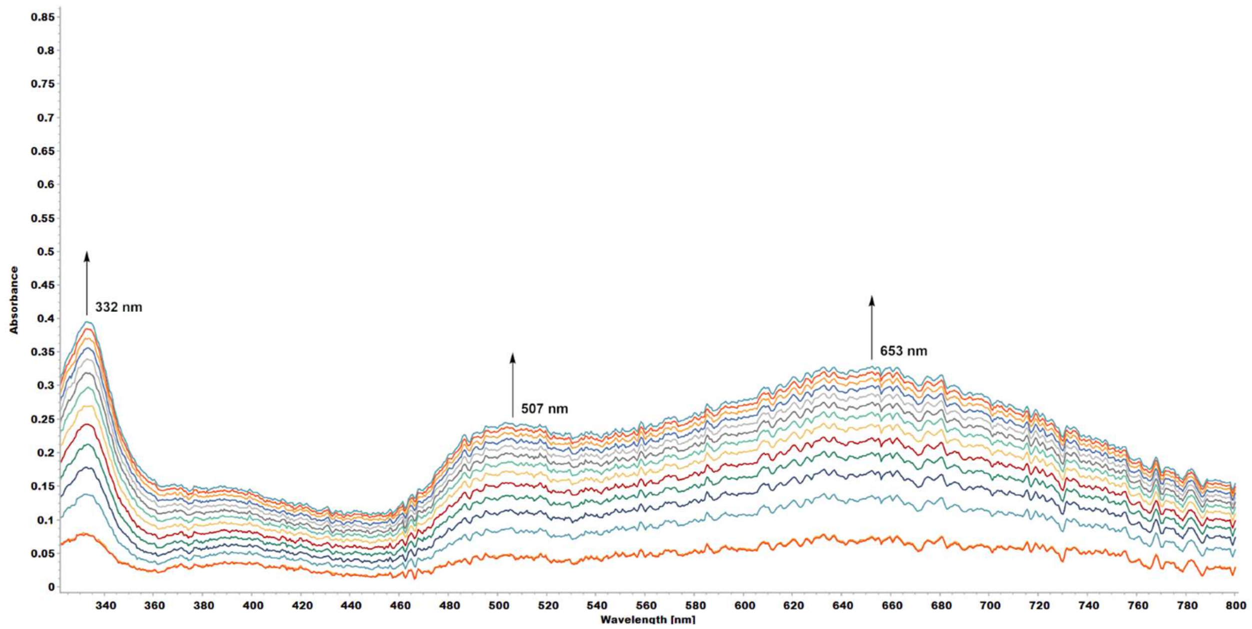

Figure S57. UV-Vis absorption spectra of **1Se** at different times during bulk oxidation (applied voltage of **1.0 V** for 120 s) using [^{n}Bu$_4$N][PF$_6$] as supporting electrolyte.

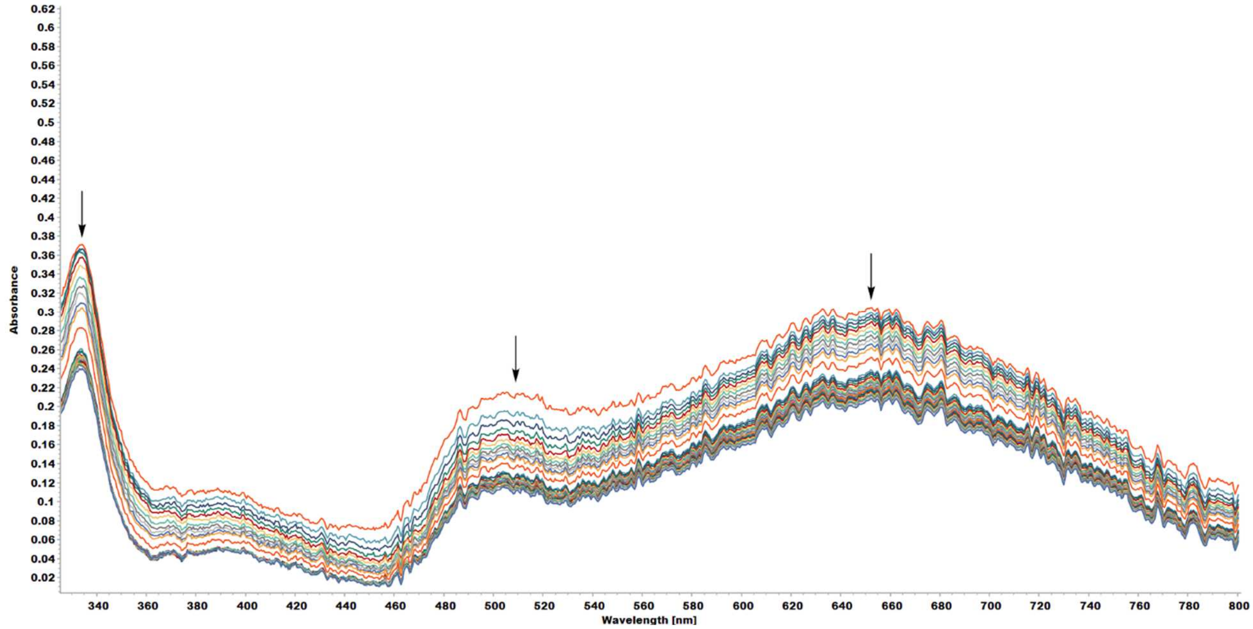

Figure S58. UV-Vis absorption spectra of **1Se** at different times during bulk reduction (applied voltage of **0.5 V** for 300 s) using [^{n}Bu$_4$N][PF$_6$] as supporting electrolyte.

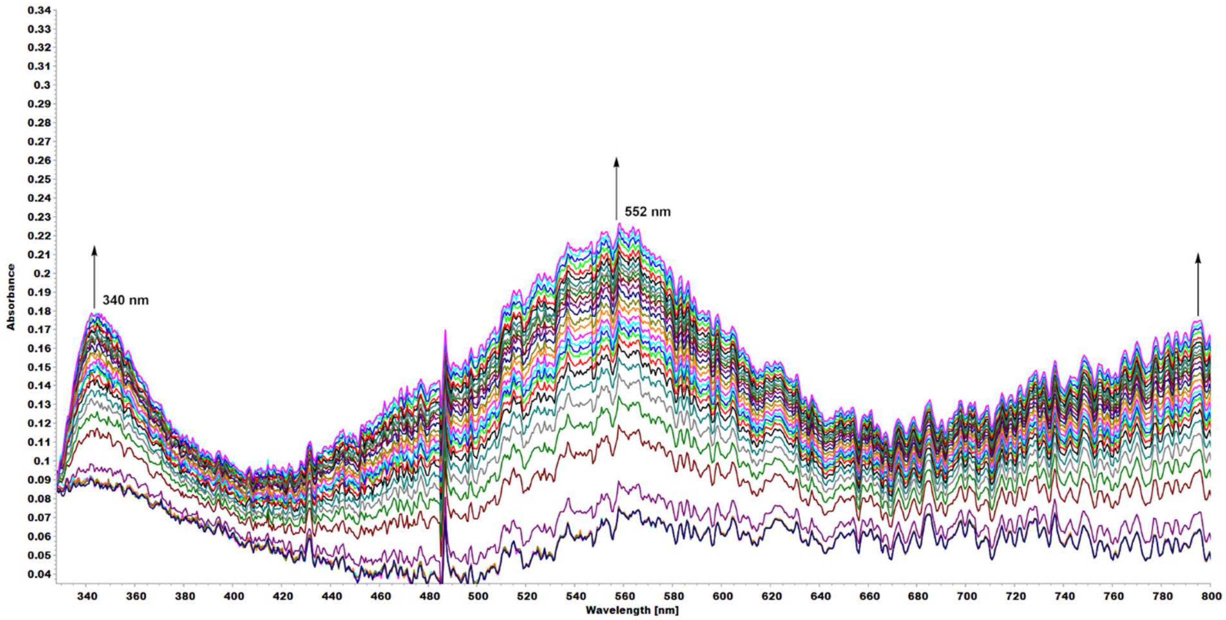

Figure S59. UV-Vis absorption spectra of **1Te** at different times during bulk oxidation (applied voltage of **0.84 V** for 240 s) using [^{n}Bu$_4$N][PF$_6$] as supporting electrolyte.

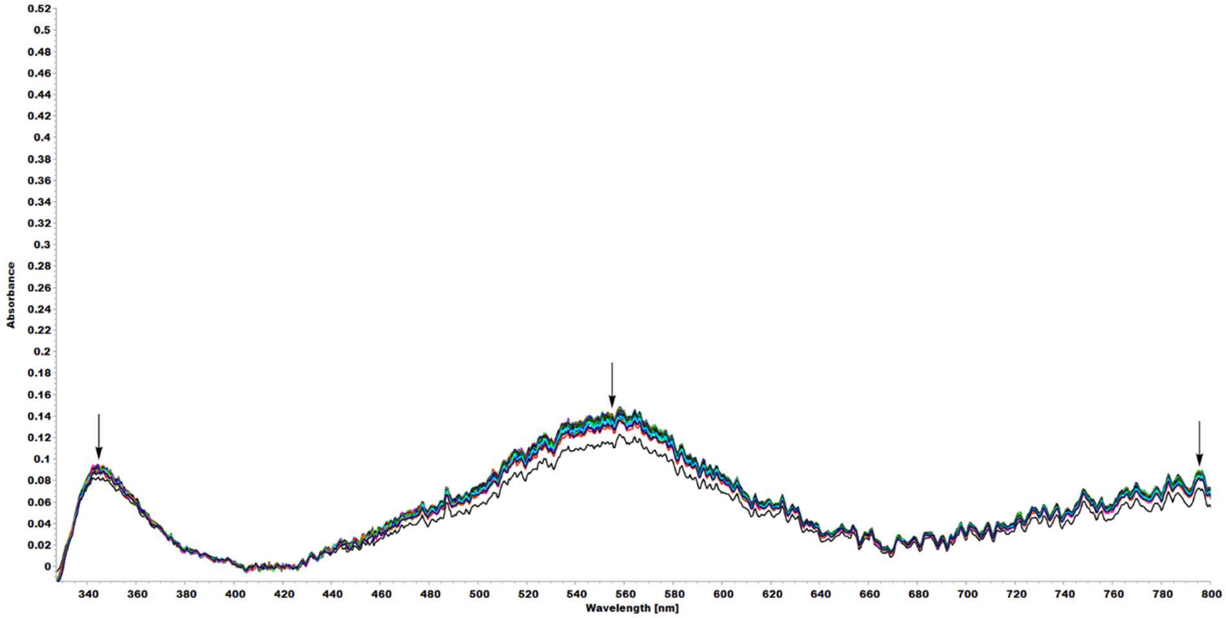

Figure S60. UV-Vis absorption spectra of **1Te** at different times during bulk reduction (applied voltage of **0.54 V** for 640 s) using [^{n}Bu$_4$N][PF$_6$] as supporting electrolyte.

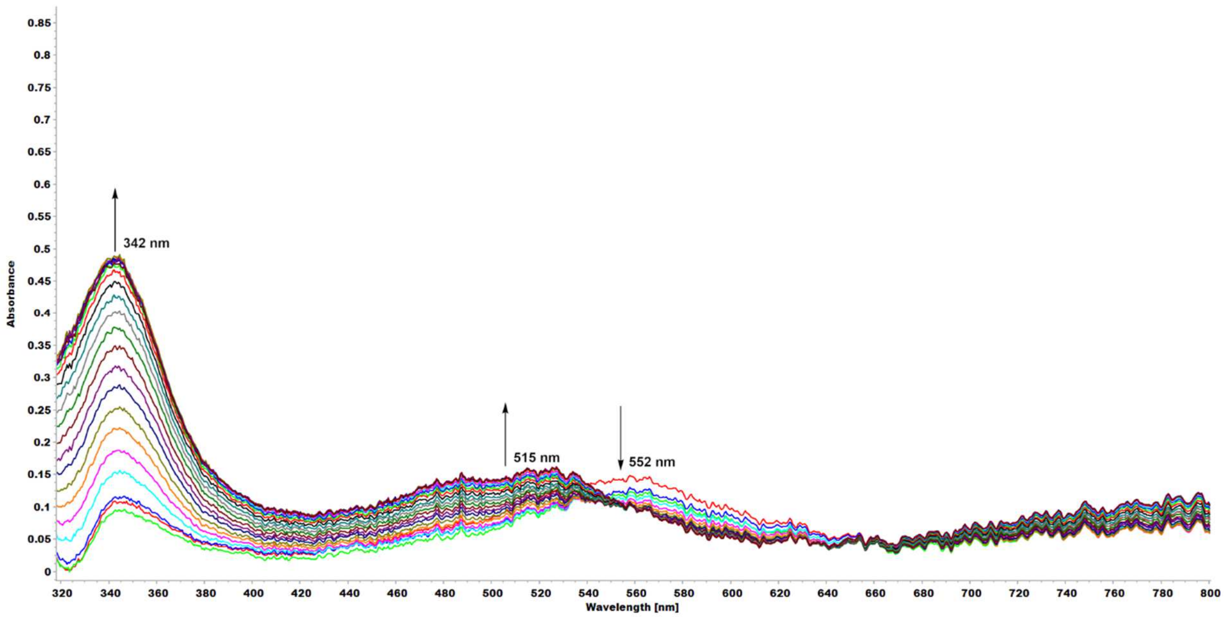

Figure S61. UV-Vis absorption spectra of **1Te** at different times during bulk oxidation (applied voltage of **1.36 V** for 240 s) using [^{n}Bu$_4$N][PF$_6$] as supporting electrolyte.

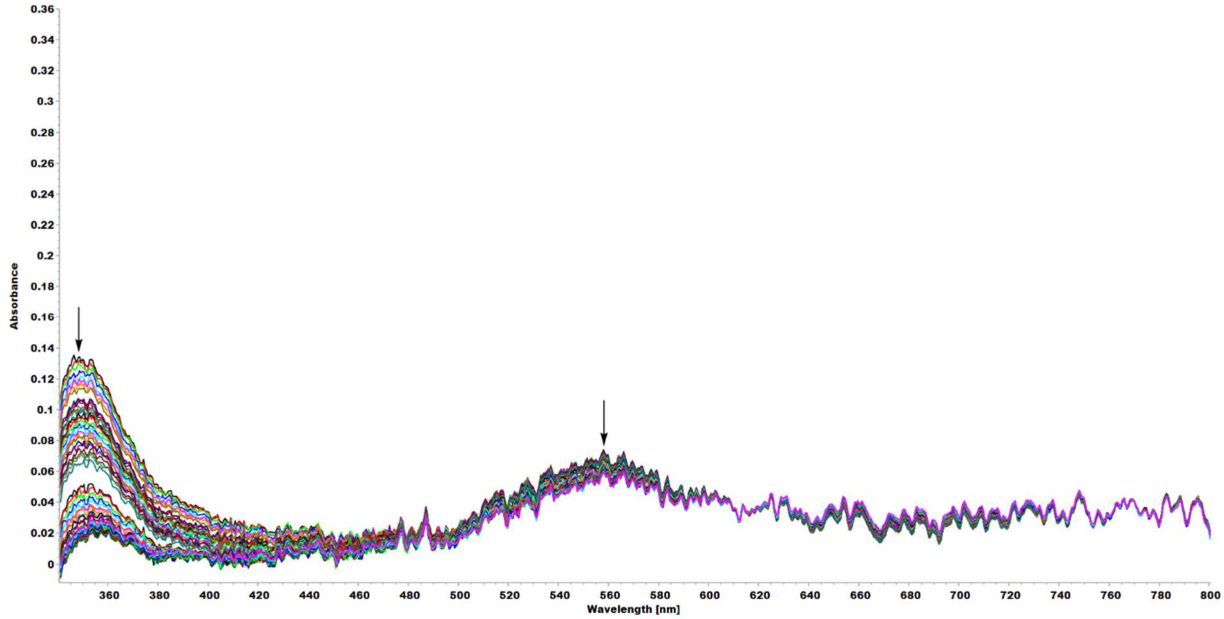

Figure S62. UV-Vis absorption spectra of **1Te** at different times during bulk reduction (applied voltage of **1.04 V** for 600 s) using [^{n}Bu$_4$N][PF$_6$] as supporting electrolyte.

Supplementary Note 13. X-Ray Diffraction Studies

Intensity data of **1S**, **1Se**, **1Te**, [**1Se**][B(C_6F_5)$_4$], [**1Te**][B(C_6F_5)$_4$]·CH_2Cl_2, [**1Te**][SbF_6], M^SFluindPhTeF_2, [M^SFluindPhTeF_2]$_2$·K[B(C_6F_5)$_4$], [M^SFluindTeOH][$B_{12}Cl_{12}$]$_{0.5}$ was collected on a Bruker Venture D8 diffractometer at 100 K with graphite-monochromatic Mo-Kα (0.7107 Å) radiation. All structures were solved by direct methods and refined based on F^2 by use of the SHELX program package as implemented in WinGX[24–26] or OLEX2.[27] All non-hydrogen atoms were refined using anisotropic displacement parameters. Hydrogen atoms attached to carbon atoms were included in geometrically calculated positions using a riding model. Crystal and refinement data are collected in Supplementary Tables S70–S72. Figures were created using Diamond.[28] Crystallographic data for the structural analyses have been deposited with the Cambridge Crystallographic Data Centre. Copies of this information may be obtained free of charge from The Director, CCDC, 12 Union Road, Cambridge CB2 1EZ, UK (Fax: +44-1223-336033; e-mail: deposit@ccdc.cam.ac.uk or http://www.ccdc.cam.ac.uk).

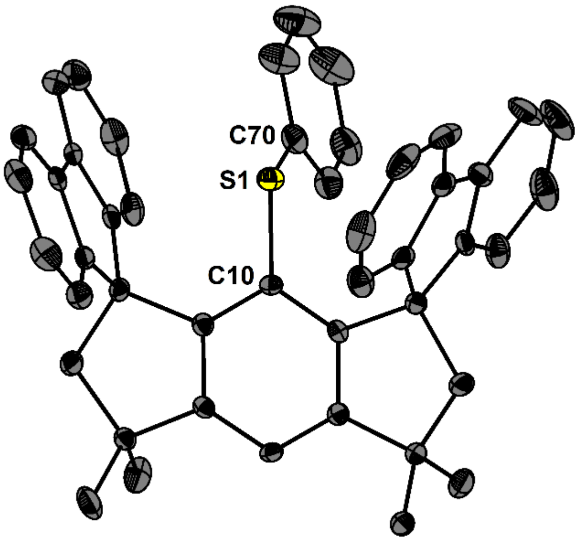

Figure S63. Molecular structure of **1S** showing 50% probability ellipsoids and the essential atomic numbering. Hydrogen atoms are omitted.

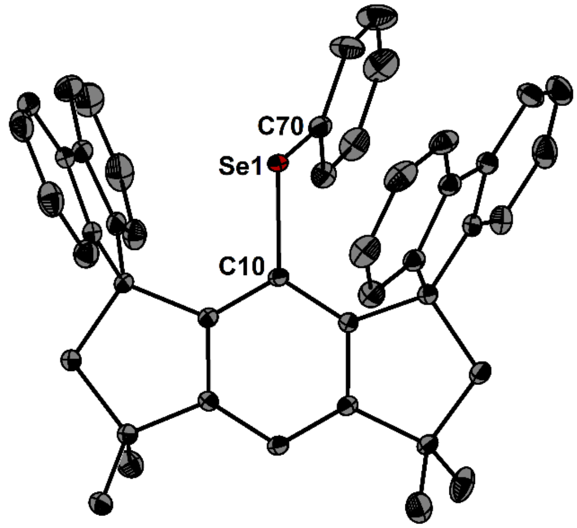

Figure S64. Molecular structure of **1Se** showing 50% probability ellipsoids and the essential atomic numbering. Hydrogen atoms are omitted.

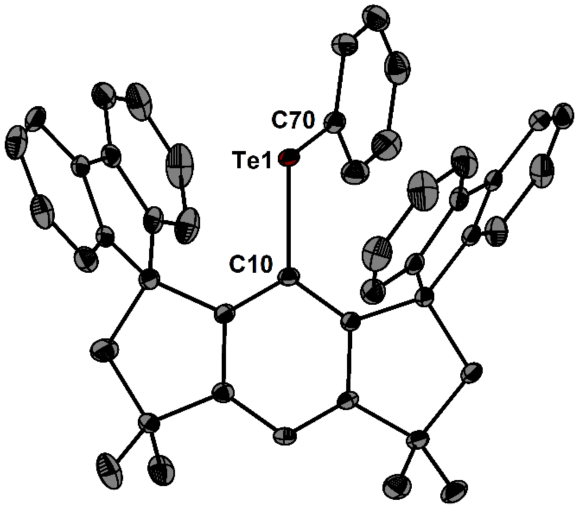

Figure S65. Molecular structure of **1Te** showing 50% probability ellipsoids and the essential atomic numbering. Hydrogen atoms are omitted.

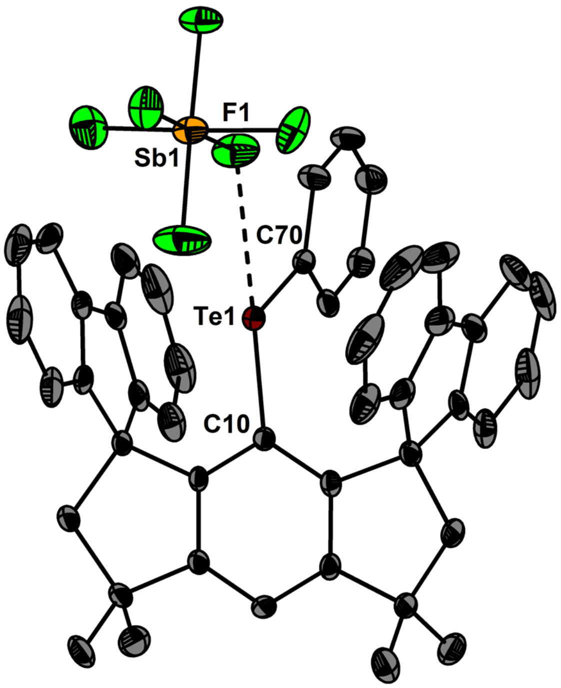

Figure S66. Molecular structure of [**1Te**][SbF_6] showing 50% probability ellipsoids and the essential atomic numbering. Hydrogen atoms are omitted.

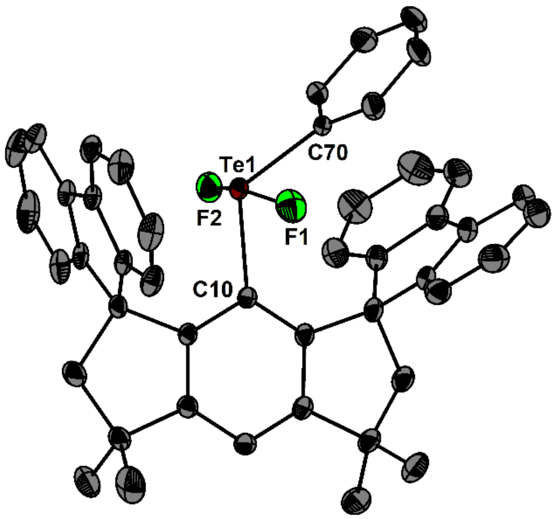

Figure S67. Molecular structure of M^SFluindPhTeF_2 showing 50% probability ellipsoids and the essential atomic numbering. Hydrogen atoms and solvent molecules are omitted.

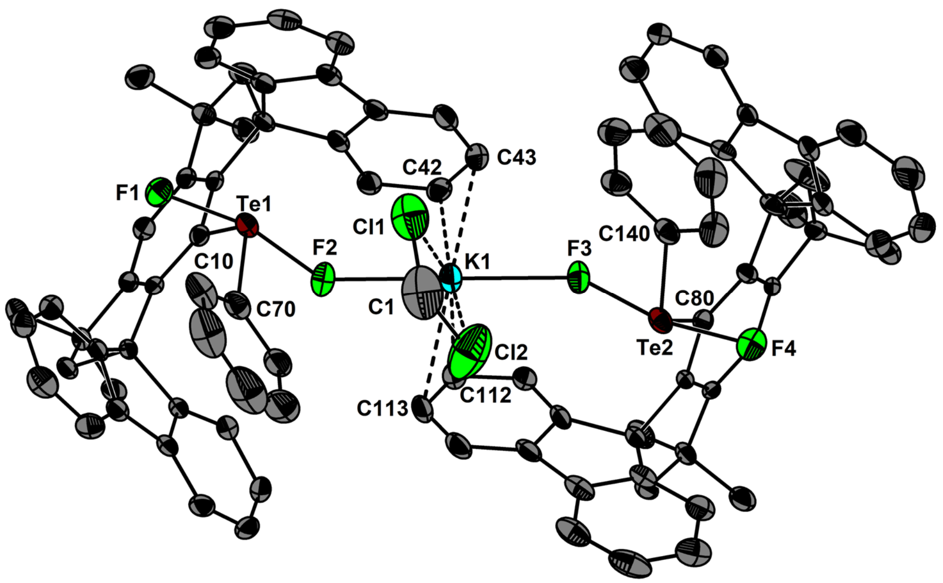

Figure S68. Molecular structure of [M^{S}FluindPhTeF$_2$]$_2$·K[B(C_6F_5)$_4$] showing 50% probability ellipsoids and the essential atomic numbering. Hydrogen atoms, solvent molecules and counter-ions are omitted. A mixture of compounds was obtained by combining M^{S}FluindPhTeF$_2$ with K[B(C_6F_5)$_4$] in CH_2Cl_2 at room temperature. Crystals were obtained by layering ^{n}hexane on top of the filtered solution.

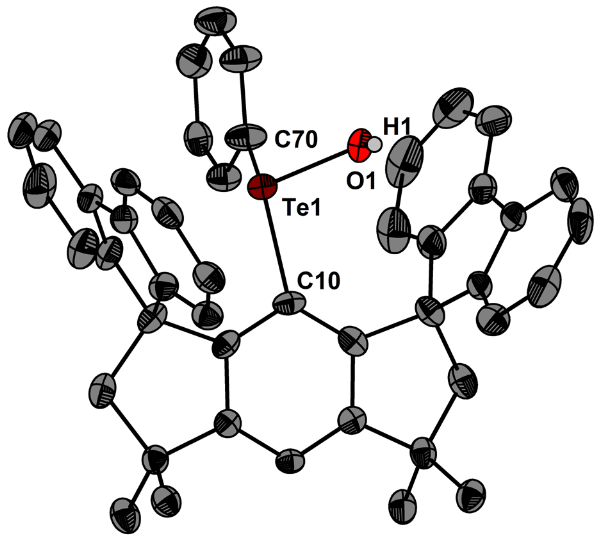

Figure S69. Molecular structure of [M^{S}FluindPhTeOH][$B_{12}Cl_{12}$]$_{0.5}$ showing 50% probability ellipsoids and the essential atomic numbering. Carbon-bound hydrogen atoms and the counter-ion are omitted. A mixture of compounds was obtained by combining **1Te** with Cs[$B_{12}Cl_{12}$] and XeF_2 in CH_2Cl_2 at −78°C to room temperature. Crystals were obtained by layering ^{n}hexane on top of the filtered solution.

Table S70. Crystal data and structure refinement of **1S**, **1Se** and **1Te**.

	**1S**	**1Se**	**1Te**·0.5 toluene
Formula	$C_{46}H_{38}S$	$C_{46}H_{38}Se$	$C_{49.5}H_{42}Te$
Formula weight, g mol^{-1}	622.82	669.72	764.43
Crystal system	monoclinic	monoclinic	monoclinic
Crystal size, mm	0.30 × 0.20 × 0.10	0.21 × 0.17 × 0.11	0.57 × 0.43 × 0.34
Space group	$P2_1/n$	$P2_1/n$	$P2_1/n$
a, Å	10.488(3)	10.5031(3)	11.5773(6)
b, Å	16.261(4)	16.3544(4)	16.6056(7)
c, Å	19.816(4)	19.8302(5)	19.3064(11)
α, °	90	90	90
β, °	100.565(9)	100.4070(10)	101.595(2)
γ, °	90	90	90
V, Å^3	3322.2(13)	3350.24(15)	3635.9(3)
Z	4	4	4
ρ_{calcd}, Mg m^{-3}	1.245	1.328	1.396
μ (Mo *K*α), mm^{-1}	0.131	1.154	0.853
F(000)	1320	1392	1564
θ range, °	1.97 to 25.01	2.33 to 31.51	2.26 to 31.00
Index ranges	$-12 \le h \le 12$	$-15 \le h \le 15$	$-16 \le h \le 16$
	$-19 \le k \le 18$	$-24 \le k \le 24$	$-18 \le k \le 24$
	$-23 \le l \le 23$	$-29 \le l \le 29$	$-21 \le l \le 27$
No. of reflns collected	30397	187915	43763
Completeness to θ_{max}	99.9%	99.9%	99.7%
No. indep. Reflns	5895	11160	11507
No. obsd reflns with ($I>2\sigma(I)$)	4892	9965	9672
No. refined params	429	428	429
GooF (F^2)	1.074	1.036	1.042
R_1 (F) ($I > 2\sigma(I)$)	0.0621	0.0273	0.0354
wR_2 (F^2) (all data)	0.1661	0.0707	0.0869
Largest diff peak/hole, e Å^{-3}	0.72 / –0.80	0.47 / –0.36	1.68 / –1.42
CCDC numbers	2419127	2419128	2419129

Table S71. Crystal data and structure refinement of [**1Se**][B(C_6F_5)$_4$], [**1Te**][B(C_6F_5)$_4$] and [**1Te**][SbF_6].

	[**1Se**][B(C_6F_5)$_4$] ·CH_2Cl_2	[**1Te**][B(C_6F_5)$_4$] ·CH_2Cl_2	[**1Te**][SbF_6]
Formula	$C_{71}H_{40}BCl_2F_{20}Se$	$C_{71}H_{43}BCl_2F_{20}Te$	$C_{46}H_{38}F_6SbTe$
Formula weight, g mol^{-1}	1433.70	1485.36	954.11
Crystal system	monoclinic	monoclinic	monoclinic
Crystal size, mm	0.32 × 0.25 × 0.18	0.25 × 0.20 × 0.08	0.31 × 0.12 × 0.07
Space group	$P2_1/c$	$P2_1/n$	$P2_1/c$
a, Å	10.4093(6)	10.5576(12)	11.1411(8)
b, Å	23.4103(16)	35.521(3)	16.9199(14)
c, Å	25.9992(18)	15.5538(11)	20.1869(14)
α, °	90	90	90
β, °	90.115(2)	90.060(4)	99.432(3)
γ, °	90	90	90
V, Å^3	6335.6(7)	5833.0(9)	3753.9(5)
Z	4	4	4
ρ_{calcd}, Mg m^{-3}	1.503	1.688	1.688
μ (Mo $K\alpha$), mm^{-1}	0.779	0.713	1.560
F(000)	2876	2948	1884
θ range, °	2.50 to 31.03	2.24 to 36.38	2.21 to 27.50
Index ranges	$-15 \le h \le 15$	$-17 \le h \le 17$	$-14 \le h \le 14$
	$-29 \le k \le 33$	$-58 \le k \le 59$	$-21 \le k \le 21$
	$-35 \le l \le 37$	$-25 \le l \le 25$	$-26 \le l \le 26$
No. of reflns collected	177488	170781	122817
Completeness to θ_{max}	99.7%	99.9%	99.9%
No. indep. Reflns	20138	28328	8623
No. obsd reflns with ($I>2\sigma(I)$)	16340	23368	7773
No. refined params	887	860	491
GooF (F^2)	1.044	1.033	1.207
R_1 (F) ($I > 2\sigma(I)$)	0.0523	0.0381	0.0417
wR_2 (F^2) (all data)	0.1574	0.1031	0.0949
Largest diff peak/hole, e Å^{-3}	1.57 / −0.85	1.58 / −1.17	1.54 / −1.12
CCDC numbers	2419130	2419131	2419132

Table S72. Crystal data and structure refinement of $M^SFluindPhTeF_2$, $[M^SFluindPhTeF_2]_2 \cdot K[B(C_6F_5)_4]$ and $[M^SFluindPhTeOH][B_{12}Cl_{12}]_{0.5}$.

	$M^SFluindPhTeF_2\cdot$ heptane	$[M^SFluindPhTeF_2]_2\cdot$ $K[B(C_6F_5)_4]\cdot CH_2Cl_2$	$[M^SFluindPhTeOH]$ $[B_{12}Cl_{12}]_{0.5}$
Formula	$C_{53}H_{54}F_2Te$	$C_{117}H_{78}BCl_2F_{24}KTe_2$	$C_{46}H_{39}B_6Cl_6OTe$
Formula weight, g mol^{-1}	856.56	2315.80	1012.93
Crystal system	monoclinic	monoclinic	monoclinic
Crystal size, mm	0.11 × 0.10 × 0.08	0.08 × 0.05 × 0.03	0.16 × 0.13 × 0.11
Space group	$P2_1/n$	$P2_1/c$	$P2_1/c$
a, Å	13.4502(15)	20.8910(6)	10.2739(16)
b, Å	14.2357(13)	27.5986(7)	25.429(4)
c, Å	20.890(2)	17.3844(4)	16.781(3)
α, °	90	90	90
β, °	100.045(4)	104.0930(10)	95.289(5)
γ, °	90	90	90
V, Å^3	3938.5(7)	9721.5(4)	4365.3(13)
Z	4	4	4
ρ_{calcd}, Mg m^{-3}	1.490	1.582	1.541
μ (Mo $K\alpha$), mm^{-1}	0.803	0.797	1.086
F(000)	1788	4632	2028
2θ range, °	2.10 to 36.34	1.91 to 25.00	1.99 to 25.00
Index ranges	$-22 \le h \le 22$	$-24 \le h \le 24$	$-12 \le h \le 12$
	$-23 \le k \le 23$	$-32 \le k \le 32$	$-31 \le k \le 31$
	$-32 \le l \le 34$	$-20 \le l \le 20$	$-20 \le l \le 20$
No. of reflns collected	159971	173568	192712
Completeness to θ_{max}	99.8%	99.9%	99.9%
No. indep. Reflns	19096	17120	8590
No. obsd reflns with ($I>2\sigma(I)$)	15558	13754	7735
No. refined params	510	1332	565
GooF (F^2)	1.080	1.055	1.097
R_1 (F) ($I > 2\sigma(I)$)	0.0552	0.0445	0.0436
wR_2 (F^2) (all data)	0.1318	0.1220	0.1010
Largest diff peak/hole, e Å^{-3}	7.27 / −1.86	2.67 / −0.67	1.03 / −1.08
CCDC numbers	2419133	2419134	2419135

Supplementary Note 14. Electron Paramagnetic Resonance

Table S73. Parameters used for simulation of X- and Q-band EPR spectra.

	Parameters	X-band	Q-band
**[1S]** $^{\bullet+}$	g	[2.00755, 2.00755, 2.00275]	[2.00693, 2.00476, 2.00198]
	A(H) [MHz] (2 equiv)	-	[23, 21, 5]
	line width [G]	[2.9 1.7]	5
	HStrain [MHz]	-	[11, 42, 13]
**[1Se]** $^{\bullet+}$	g	[2.05625, 2.02802, 1.99944]	[2.05450, 2.02713, 1.99812]
	A(Se) [MHz]	[−90, −120, 775]	[−90, −130, 772]
	HStrain [MHz]	[40, 23, 20]	[58, 35, 24]
	gStrain	[0, 0.003, 0.005]	-
**[1Te]** $^{\bullet+}$	g	[2.14389, 2.04398, 1.97201]	[2.14351, 2.04322, 1.97135]
	A(Se) [MHz]	[690, 950, −2190]	[690, 950, −2185]
	HStrain [MHz]	[60, 35, 40]	[120, 75, 35]
	AStrain [MHz]	[0, 80, 80]	[0, 0, 70]

Supplementary References

1. Olaru, M., Mebs, S. & Beckmann, J. Cationic Carbene Analogues: Donor-Free Phosphenium and Arsenium Ions. *Angew. Chem. Int. Ed.* **60,** 19133–19138; 10.1002/anie.202107975 (2021).
2. Romanato, P., Duttwyler, S., Linden, A., Baldridge, K. K. & Siegel, J. S. Competition between π-arene and lone-pair halogen coordination of silylium ions? *J. Am. Chem. Soc.* **133,** 11844–11846; 10.1021/ja2040392 (2011).
3. Fulmer, G. R. *et al.* NMR Chemical Shifts of Trace Impurities: Common Laboratory Solvents, Organics, and Gases in Deuterated Solvents Relevant to the Organometallic Chemist. *Organometallics* **29,** 2176–2179; 10.1021/om100106e (2010).
4. Stoll, S. & Schweiger, A. EasySpin, a comprehensive software package for spectral simulation and analysis in EPR. *J. Magn. Reson.* **178,** 42–55; 10.1016/j.jmr.2005.08.013 (2006).
5. Neese, F. The ORCA program system. *WIREs Comput. Mol. Sci.* **2,** 73–78; 10.1002/wcms.81 (2012).
6. Neese, F., Wennmohs, F., Becker, U. & Riplinger, C. The ORCA quantum chemistry program package. *J. Chem. Phys.* **152**; 10.1063/5.0004608 (2020).
7. Adamo, C. & Barone, V. Toward reliable density functional methods without adjustable parameters: The PBE0 model. *J. Chem. Phys.* **110,** 6158–6170; 10.1063/1.478522 (1999).
8. Grimme, S., Antony, J., Ehrlich, S. & Krieg, H. A consistent and accurate ab initio parametrization of density functional dispersion correction (DFT-D) for the 94 elements H-Pu. *J. Chem. Phys.* **132**; 10.1063/1.3382344 (2010).
9. Grimme, S., Ehrlich, S. & Goerigk, L. Effect of the Damping Function in Dispersion Corrected Density Functional Theory. *J. Comput. Chem.* **32,** 1456–1465; 10.1002/jcc.21759 (2011).
10. van Lenthe, E., van Leeuwen, R., Baerends, E. J. & Snijders, J. G. Relativistic regular two-component Hamiltonians. *Int. J. Quantum Chem.* **57,** 281–293; 10.1002/(Sici)1097-461x(1996)57:3<281::Aid-Qua2>3.0.Co;2-U (1996).
11. van Wüllen, C. Molecular density functional calculations in the regular relativistic approximation: Method, application to coinage metal diatomics, hydrides, fluorides and chlorides, and comparison with first-order relativistic calculations. *J. Chem. Phys.* **109,** 392–399; 10.1063/1.476576 (1998).
12. Weigend, F. & Ahlrichs, R. Balanced basis sets of split valence, triple zeta valence and quadruple zeta valence quality for H to Rn: Design and assessment of accuracy. *Phys. Chem. Chem. Phys.* **7,** 3297–3305; 10.1039/b508541a (2005).
13. Pantazis, D. A., Chen, X. Y., Landis, C. R. & Neese, F. All-electron scalar relativistic basis sets for third-row transition metal atoms. *J. Chem. Theory Comput.* **4,** 908–919; 10.1021/ct800047t (2008).

14. Neese, F., Wennmohs, F., Hansen, A. & Becker, U. Efficient, approximate and parallel Hartree-Fock and hybrid DFT calculations. A 'chain-of-spheres' algorithm for the Hartree-Fock exchange. *Chem. Phys.* **356,** 98–109; 10.1016/j.chemphys.2008.10.036 (2009).

15. Cossi, M., Rega, N., Scalmani, G. & Barone, V. Energies, structures, and electronic properties of molecules in solution with the C-PCM solvation model. *J. Comput. Chem.* **24,** 669–681; 10.1002/jcc.10189 (2003).

16. Bader, R. F. W. *Atoms in molecules. A quantum theory* (Clarendon Press, Oxford, 1990).

17. Keith, T. A. *AIMAll* (T. K. Gristmill Software, 2015).

18. Lefebvre, C. *et al.* Accurately extracting the signature of intermolecular interactions present in the NCI plot of the reduced density gradient versus electron density. *Phys. Chem. Chem. Phys.* **19,** 17928–17936; 10.1039/C7CP02110K (2017).

19. Lu, T. & Chen, Q. Independent gradient model based on Hirshfeld partition: A new method for visual study of interactions in chemical systems. *J. Comput. Chem.* **43,** 539–555; 10.1002/jcc.26812 (2022).

20. Lu, T. & Chen, F. Multiwfn: a multifunctional wavefunction analyzer. *J. Comput. Chem.* **33,** 580–592; 10.1002/jcc.22885 (2012).

21. Humphrey, W., Dalke, A. & Schulten, K. VMD: visual molecular dynamics. *J. Mol. Graph.* **14,** 33-8, 27-8; 10.1016/0263-7855(96)00018-5 (1996).

22. LeSuer, R. J., Buttolph, C. & Geiger, W. E. Comparison of the conductivity properties of the tetrabutylammonium salt of tetrakis(pentafluorophenyl)borate anion with those of traditional supporting electrolyte anions in nonaqueous solvents. *Anal. Chem.* **76,** 6395–6401; 10.1021/ac040087x (2004).

23. Menges, F. *Spectragryph - optical spectroscopy software* (2022).

24. Farrugia, L. J. WinGX suite for small-molecule single-crystal crystallography. *J. Appl. Crystallogr.* **32,** 837–838; 10.1107/S0021889899006020 (1999).

25. Sheldrick, G. M. Crystal structure refinement with SHELXL. *Acta Cryst. C* **71,** 3–8; 10.1107/S2053229614024218 (2015).

26. Sheldrick, G. M. SHELXT - Integrated space-group and crystal-structure determination. *Acta Cryst. A* **71,** 3–8; 10.1107/S2053273314026370 (2015).

27. Dolomanov, O. V., Bourhis, L. J., Gildea, R. J., Howard, J. A. K. & Puschmann, H. OLEX2 : a complete structure solution, refinement and analysis program. *J. Appl. Crystallogr.* **42,** 339–341; 10.1107/S0021889808042726 (2009).

28. Crystal Impact - Dr. H. Putz & Dr. K. Brandenburg GbR. *Diamond - Crystal and Molecular Structure Visualization* (2014).
