# Supplementary material for: Isolation of kinetically-stabilised diarylchalcogenide radical cations
Source: Commun Chem. 2025 Aug 9;8:239. doi: 10.1038/s42004-025-01613-z (PMC12335450; doi:10.1038/s42004-025-01613-z)
Supplement: Supplementary file 2 — Description of Additional Supplementary Files [file 42004_2025_1613_MOESM2_ESM.docx]

Description of Additional Supplementary Files

**File name: Supplementary Data 1**

**Description:** Source data for cyclic voltammograms.

**File name: Supplementary Data 2**

**Description:** Source data for UV-Vis-NIR Spectra.

**File name: Supplementary Data 3**

**Description:** Cartesian coordinates of the optimized DFT models.

**File name: Supplementary Data 4**

**Description:** Crystallographic Information File of reported molecular structures.
